# Supplementary material for: Sex-stratified genetic regulators of cytokine production in the Dutch and Tanzanian populations
Source: HGG Adv. 2026 Mar 18;7(2):100593. doi: 10.1016/j.xhgg.2026.100593 (PMC13071459; doi:10.1016/j.xhgg.2026.100593)
Supplement: Document S1. Figures S1–S16 [file mmc1.pdf]

HGGA, Volume 7

Supplemental information

Sex-stratified genetic regulators of cytokine production in the Dutch and Tanzanian populations

Caroline Amour, Raul Cetatean, Isis Ricano Ponce, Nick Keur, Godfrey S. Temba, Vesla I. Kullaya, Blandina T. Mmbaga, Reginald Kavishe, Leo A.B. Joosten, Mihai G. Netea, Quirijn de Mast, Collins K. Boahen, and Vinod Kumar

Supplemental Figures

A

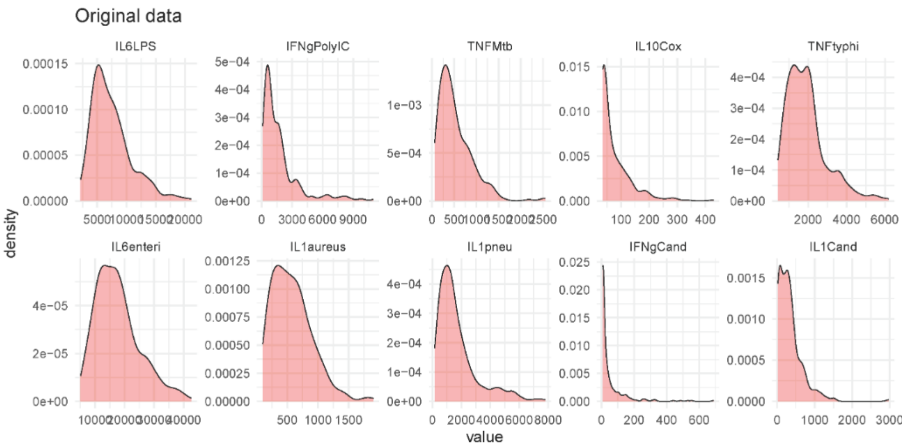

B

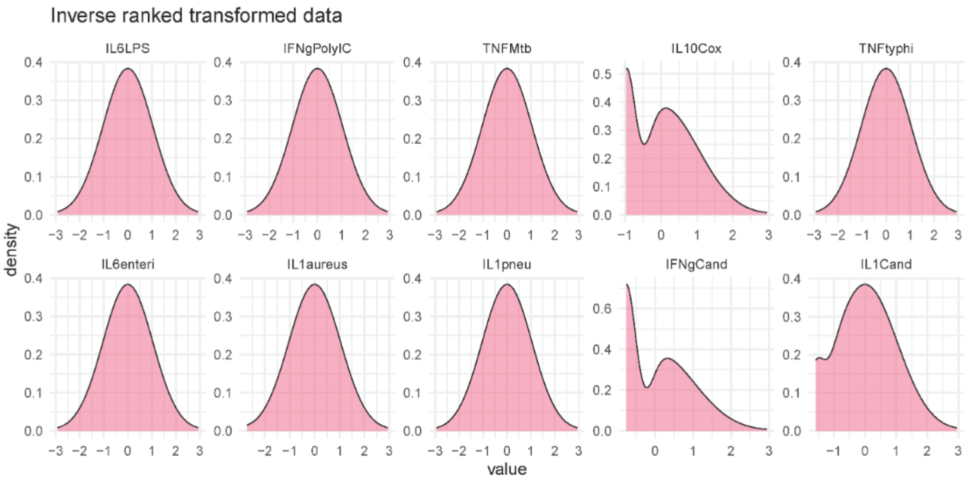

Figure S1: Distribution of the measured cytokine stimuli pairs for the Tanzania cohort.

Density plots depicting the distributions of cytokine production upon stimulation of various bacteria and pathogens. The density plots are arranged as follows A) raw (Original data), and B) inverse-ranked normalization of the cytokine production distributions respectively.

A

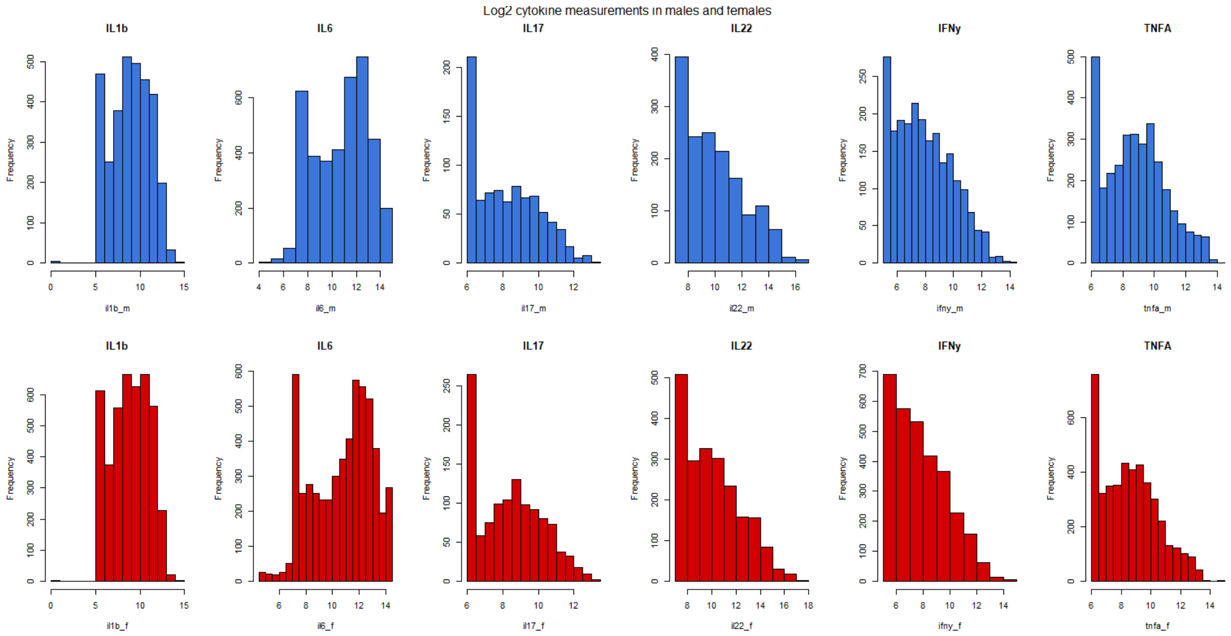

B

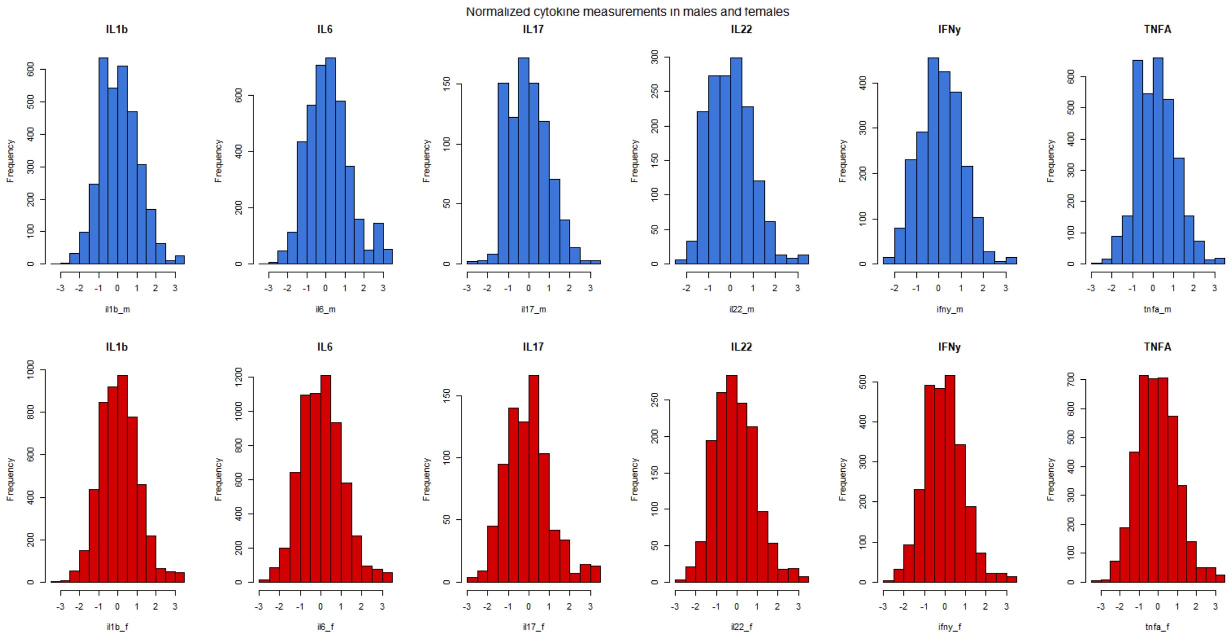

Figure S2: Distribution of the measured cytokine stimuli pairs for the Dutch cohort.

Density plots depicting the distributions of cytokine production upon stimulation of various bacteria and pathogens. The density plots are arranged as follows: A) raw (Original data), and B) inverse-ranked normalization of the cytokine production distributions respectively.

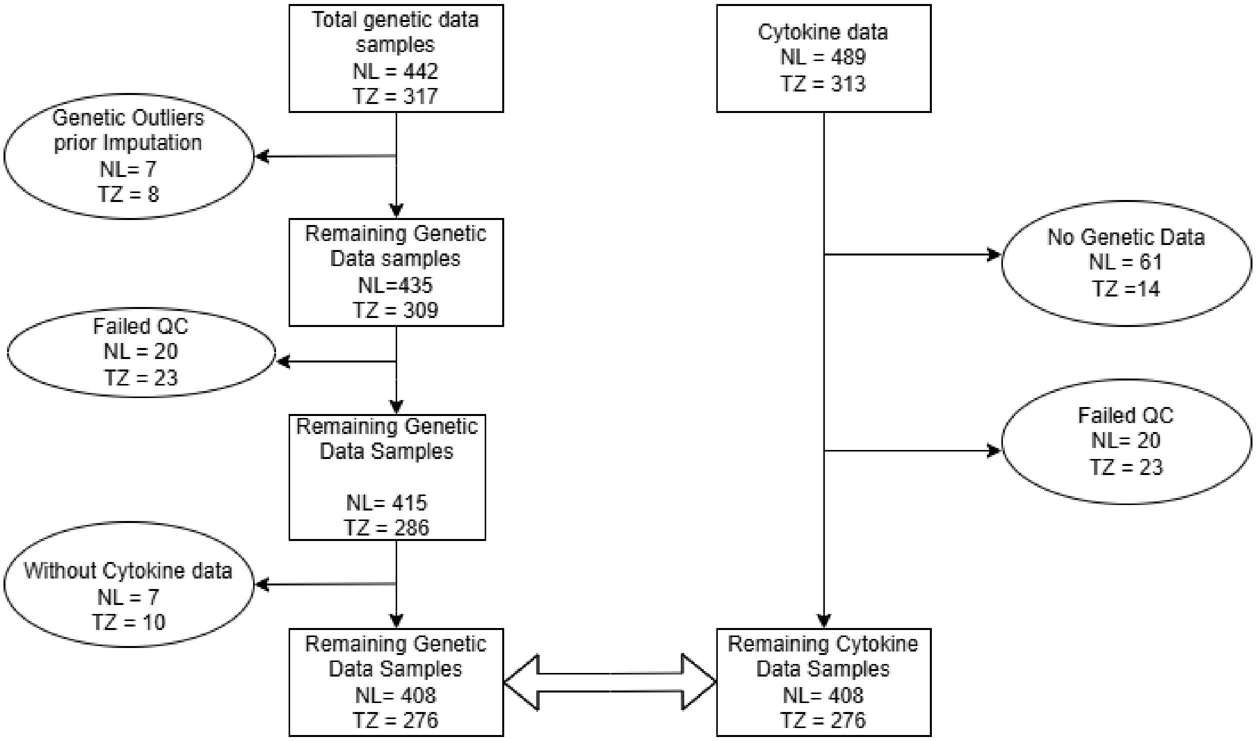

Figure S3: Visualization of samples in the Dutch (NL) and Tanzania (TZ) cohorts

Schematic diagram showing the sample preprocessing according to the measured genetic and cytokine data

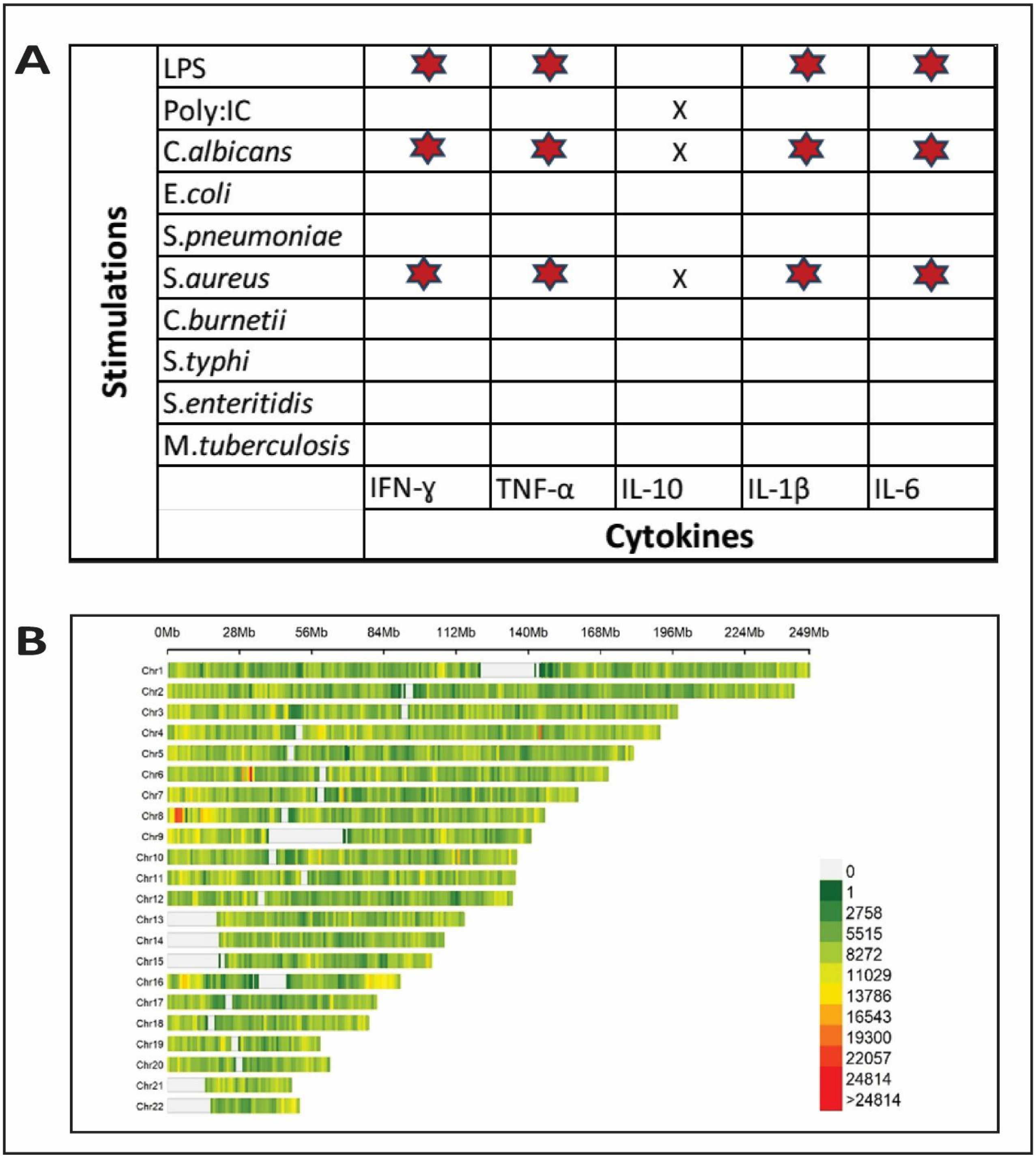

Figure S4: Cytokine-stimulation combinations and SNP density plot

(A) Illustration of cytokine-stimulation combinations common in both African and European datasets (indicated by red stars). Cells with "X'' labels denote cytokine-stimulation pairs excluded from downstream analysis, as more than 75% of samples had values below the detection limit of the assay.

(B) The SNP density plot chromosome wise representing number of SNPs within 1 Mb window size. The horizontal axis shows the chromosome length (Mb); the different color depicts SNP density

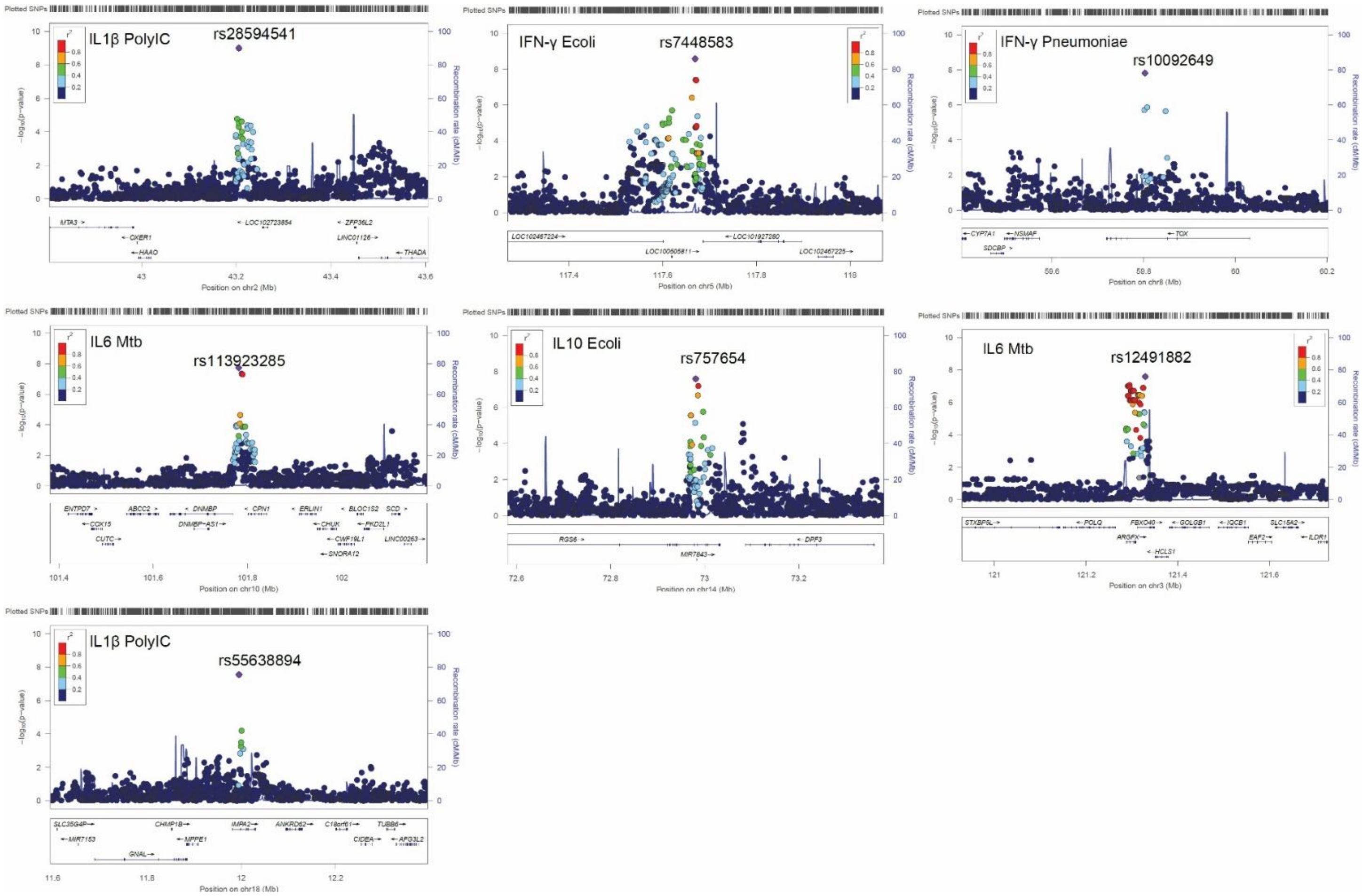

Figure S5: Regional association plot for each genome-wide cytokine QTL in the Tanzanian cohort males. Other SNPs within a genomic window of 400 kb are color-coded based on their linkage disequilibrium (r2) with the top SNP, displayed in purple. The horizontal axis represents chromosomal positions according to the NCBI human genome build 37, and the vertical axis represents -log10 p-values.

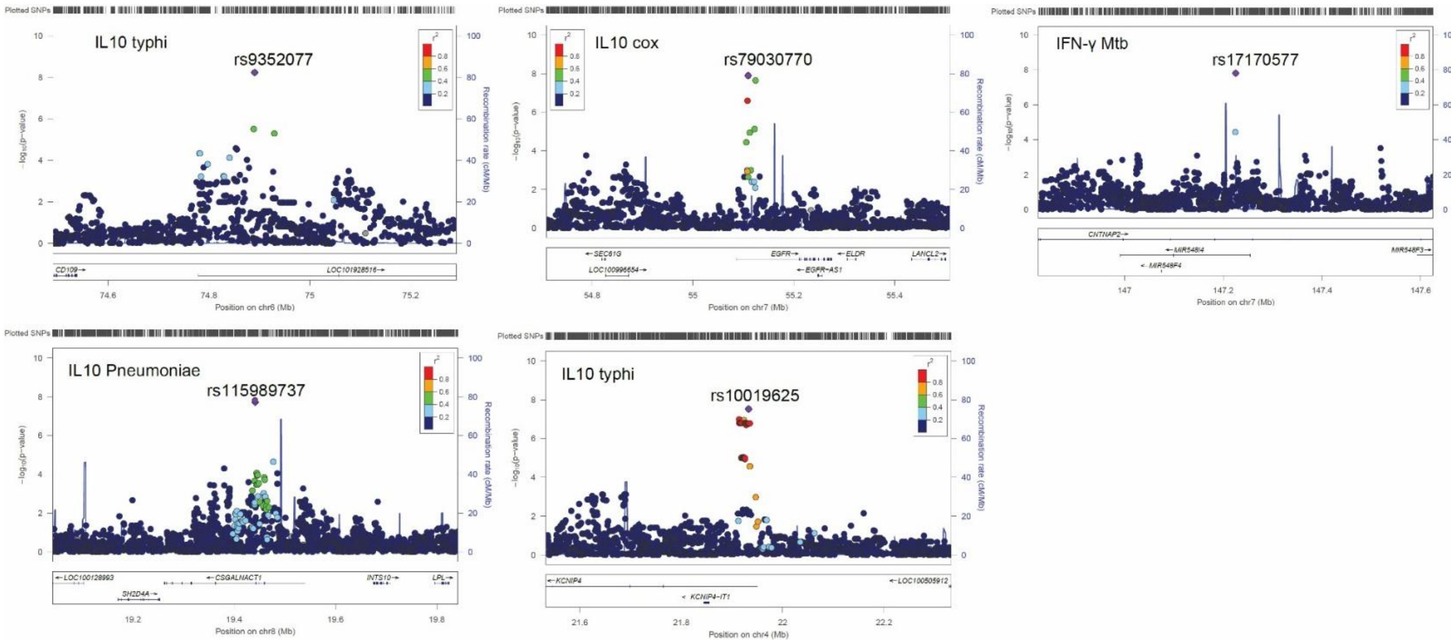

Figure S6: Regional association plot for each genome-wide cytokine QTL in the Tanzanian cohort females. Other SNPs within a genomic window of 400 kb are color-coded based on their linkage disequilibrium (r2) with the top SNP, displayed in purple. The horizontal axis represents chromosomal positions according to the NCBI human genome build 37, and the vertical axis represents -log10 p-values.

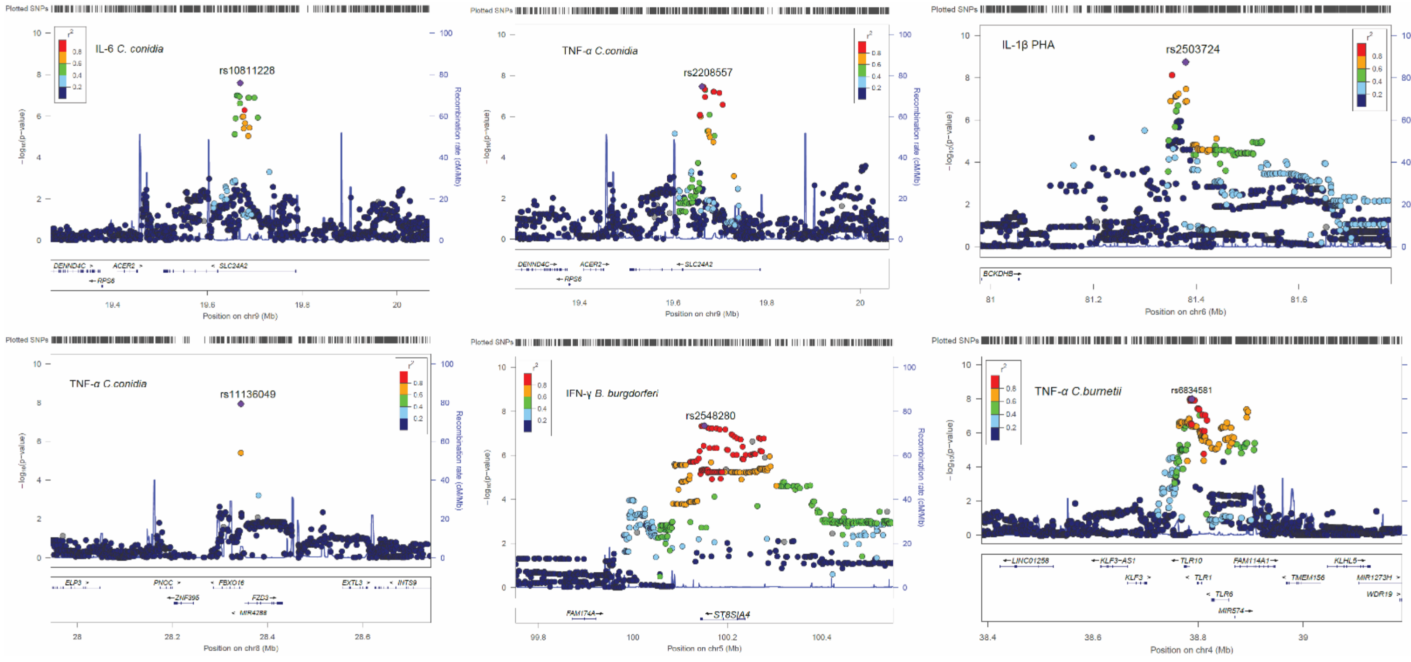

Figure S7: Regional association plot for each genome-wide cytokine QTL in the Dutch cohort males. Other SNPs within a genomic window of 400 kb are color-coded based on their linkage

disequilibrium (r2) with the top SNP, displayed in purple. The horizontal axis represents chromosomal positions according to the NCBI human genome build 37, and the vertical axis represents -log10 p-values.

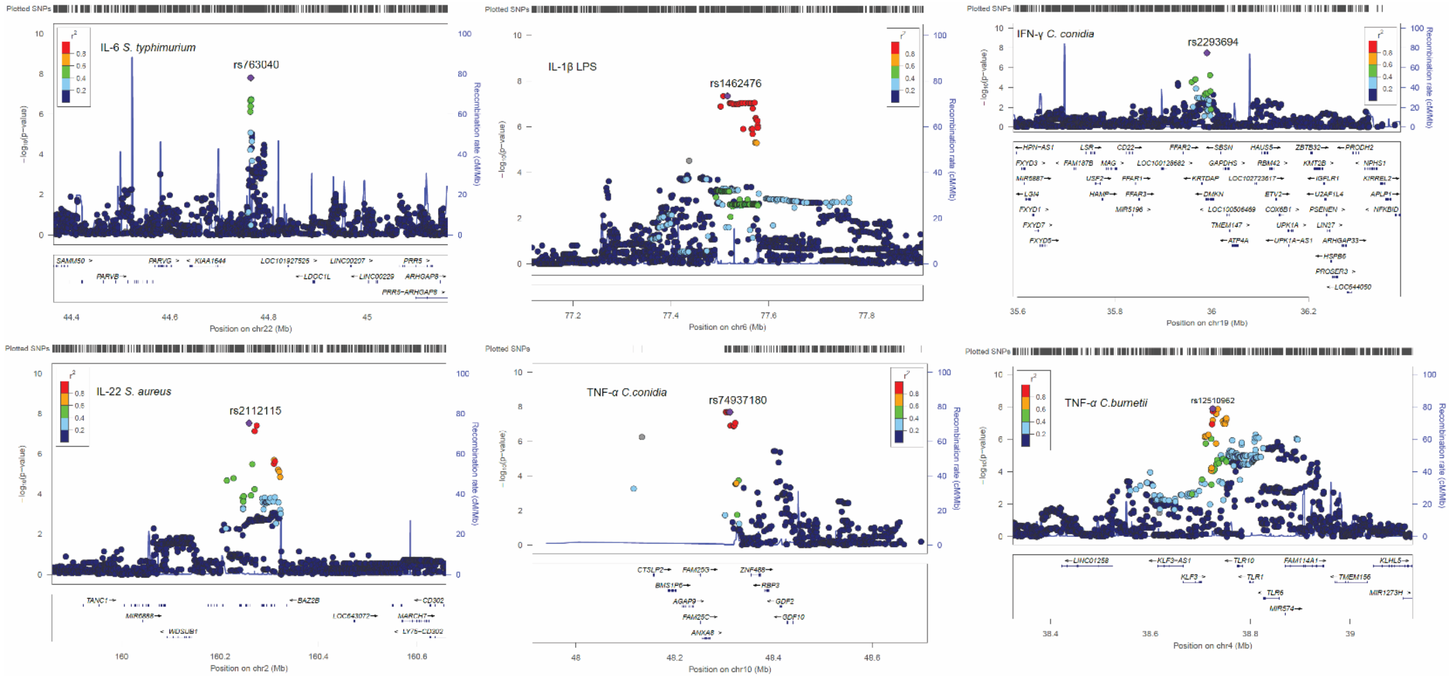

Figure S8: Regional association plot for each genome-wide cytokine QTL among females in the Dutch cohort. Other SNPs within a genomic window of 400 kb are color-coded based on their linkage disequilibrium (r2) with the top SNP, displayed in purple. The horizontal axis represents chromosomal positions according to the NCBI human genome build 37, and the vertical axis represents -log10 p-values.

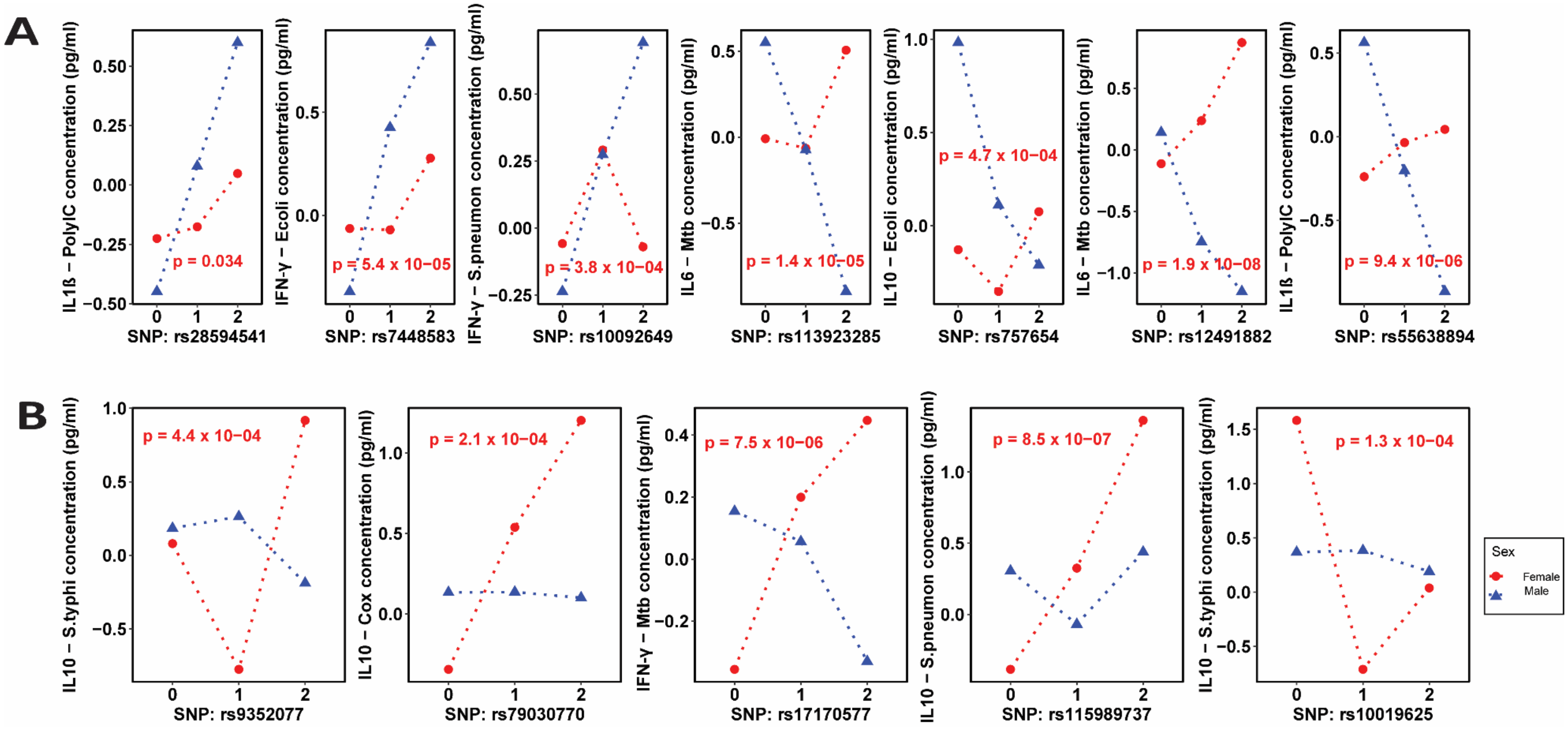

Figure S9: Visualization of Sex-by-SNP interaction in the Tanzanian cohort

(A) Interaction plots of the male-specific genome-wide significant cQTL variants.

(B) Interaction plots of the female-specific genome-wide significant cQTL variants. The median values of the cytokines are depicted with circle and triangle points for males and females respectively.

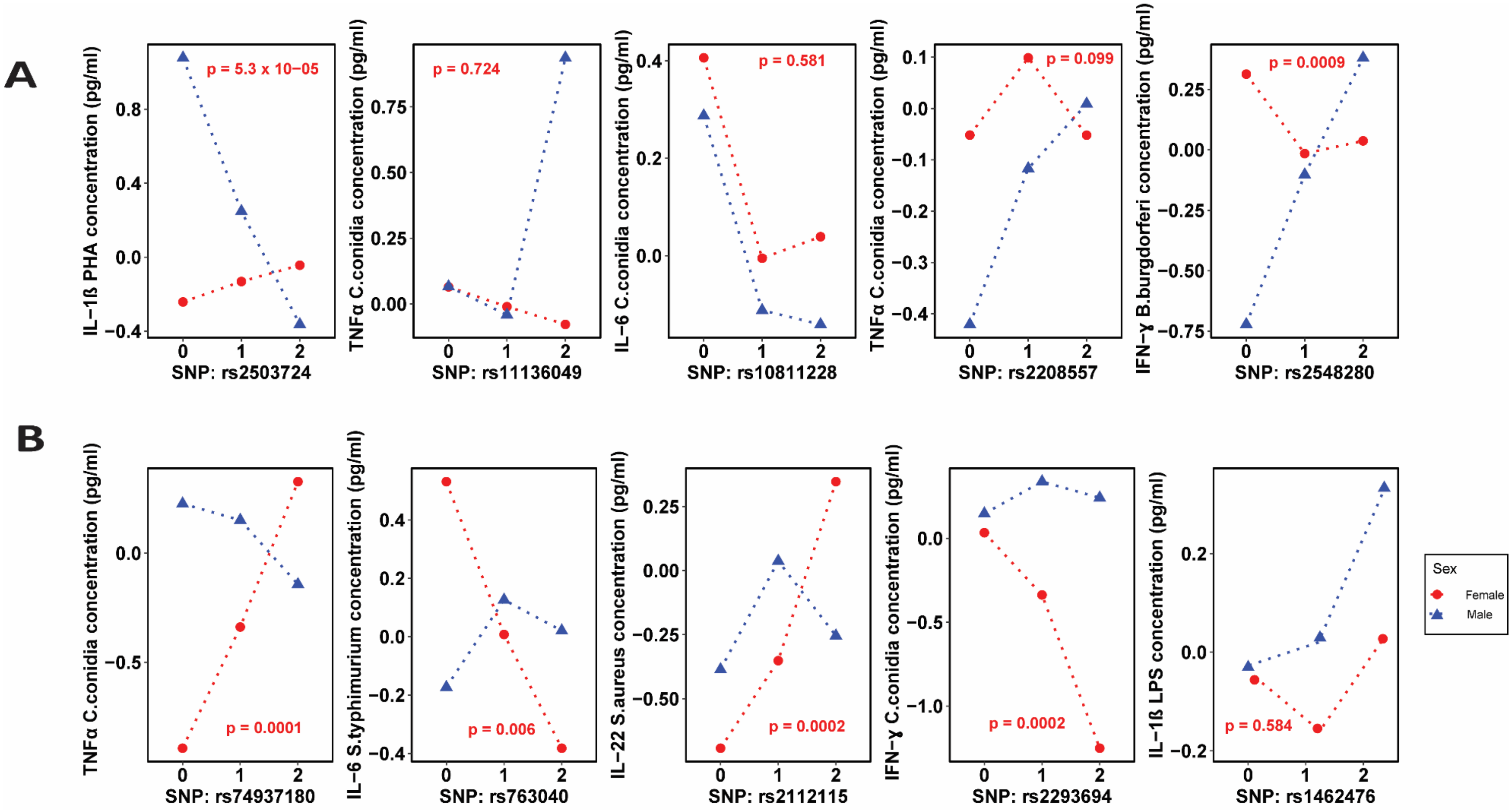

Figure S10: Visualization of Sex-by-SNP interaction in the Dutch cohort

(A) Interaction plots of the male-specific genome-wide significant cQTL variants.

(B) Interaction plots of the female-specific genome-wide significant cQTL variants. The median values of the cytokines are depicted with circle and triangle points for males and females respectively.

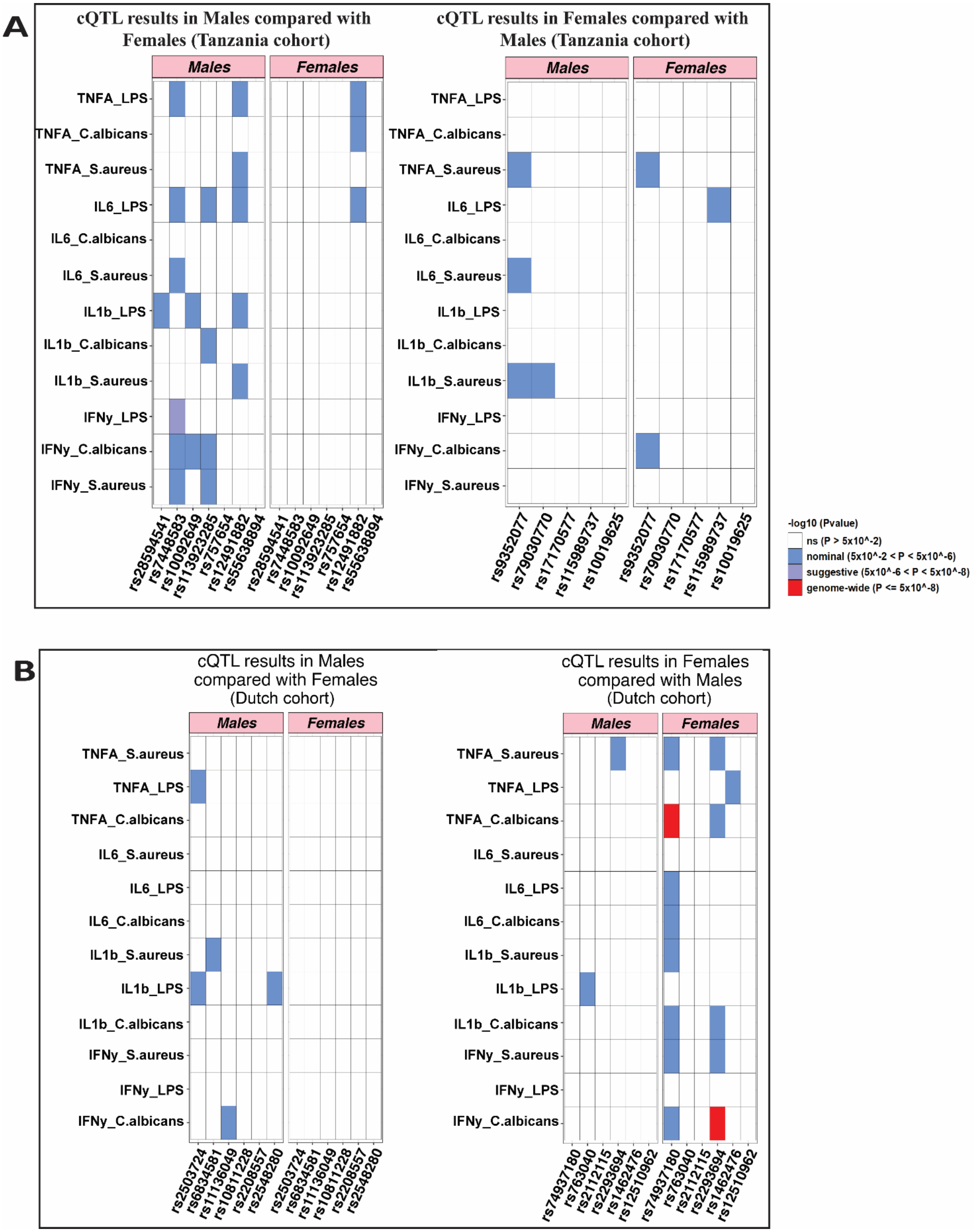

Figure S11: The association results of the cQTLs with the common cytokine measurements between the two cohorts are shown for A) Tanzania and B) Dutch cohorts. The color key indicates the range of cQTL p values (shown as -log10 Pvalue). The x axis shows the genome-

wide significant loci, and the y axis shows the cytokine-stimuli pairs. For each SNP, the -log10(p-value) is shown both for males and for females. Color-key ranges are: not significant - white, nominal - blue, suggestive – grey, and all the genome-wide significant associations are shown in red.

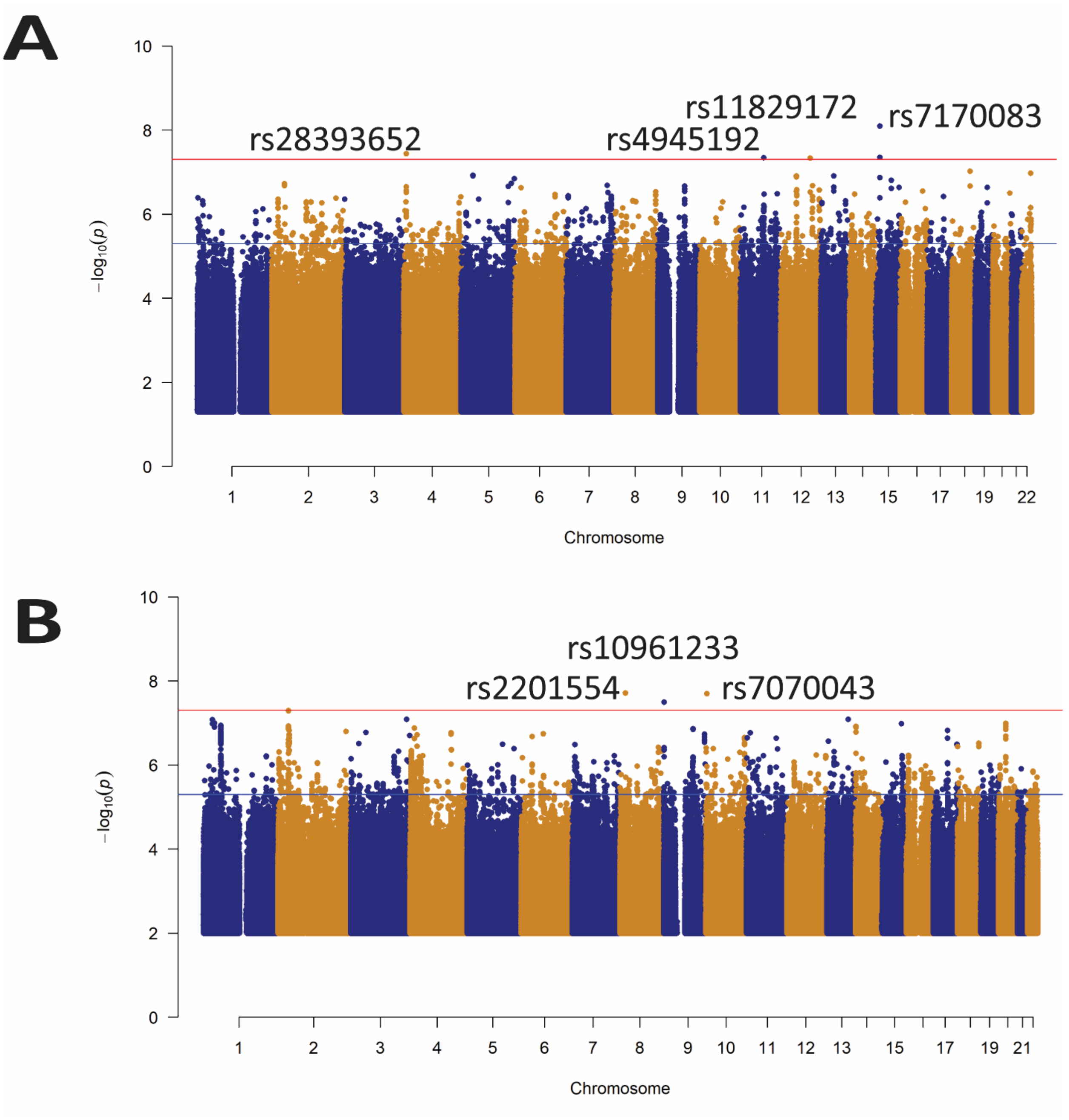

Figure S12: Manhattan plots of the c-QTL variants joint analysis of males and females in the Tanzania and Dutch cohorts.

The Manhattan plots show cQTLs that reached genome-wide significance in A) Participants upon stimulation in the Tanzania cohort and B) Participants in the Dutch cohort. The red horizontal dashed line represents the genome-wide significant threshold (p value < 5×10^{-8})

and the blue dashed line denotes the suggestive evidence of association threshold. Nominally significant (p value < 5 x 10^{-2}) cQTLs are plotted.

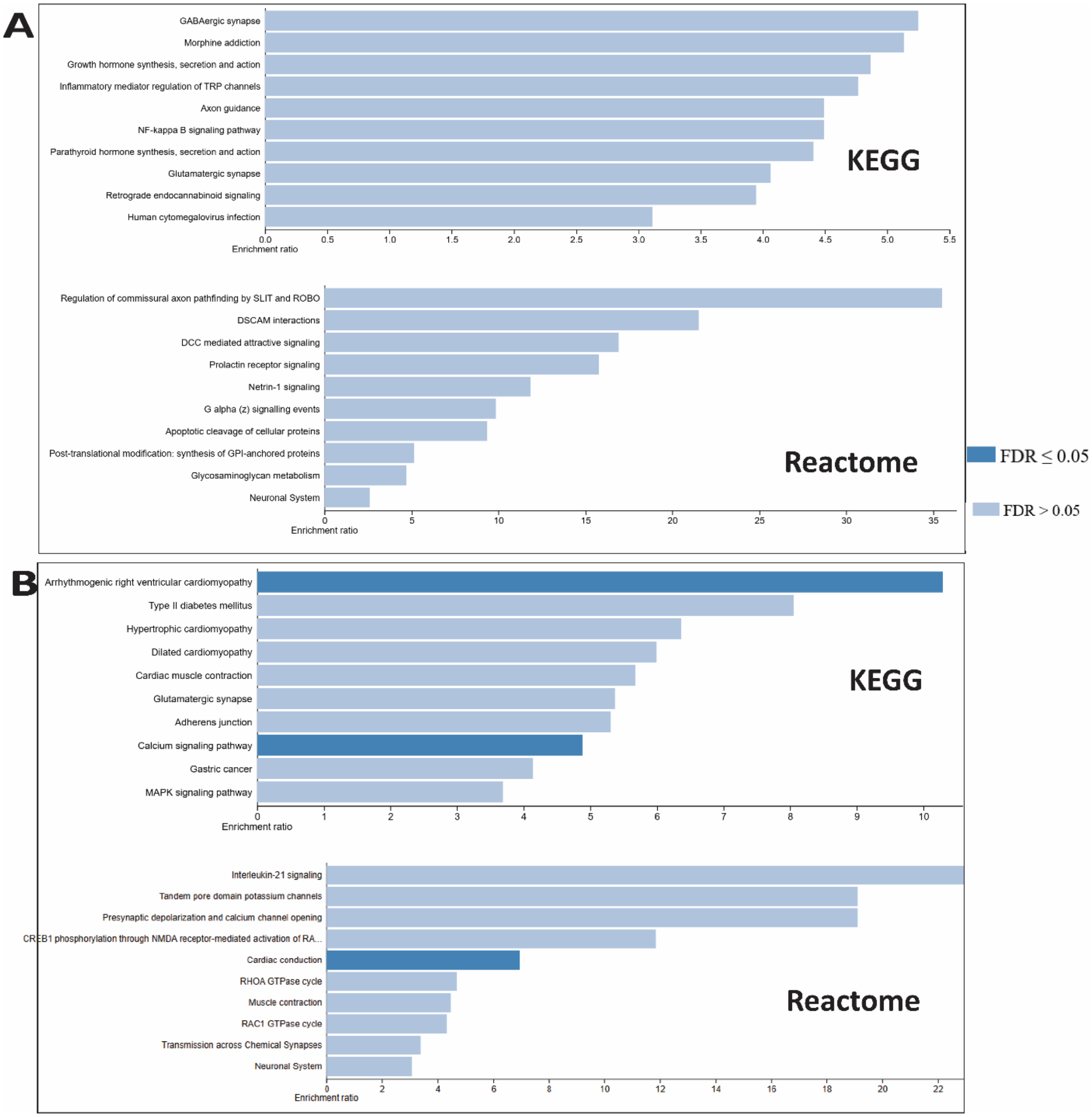

Figure S13: Bar graphs summarizing pathway enrichment results in the Tanzanian cohort

(A) Top enriched KEGG and Reactome pathways for gene sets curated from SNPs associated with cytokines in males.
(B) Top enriched KEGG and Reactome pathways for gene sets curated from SNPs associated with cytokines in females.

The bars represent statistically significant pathways after multiple testing. We set the false discovery rate (FDR)-adjusted p-value to 0.05 as the significance level.

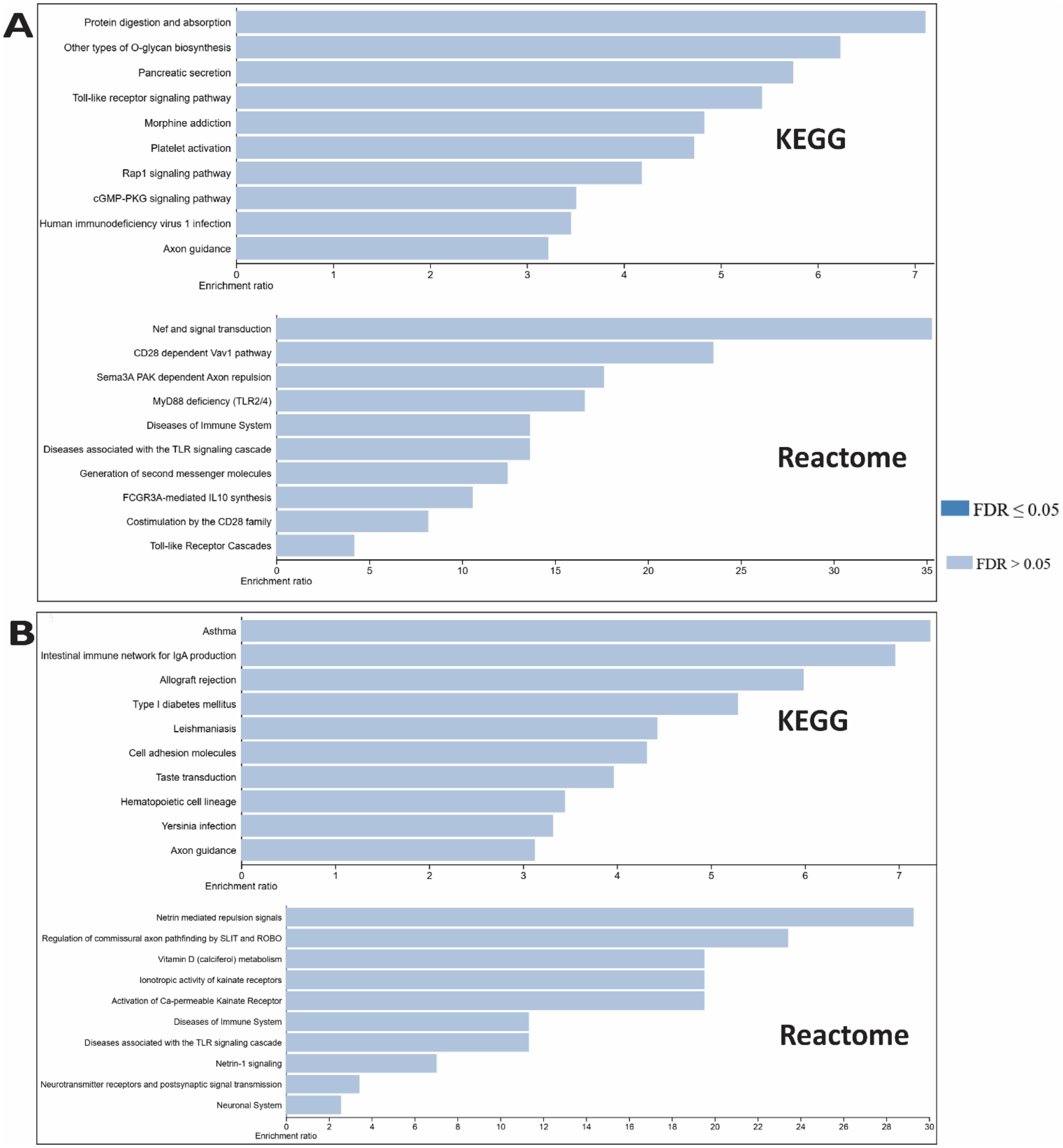

Figure S14: Bar graphs summarizing pathway enrichment results in the Dutch cohort

(A) Top enriched KEGG and Reactome pathways for gene sets curated from SNPs associated with cytokines in males.

(B) Top enriched KEGG and Reactome pathways for gene sets curated from SNPs associated with cytokines in females. The bars represent statistically significant pathways after multiple testing. We set the false discovery rate (FDR)-adjusted p-value to 0.05 as the significance level.

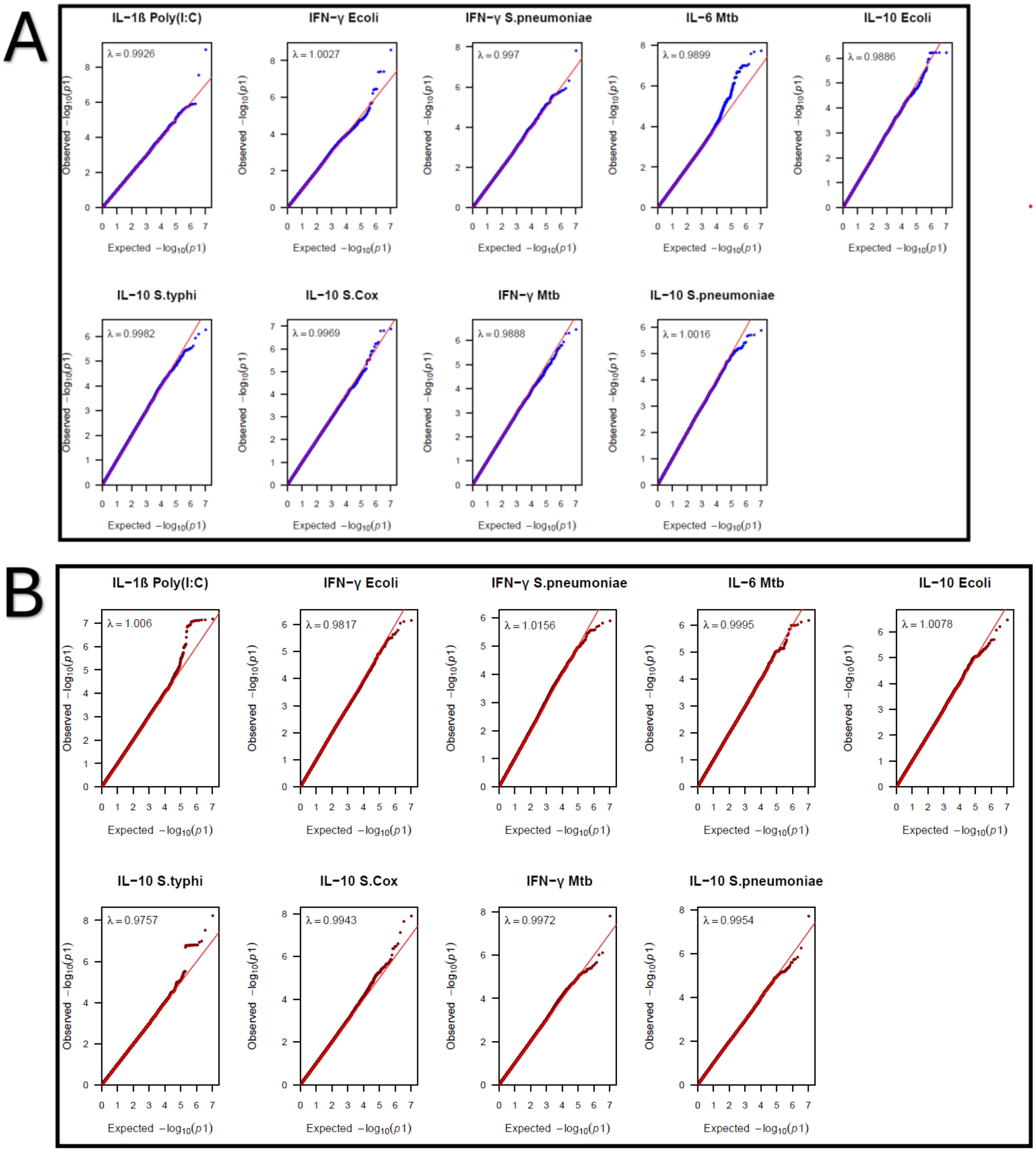

Figure S15: Quantile-quantile (Q-Q) plots for the Tanzania cohort

Quantile-quantile (Q-Q) plots for the pQTL mapping results in the Tanzania cohort for Males (A) and Females (B). The p-values distribution of the analysis (significant cytokine-stimuli) results are shown in blue and dark red colors for the Males and Females respectively.

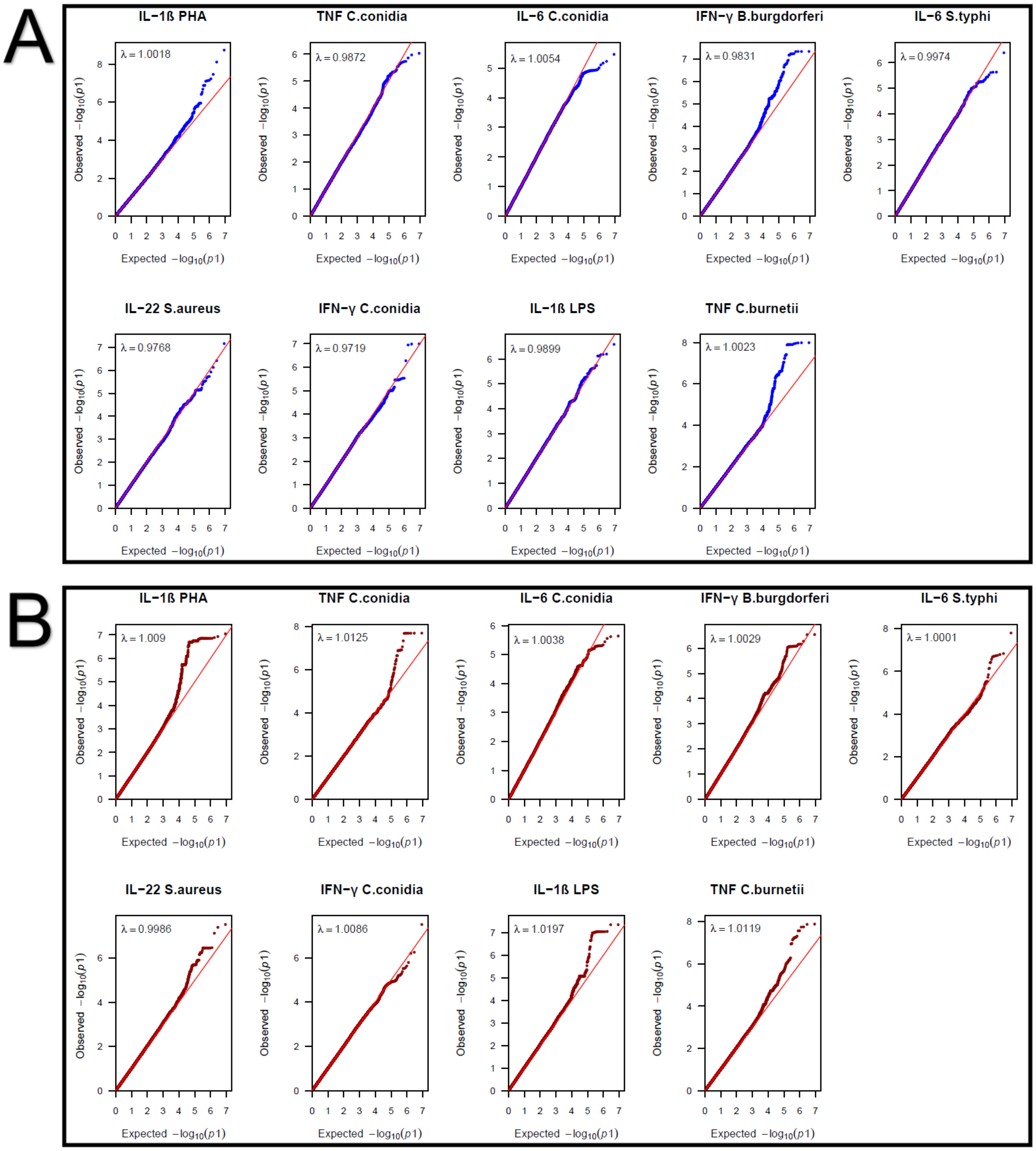

Figure S16: Quantile-quantile (Q-Q) plots for the Dutch cohort

Quantile-quantile (Q-Q) plots for the pQTL mapping results in the Dutch cohort for Males (A) and Females (B). The p-values distribution of the analysis (significant cytokine-stimuli) results are shown in blue and dark red colors for the Males and Females respectively.
