# Supplementary material for: Sex-stratified genetic regulators of cytokine production in the Dutch and Tanzanian populations
Source: HGG Adv. 2026 Mar 18;7(2):100593. doi: 10.1016/j.xhgg.2026.100593 (PMC13071459; doi:10.1016/j.xhgg.2026.100593)
Supplement: Document S3. Article plus supplemental information [file mmc3.pdf]

HGG Advances

ARTICLE

Sex-stratified genetic regulators of cytokine production in the Dutch and Tanzanian populations

Caroline Amour,[1,2] Raul Cetatean,[1] Isis Ricano Ponce,[1] Nick Keur,[1] Godfrey S. Temba,[1,3] Vesla I. Kullaya,[3,5] Blandina T. Mmbaga,[5,6] Reginald Kavishe,[3] Leo A.B. Joosten,[1,7] Mihai G. Netea,[1,4] Quirijn de Mast,[1] Collins K. Boahen,[1,9] and Vinod Kumar[1,8,9,10,*]

Summary

Differences in immune responses are observed between males and females, influenced by genetic, hormonal, and environmental factors. The sex-specific genetic effects on cytokine production, however, remain underexplored. This study aimed to identify sex-specific quantitative trait loci (QTLs) affecting cytokine production in response to diverse infectious antigens. We performed sex-stratified cytokine QTL (cQTLs) mapping in two population-based cohorts from Tanzania and the Netherlands. In the Tanzanian cohort, 12 genome-wide significant cytokine QTLs were identified, with 7 observed in males and 5 in females. In the Dutch cohort, 12 genome-wide significant cQTLs were identified, with 6 cQTLs each in males and females. Colocalization analysis confirmed that all 12 genome-wide cQTLs from the Tanzanian cohort are sex specific, while in the Dutch cohort 10 genome-wide cQTL variants are modulated in a sex-specific manner. Furthermore, pathway and phenotype enrichment analyses linked the identified cQTL variants to relevant immune functions and sex-biased traits. Our study highlights the importance of sex-stratified genetic analyses when investigating the genetic basis of cytokine production in humans. We show that sex-specific cQTLs may underlie observed phenotypic differences between males and females and that accounting for such effects can inform the development of personalized medical treatments for sex-biased diseases.

Introduction

Sex differences in immune responses are well documented, with females generally exhibiting stronger adaptive immunity than males.[1] This contributes to lower susceptibility to certain infectious diseases, including Dengue virus, hepatitis B, hepatitis C, and SARS-CoV-2.[2–4] Conversely, certain viruses such as influenza tend to affect more females than males.[5] These differences extend beyond infectious diseases to include autoimmune diseases, where approximately 80% of the affected individuals are females,[6] and non-reproductive cancers, which are more common in males.[7] These differences arise from complex interactions between genetic, hormonal, and environmental factors, underscoring the need to dissect sex-specific mechanisms of immune regulation to better understand disease risk.

Genetic variation plays an important role in shaping these sex differences. Recent studies have shown that common genetic variants exert different effects in males and females,[8,9] alongside sex-biased gene expression[10–17] and hormonal differences.[9,18] Previous studies have investigated differences in genetic variation contributions to a single trait (heritability [h^2]),[19–22] genetic variants affecting traits differently (deviation of genetic correlations from 1 [r_g]),[19,21,23–26] and performed sex-stratified genome-wide association studies (GWASs) to directly assess differences in the effects of genetic variants between the sexes.[13,27–34] Despite these advances, the contribution of genetic variation to sex-specific regulation of cytokine responses remain insufficiently characterized. This is particularly relevant for pathogen-induced cytokine production, where sex-agnostic analyses may mask important differences. Understanding these mechanisms is essential, as sex-specific genetic effects may partly explain the observed differences in susceptibility to infections, autoimmune diseases, and treatment outcomes.[24,35]

Cytokines are key modulators of immune responses, with an important role in the pathophysiology of autoimmune, inflammatory, neurodegenerative diseases,[36] cancer,[37] and infections.[38] Studying cytokines across diverse populations, including from different continents, provides insight into potential differences in immune responses and genetic predispositions. The Human Functional Genomics Project (HFGP) was established to systematically map host genetic and non-genetic factors

[1]Department of Internal Medicine, Radboudumc Community for Infectious Diseases, Radboud University Medical Center, Nijmegen, the Netherlands; [2]Institute of Public Health, Kilimanjaro Christian Medical University College (KCMUCo), Moshi, Tanzania; [3]Department of Medical Biochemistry and Molecular Biology, Kilimanjaro Christian Medical University College (KCMUCo), Moshi, Tanzania; [4]Department of Immunology and Metabolism, Life & Medical Sciences (LIMES) Institute, University of Bonn, Bonn, Germany; [5]Kilimanjaro Clinical Research Institute, Kilimanjaro Christian Medical Center, Moshi, Tanzania; [6]Department of Pediatrics, Kilimanjaro Christian Medical University College (KCMUCo), Moshi, Tanzania; [7]Department of Medical Genetics, Iuliu Hatieganu University of Medicine and Pharmacy, Cluj-Napoca, Romania; [8]Nitte (Deemed to Be University), Medical Sciences Complex, Nitte University Centre for Science Education and Research (NUCSER), Deralakatte, Mangalore 575018, India
[9]These authors contributed equally
[10]Lead contact
*Correspondence: v.kumar@radboudumc.nl
https://doi.org/10.1016/j.xhgg.2026.100593.
© 2026 The Authors. Published by Elsevier Inc. on behalf of American Society of Human Genetics.
This is an open access article under the CC BY license (http://creativecommons.org/licenses/by/4.0/).

influencing the immune response variability (http://www.humanfunctionalgenomics.org/site/). A previous HFGP study showed important differences in genetic regulation of cytokine responses between Western European and Eastern African cohort.[39]

Building on this, the present study aimed to identify sex-specific genetic variants that influence cytokine production in response to a broad range of infectious antigens. Using genome-wide single-nucleotide polymorphisms (SNPs) and cytokine production data from the Netherlands (500FG) and Tanzania (300TZFG) cohorts,[39–41] we performed sex-stratified cytokine quantitative trait loci (cQTL) mapping, colocalization, and pathway enrichment analyses. By comparing findings across two ancestries, we provide insights into the sex-specific genetic architecture of cytokine regulation, with implications for understanding sex-biased disease susceptibility and informing personalized medicine.

Subjects and methods

Study cohort characteristics

The study utilized data from two cohorts: the Tanzania Functional Genomics and a Dutch cohort of individuals of Western European origin. Detailed demographic characteristics of these cohorts have been previously described.[42,43] In summary, the 500FG cohort consists of around 534 healthy individuals (237 males, 296 females) of Western European ancestry, aged 18–75 years. This cohort is part of the HFGP (http://www.humanfunctionalgenomics.org/site/). Exclusion criteria were: the use of any medication in the past month and acute or chronic diseases at the time of blood sampling. Pregnant, postpartum, or breastfeeding females were excluded.

The Tanzanian cohort comprised 323 healthy individuals (159 males, 164 females) aged 18–65 years, residing in the Kilimanjaro region of Northern Tanzania. All participants tested negative for malaria and HIV and were free from any acute or chronic diseases. The exclusion criteria included pregnancy, use of antibiotics or anti-malaria medication in the past 3 months, tuberculosis in the past year, blood pressure above 140/90 mmHg or below 90/60 mmHg, and random blood glucose levels greater than 8.0 mmol/L. A total of 317 and 324 individuals in the Tanzanian cohort had genotype and cytokine data, respectively.

Sample collection and stimuli

500FG (Dutch) cohort

All stimuli and sample collection procedures have been described previously in detail.[42] Supplementary notes summarize all the stimuli used for analysis. Informed consent was obtained before collecting venous blood from the volunteers. Peripheral blood mononuclear cells were obtained by density centrifugation of the EDTA blood diluted 1:1 in pyrogen-free saline over Ficoll-Paque (Pharmacia Biotech, Uppsala). Cells were successively washed twice in saline and suspended in a medium (RPMI 1640) supplemented with gentamicin (10 mg/mL), L-glutamine (10 nM), and pyruvate (10 mM). Cell counting was performed using a Coulter counter (Beckman Coulter, Pasadena), and the cell number was adjusted to 5×10^6 cells/mL.

Monocytes were cultured in flat-bottom plates with 10% human serum at 37°C and 5% CO_2 for 6 days. After the differentiation, the medium was removed, and the differentiated macrophages were stimulated for 24 h. In a 48-well plate, 100 μL of heparin blood was placed and subsequently stimulated with a 400 μL stimulus, resulting in a final volume of 500 μL. The stimulation process lasted for 48 h at 37°C and 5% CO_2.

300TZFG (Tanzania) cohort

As previously described,[43] *ex vivo* cytokine-stimulation experiments were performed at the biotechnology laboratory of Kilimanjaro Clinical Research Institute in Moshi, Tanzania. Whole blood was stimulated with 10 stimuli (see Table S16 from Temba et al.[43] for further details) including bacterial and fungal pathogens as well as TLR3 and TLR4 agonists. One hundred microliters of heparin blood was added to a 48-well culture plate and subsequently stimulated with 400 μL of stimulus for 48 h at 37°C and 5% CO_2. Stimuli were prepared in RPMI culture medium (Dutch modified, Invitrogen) supplemented with 50 μg/mL gentamicin, 2 mM GlutaMAX, and 1 mM pyruvate. Supernatants were collected and stored at −80°C until used for ELISA. To minimize variation between measurements, all samples were analyzed using kits from the same lot number.

Genotyping, quality control, and imputation of genetic data

Genotyping, imputation and quality control of the data have been previously described for both Dutch[42] and Tanzania[39] cohorts. For the Dutch cohort, DNA samples from approximately 500 individuals were genotyped using the commercially available Illumina Human Omni Express-8 v.1.0 SNP chip. Opticall 0.7.0 with default settings was used to perform genotype calling.[44] Variants with Hardy-Weinberg equilibrium (HWE) < 0.0001, minor allele frequency (MAF) < 0.001 and call rate ≤ 0.99 were excluded during quality control. Quality control measures included checks for sex discrepancies, cryptic relatedness, and population stratification to exclude genetic outliers (17 in the 500FG cohort). Genotyped samples were imputed with the Michigan Imputation server,[45] with the Genome of the Netherlands Consortium, (GoNL 2014) and the human reference consortium (HRC r1.1 2016) as reference panels for the 500FG cohort. We filtered out genetic variants with imputation quality score (R^2) < 0.3 and MAF cutoff of 5%. A total of 4,358,039 SNPs was available for the 500FG cohort.

For the Tanzanian cohort, DNA was extracted from whole blood using the DNeasy kit. Genotyping was performed with the Global Screening Array (GSA) SNP chip. We used default settings of Opticall 0.7021 to perform genotype calling. Quality control filters prior to imputation include excluding variants with call rate exceeding 0.1, low minor allele frequencies (MAFs < 0.001), and SNPs deviating from HWE with $p < 1 \times 10^{-4}$. Specifically, filters were applied simultaneously for MAF < 0.001, HWE ($p < 1 \times 10^{-4}$), and SNP missingness (call rate < 0.9, i.e., >10% missing genotypes). The GSA SNP chip initially generated over 5 million variants. Following the application of these quality control filters, 409,261 SNPs remained for imputation. Next, we excluded 15 samples that were potential genetic outliers through identification by using multidimensional scaling plots. Strand alignment to a reference panel, 1000 Genomes reference panel dataset, was performed via Genotype harmonizer.[46] We performed genotype imputation for all autosomal chromosomes by using the Minimac4 software through the publicly available Michigan Imputation server.[45] The Human Reference Consortium (HCR r.1.1 2016) was used as a reference

panel and the dataset was phased with Eagle v.2.3. Variants with imputation quality score (R^2) < 0.3 were excluded from further analysis.

Genotyping and imputation generated a total of 5,271,779 variants from 308 individuals. We further excluded samples because of extreme heterozygosity rates and cryptic relatedness. No sample was removed as a result of incorrect or ambiguous sex information when compared with self-reported sex in the phenotype data. We used a multidimensional scaling approach to examine potential population structure by merging our data with the 1000 Genomes Project data. We considered SNPs with MAFs > 5% and not deviating from HWE with $p > 1 \times 10^{-6}$, yielding a final dataset of 5,269,992 SNPs that were subsequently used for cytokine QTL analysis.

Measurement and quality control of cytokine data

In the Dutch cohort, cytokine concentrations of IL-1β, IL-6, IL-17, IL-22, IFN-γ, and TNF-α were measured from whole-blood stimulation assays using ELISA kits, following the manufacturer's instructions. In the Tanzanian cohort, concentrations of IL-6, IL-1β, IFN-γ, TNF-α, and IL-10 were measured in stored supernatants via ELISA (R&D Systems; IFN-γ: Sanguin). IL-10 responses to *S. aureus*, *C. albicans*, and poly(I:C) were excluded, as over 75% of the individuals had values below the detection limit, resulting in 47 cytokine-stimulation combinations used in this analysis.

In both cohorts, preprocessing of cytokine data was performed before statistical analysis. Raw cytokine concentrations were first log2-transformed (Figures S1A and S2A) and normalized to follow Gaussian distributions using an inverse rank-based normal transformation (Figures S1B and S2B) function in the GenABEL R package.[47] Unsupervised hierarchical clustering analysis was performed using Pearson's correlation as a measure of similarity to identify potential outliers, confirming uniform clustering across samples.

cQTL mapping

cQTL mapping was performed in a sex-stratified manner using a linear regression model implemented in the Matrix eQTL R package (v.2.3).[48] Genotype and cytokine data were available for 408 individuals (173 males, 235 females) in the Dutch cohort, and 276 individuals (140 males, 136 females) in the Tanzanian cohort (Figure S3). Association analyses were conducted separately by sex and cohort, with linear models adjusted for relevant covariates: age, total leukocyte count, and residential status in the Tanzanian cohort; and age along with absolute counts of B cells, monocytes, lymphocytes, NK cells, and T cells in the Dutch cohort.[42]

In the Dutch cohort, contraceptive use was considered for the female samples, with 0 indicating no contraceptive use and 1 indicating contraceptive use. Furthermore, following normalization, samples were grouped based on stimulation, cell system, and time of stimulation prior to the analysis. To complement the sex-stratified cQTL mapping, we also performed a joint analysis including both males and females. This joint model used the same covariates and statistical framework as the stratified analyses to identify associations shared across sexes as well as to serve as a baseline for comparison with sex-specific effects.

To account for the multiple testing burden, the significant cutoff is determined based on the ratio of the conventional genome-wide significant threshold (5.0×10^{-8}) and the product of the number of cytokine-stimuli pairs (47 and 95 for Tanzanian and Dutch cohorts, respectively). The resulting p values were 1.06×10^{-9} and 5.26×10^{-10} for Tanzanian and Dutch cohorts, respectively. Given that no association surpassed this stringent threshold, we considered the conventional genome-wide significant threshold (5.0×10^{-8}) together with colocalization analysis and SNP-by-sex interaction analysis to identify key loci exhibiting sex-dependent effect.

For genome-wide QTL mapping, linkage disequilibrium (LD) clumping was performed with the greedy algorithm implemented in PLINK[49] to identify independent associations. Our imputed genotype data (Figure S4) served as a reference dataset for estimating LD, clumps around lead SNPs were formed with a default window size of 250 kb, and r^2 threshold greater than 0.1 was applied.

Colocalization analysis

Colocalization analyses were performed on genome-wide significant cQTLs in males and females using the coloc R package, which implements approximate Bayes factors for testing colocalization of two potentially related phenotypes in a genomic region.[50] A 500-kb region around the lead SNPs was tested in both sexes. A posterior probability for H4 (PP4) > 0.75 is considered as a strong indication that the genomic locus is shared between sexes, whereas a low posterior probability suggests that the region is sex specific. Other hypotheses tested are H1 and H2, which indicate either males or females have significant associations in the tested region and H3, which indicates that both males and females have significantly unique causal variants. The colocalization approach defined trait 1 and 2 throughout as males and females, respectively; and H1(PP1) > 0.75 or H2(PP2) > 0.75 was considered as indicator for male- or female-specific loci.

Locus zoom was used to visualize the loci in both sexes,[51] and the plotted regions are 500 kb in length. The LD population selected was the European population for the Dutch cohort and the African population for the Tanzanian cohort.

Phenotype associations

PhenoScanner (v.2)[52,53] was used to identify associations between sex-specific cQTLs, traits, and diseases. For each cytokine-locus association discovered, we divided the locus into segments no larger than 1 Mb (the maximum allowable by PhenoScanner application programming interface), as needed. Next, we inputted the resulting region(s) into the PhenoScanner function in R, specifying "build" as "37," "p value" as 1×10^{-5}, "catalog" as "eQTL," and proxies set as "None" (with a query date of September 21, 2023).

Pathway enrichment analysis

To evaluate the possible biological relevance of cQTLs, we conducted an over-representation analysis, which performs a hypergeometric test to identify causal pathways. We used the Web Gestalt (WEB-based Gene Set Analysis Toolkit), a freely available online tool and one of the most widely used gene set enrichment analysis tools. To identify these pathways, we first prioritized suggestive independent cQTLs ($p < 1 \times 10^{-5}$) for each of the sex-stratified cohorts. The independent lead SNPs (lowest p value per SNP over all traits) are then mapped to genes near these genetic variants by using a window size of 250 kb upstream and downstream of each SNP. We considered pathways as statistically significant only after correcting for multiple testing using the Benjamin-Hochberg method with a false discovery rate threshold of $p < 0.05$.

Table 1. **Genome-wide significant loci identified in the cQTL mapping among the Tanzanians**

Cytokine	SNP	chr	*p* value m	β m	*p* value f	β f	Sex specific	Nearest gene	PP1	PP2	PP3	PP4
IFN-γ *S. pneumoniae*	rs10092649	8	1.6×10^{-8}	0.62	8.6×10^{-1}	−0.026	male	TOX	0.9	0.001	0.0484	0.032
IL6 Mtb	rs113923285	10	1.8×10^{-8}	−0.599	1.8×10^{-1}	0.135	male	CPN1	0.876	0.0005	0.0503	0.065
IL6 Mtb	rs12491882	3	2.6×10^{-8}	−0.778	6.6×10^{-2}	0.224	male	FBXO40	0.886	0.0002	0.0385	0.071
IL-1β Poly(I:C)	rs28594541	2	9.9×10^{-10}	0.587	1.9×10^{-1}	0.177	male	LOC102723854	0.859	9E−05	0.0914	0.049
IL-1β Poly(I:C)	rs55638894	18	2.8×10^{-8}	−0.667	1.8×10^{-1}	0.188	male	IMPA2	0.84	0.002	0.0817	0.056
IFN-γ *E. coli*	rs7448583	5	2.7×10^{-9}	0.667	8.6×10^{-1}	0.022	male	LOC100505811	0.918	0.0001	0.055	0.025
IL10 *E. coli*	rs757654	14	2.6×10^{-8}	−0.557	5.6×10^{-1}	−0.062	male	MIR7843	0.912	0.0006	0.0472	0.029
IL-10 *S. typhi*	rs10019625	4	1.2×10^{-1}	−0.234	3.0×10^{-8}	0.977	female	KCNIP4−IT1	0.0003	0.832	0.0657	0.098
IL-10 *S. pneumoniae*	rs115989737	8	1.3×10^{-1}	−0.259	1.9×10^{-8}	0.853	female	CSGALNACT1	0.0021	0.808	0.0994	0.073
IFN-γ Mtb	rs17170577	7	6.9×10^{-1}	−0.05	1.5×10^{-8}	0.627	female	MIR548I4, CNTNAP2	0.0011	0.872	0.093	0.023
IL10 Cox	rs79030770	7	8.0×10^{-1}	0.038	1.3×10^{-8}	0.862	female	EGFR	0.0008	0.897	0.0531	0.035
IL10 *S. typhi*	rs9352077	6	2.9×10^{-1}	−0.176	5.9×10^{-9}	−1.135	female	CD109, LOC101928516	0.001	0.875	0.0472	0.058

p values for the lead SNP for both sexes are reported in the columns *p* value m and *p* value f.
PP1 indicates the posterior probability that only males have a genetic association in the testing region.
PP2 indicates the posterior probability that only females have a genetic association in the testing region.
PP3 indicates the posterior probability that both sexes have a genetic association in the testing region; however, the causal variants are different.
PP4 indicates the posterior probability that male and female share the same causal variant.

Statistical analyses

All statistical methods and tools used are described under the appropriate sections above. Analysis and visualization were performed in R v.4.5.0 unless otherwise stated. Generalized linear regression methods was performed to examine the potential interaction between SNP genotypes and sex. The lm () function in R was used for conducting the analysis and interaction effects were visualized using the interaction plot () function.

Ethics approval and consent to participate

The study was conducted in accordance with the Declaration of Helsinki, and the Tanzanian study was approved by the Kilimanjaro Christian Medical University College (CRERC) (no. 2443) and the National Institute for Medical Research (NIMR/HQ/R.8a/Vol. IX/2290 and NIMR/HQ/R.8a/Vol.IX/3318) in Tanzania. Similarly, the Dutch cohort was approved by the Ethical Committee of Radboud University Medical Center Nijmegen, the Netherlands (NL42561.091.12, 2012/550). Written informed consent was obtained from all participants involved in the study.

Results

We aimed to identify SNPs associated with cytokine production in response to different pathogens and stimuli profiled in male and female samples separately using two different population-based cohorts.

Sex-stratified QTL mapping identifies 12 genome-wide significant cQTLs in the Tanzanian cohort

To identify sex-specific genetic variants influencing cytokine levels in response to different pathogens and stimuli, we performed cQTL mapping in a sex-stratified manner at genome-wide scale. In the Tanzanian cohort, we identified 12 independent cQTLs at genome-wide significance ($p < 5 \times 10^{-8}$) (Table 1; Figures 1A and 1B). Among males ($n = 140$), seven lead cQTLs (rs28594541, rs7448583, rs10092649, rs113923285, rs757654, rs12491882, and rs55638894) reached genome-wide significance, whereas in females ($n = 136$), five cQTLs (rs9352077, rs79030770, rs17170577, rs115989737, and rs10019625) were significant. The top genome-wide significant SNP in males was SNP rs28594541 on chromosome 2, associated with IL-1β production upon poly(I:C) stimulation mapped to an uncharacterized non-coding RNA locus *LOC102723854* (Table 1; Figure 1A). The cQTL SNP rs10092649 on chromosome 8, which significantly correlated with IFN-γ production upon *S. pneumoniae* stimulation, mapped to the *TOX* (thymocyte selection-associated high-mobility group box) protein coding gene (Figure S5). Genetic polymorphisms in the *TOX* gene regulate T cell development and enhanced the differentiation of NK cells, which give rise to a population showing effector functions of mature NK cells.[54] Among females, the genome-wide significant SNP rs79030770 on chromosome 5, was associated with IL-10 production upon *C. burnetii* stimulation (Table 1; Figure 1B). SNP rs79030770 is near *EGFR* (epidermal growth factor receptor) gene, whose mutations are potential risk factors for lung adenocarcinoma, and more prevalent in females than in males.[55] These mutations are often linked to a better response to EGFR-targeted therapies, like tyrosine kinase inhibitors[56] (Figure S6). In addition, the SNP rs17170577 was associated with IFN-γ production upon *Mtb* stimulation mapped to the *CNTNAP2* gene, known to be associated with dyslexia risk in females but not in males.[57] Several suggestive independent associations ($p > 5 \times 10^{-8}$ to 1×10^{-6}) were also observed in both groups after adjusting for covariates (Table S1).

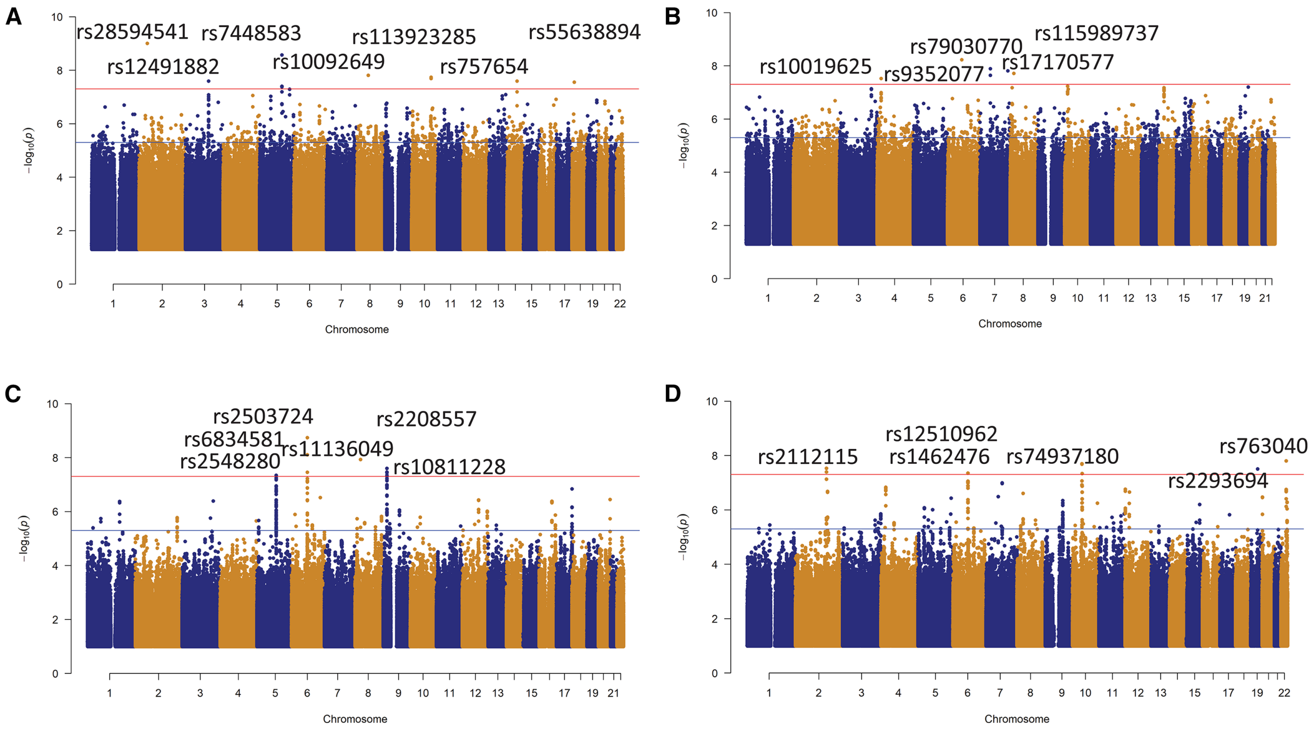

Figure 1. Manhattan plots of the cQTL variants in the Tanzanian and Dutch cohorts
The Manhattan plots show cQTLs that reached genome-wide significance in (A) male participants and (B) in the female participants upon stimulation of cytokines in the Tanzania cohort. The Manhattan plots show cQTLs that reached genome-wide significance in (C) male participants and (D) in the female participants upon stimulation of cytokines in the Dutch cohort. The red horizontal dashed line represents the genome-wide significant threshold ($p < 5 \times 10^{-8}$) and the blue dashed line denotes the suggestive evidence of association threshold. Nominally significant ($p < 5 \times 10^{-2}$) cQTLs are plotted.

Sex-stratified QTL mapping identifies 12 cQTLs in the Dutch cohort

Similarly, in the Dutch cohort, 12 independent cQTLs were identified as genome-wide significant (Table 2; Figures 1C and 1D). In males ($n = 173$), six lead SNPs (rs2548280, rs2503724, rs11136049, rs2208557, rs6834581, and rs10811228) reached genome-wide significance, while in females ($n = 235$), six lead SNPs (rs2112115, rs1462476, rs74937180, rs2293694, rs12510962, and rs763040) were significant. The top genome-wide significant SNP in males was rs2503724 on chromosome 6, associated with IL-1β production upon PHA stimulation (Table 2; Figure 1C). SNP rs2503724 is near the *BCKDHB* gene, which encodes the E1β subunit of the branched-chain α-keto acid dehydrogenase (*BCKDHB*) complex. The cQTL variants rs2548280 and rs11136049 identified in the males were found in the intronic region of the *ST8SIA4* and *FBXO16* genes, respectively; while rs2208557 and rs10811228 were both located in the SLC24A2 locus (Figure S7). *FBXO16* encodes a member of the F-box protein family, part of the ubiquitin protein ligase complex SCFs (SKP1-cullin-F-box).[58] *SLC24A2* (solute carrier family 24 member 2) is a member of the calcium/cation antiporter superfamily of transport proteins, expressed in brain and retina tissues, and is associated with visual transduction pathways and diseases such as epilepsy.

Among females, the top genome-wide significant SNP was rs74937180 on chromosome 10, associated with TNF-α production upon *C. albicans* conidia stimulation (Table 2; Figure 1D). SNP rs74937180 is near the *ANXA8* gene, which encodes a member of the annexin family, a group of evolutionary conserved Ca^{2+} and phospholipid-binding proteins. The encoded protein may function as an anticoagulant that indirectly inhibits the thromboplastin-specific complex. The variants rs2112115 and rs2293694 were located in the intronic regions of the *BAZ2B* and *DMKN* genes, while rs1462476 had no nearby genes (Figure S8). *BAZ2B*, bromodomain adjacent to zinc finger domain 2B, is a protein coding gene that has been suggested to play a potential role in transcription activation. The *DMKN* gene has been shown to be upregulated in inflammatory diseases.

Colocalization and interaction analyses in the Tanzanian cohort confirms 12 sex-specific cQTL loci

To identify genomic regions that are unique or shared between males and females, we performed genetic colocalization analysis for all the genome-wide significant loci. A tested region with posterior probability (PP4 > 0.75) shows a common association in both males and females. The posterior probability (PP1 > 0.75) indicates that only males have a genetic association in the testing region. We performed colocalization analysis on loci detected after cQTL mapping in the Tanzanian cohort. For males, we compared the seven significant loci with the same regions

Table 2. Genome-wide significant loci identified in the cQTL mapping among the Dutch

Cytokine	SNP	chr	*p* value m	β m	*p* value f	β f	Sex specific	Nearest gene	PP1	PP2	PP3	PP4
IL-6 *C. conidia*	rs10811228	9	2.5×10^{-8}	0.634	2.7×10^{-1}	0.12	male	*SLC24A2*	0.89	0.001	0.05	0.051
TNF-α *C. conidia*	rs11136049	8	1.1×10^{-8}	−0.859	8.5×10^{-1}	−0.026	male	*FBXO16*	0.87	0.002	0.05	0.024
TNF-α *C. conidia*	rs2208557	9	3.4×10^{-8}	0.72	3.1×10^{-1}	0.126	male	*SLC24A2*	0.9	0.0004	0.05	0.049
IL-1β PHA	rs2503724	6	1.8×10^{-9}	−0.657	7.7×10^{-1}	−0.028	male	*BCKDHB*	0.93	0.0001	0.04	0.049
IFN-γ *B. burgdorferi*	rs2548280	5	4.5×10^{-8}	0.48	7.5×10^{-1}	0.027	male	*ST8SIA4*	0.93	0.0001	0.04	0.049
TNF-α *C. burnetii*	rs6834581	4	1.0×10^{-08}	−0.699	1.5×10^{-05}	−0.398	shared	*TLR1-TLR6-TLR10*	0.00053	0.0014	0.528	0.657
TNF-α *C. burnetii*	rs12510962	4	1.4×10^{-02}	−0.332	1.3×10^{-08}	−0.555	shared	*TLR1-TLR6-TLR10*	0.00054	0.0014	0.524	0.474
IL-1β LPS	rs1462476	6	2.9×10^{-1}	0.128	4.4×10^{-8}	0.476	female	none	0.0001	0.903	0.06	0.069
IL-22 *S. aureus*	rs2112115	2	6.8×10^{-1}	−0.049	3.0×10^{-8}	0.573	female	*BAZ2B, WDSUB1*	0.001	0.89	0.05	0.05
IFN-γ *C. conidia*	rs2293694	19	6.2×10^{-1}	0.06	3.3×10^{-8}	−0.523	female	*DMKN, U2AF1L*	0.002	0.88	0.05	0.012
TNF-α *C. conidia*	rs74937180	10	5.5×10^{-1}	−0.091	1.4×10^{-8}	0.678	female	*ANXA8*	0.0008	0.893	0.03	0.0036
IL-6 *S. typhimurium*	rs763040	22	1.6×10^{-1}	0.256	1.6×10^{-8}	−0.807	female	*SHISAL1, RTL6*	0.002	0.668	0.22	0.143

p values for the lead SNP for both sexes are reported in the columns *p* value m and *p* value f.
PP1 indicates the posterior probability that only males have a genetic association in the testing region.
PP2 indicates the posterior probability that only females have a genetic association in the testing region.
PP3 indicates the posterior probability that both sexes have a genetic association in the testing region; however, the causal variants are different.
PP4 indicates the posterior probability that male and female share the same causal variant.

in females. Strong evidence for sex-specific causal effects was observed with PP1 values ranging from 0.84 to 0.92 in seven genomic loci: IL-1β poly(I:C) (rs28594541), IFN-γ *E. coli* (rs7448583), IFN-γ *S. pneumoniae* (rs10092649), IL- 6 *M. tuberculosis* (rs113923285), IL-10 *E. coli* (rs757654), IL-6 *M. tuberculosis* (rs12491882), and IL-1β poly(I:C) (rs55638894) (Figure 2; Table 1), suggesting no shared causal variants between sexes. For example, at the *TOX* locus (PP1 = 0.9), the variant rs10092649 showed strong association with production of IFN-γ upon *S. pneumoniae* stimulation ($p = 1.6 \times 10^{-8}$) in males but was not significant in females ($p = 8.6 \times 10^{-1}$). Further, IFN-γ in response to *S. pneumoniae* (rs10092649), the high PP1 (0.90) and very low PP2 (0.001) suggest strong evidence for a signal being present only in males. The low PP3 (0.05) and PP4 (0.032) further indicate a lack of support for a shared or distinct signal in both sexes. Therefore, this supports the interpretation that the genetic association is sex specific to males, rather than a shared signal (Table S2).

We next performed similar colocalization analysis in the five loci detected after cQTL mapping in females. The five significant loci were compared with the same regions in males. The posterior probability (PP2 > 0.75) indicates that only females have a genetic association in the testing region. Strong evidence for sex-specific effects was found, with PP2 values ranging from 0.81 to 0.89 in five genomic loci: IL-10 *S. typhimurium* (rs9352077), IL-10 *C. burnetii* (rs79030770), IFN-γ *M. tuberculosis* (rs17170577), IL-10 *S. pneumoniae* (rs115989737), and IL-10 *S. typhimurium* (rs10019625) (Figure 3; Table 1), again suggesting no shared causal variants between sexes. For example, at the EGFR locus (PP2 = 0.89), the SNP rs79030770 on chromosome 7, linked with IL-10 production upon C. *burnetii* stimulation, was significant in females ($p = 1.3 \times 10^{-8}$) but not in the males ($p = 7.9 \times 10^{-1}$). Furthermore, among the 12 loci we observed a statistically significant interaction effect between sex and the genotypes in all the sex-specific cQTLs (Figure S9), supporting the robustness of the findings.

Colocalization and interaction analysis confirms sex-specific effects of six cQTLs in the Dutch cohort

In the Dutch cohort, to test whether these cQTLs are shared or unique between the sexes, genetic colocalization analyses were performed for genome-wide significant loci identified in both males and females.

For males, the six significant loci were compared with the same regions in females. Strong evidence for sex-specific effects was observed with PP1 values ranging from 0.87 to 0.93 in five of the six genomic loci: IFN-γ *B. burgdorferi* (rs2548280), IL-1β PHA (rs2503724), TNF-α *C. conidia* (rs11136049 and rs2208557), and IL-6 *C. conidia* (rs4238282) (Figure 4; Table 2), suggesting no shared causal variants between sexes. For example, at the *ST8SIA4* locus (PP1 = 0.93), the variant rs2548280 showed strong association with production of IFN-γ upon *B. burgdorferi* stimulation ($p = 4.5 \times 10^{-8}$) in males but was not significant in females ($p = 7.5 \times 10^{-1}$). For the variant rs6834581, which showed strong association with production of TNF-α upon *C. burnetii* stimulation ($p = 1.0 \times 10^{-8}$), we did not observe any male-specific effects (PP1 = 0.0005) as affirmed by the strong colocalization with PP4 value of 0.657 (Table 2).

A similar colocalization analysis was next performed in the six significant loci detected after cQTL mapping

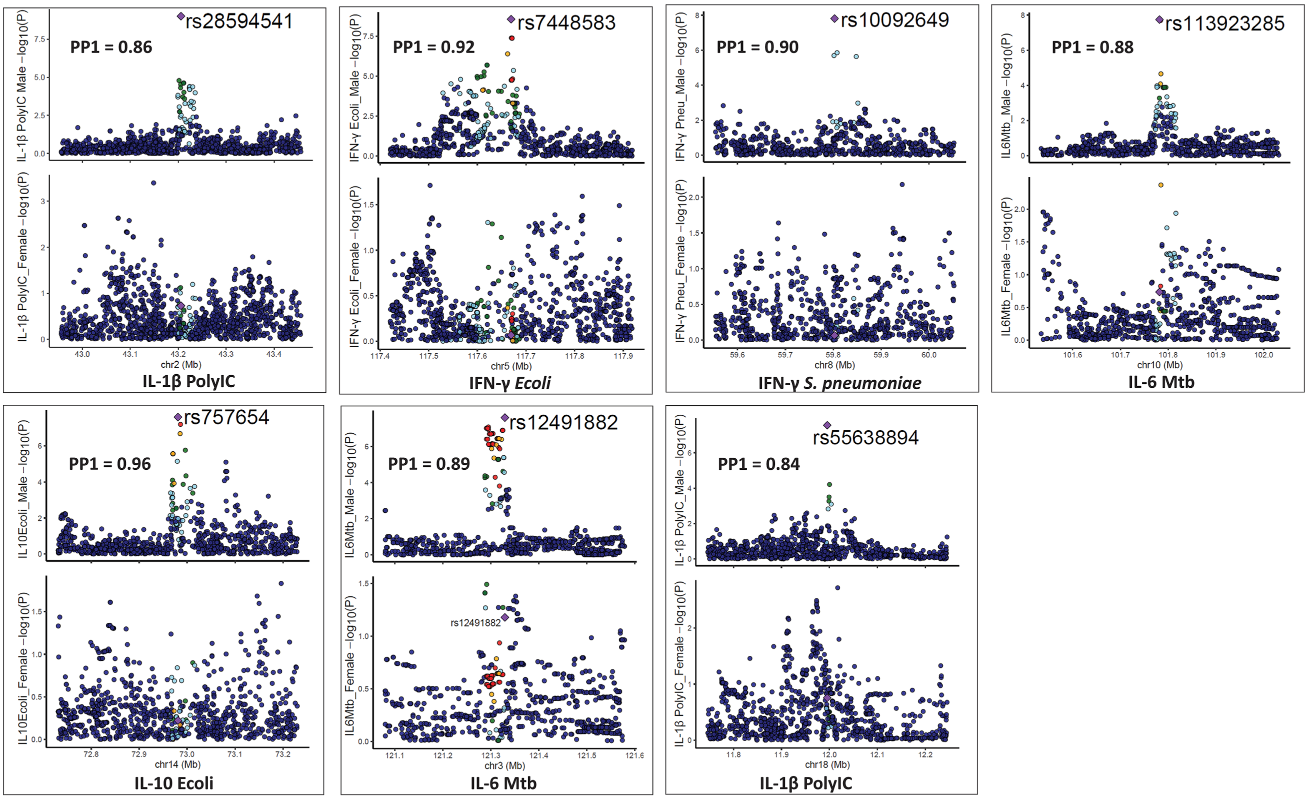

Figure 2. Illustration of colocalization analysis results among males in the Tanzanian cohort

Locus comparison plots of genomic loci between males and females. The cytokine-stimuli names and strength of association (–log10 *p* values) are displayed on the vertical axis against the chromosomal physical position on the horizontal axis. The posterior probability values (PP1) are also indicated on the plot. The top SNP in the locus is indicated by purple diamond, which is used to compute pairwise LD levels with other SNPs in the genomic region using the 1000 Genome African reference panel.

in females, in comparison with the same regions in males. Strong evidence for sex-specific effects was found, with PP2 values ranging from 0.67 to 0.90 in five of the six genomic loci: IL-22 *S. aureus* (rs2112115), IL-1β LPS (rs1462476), TNF-α *C. conidia* (rs74937180), IFN-γ *C. conidia* (rs2293694), and IL-6 *S. typhimurium* (rs763040) (Figure 5; Table 2), again suggesting no shared causal variants between sexes. For example, the SNP rs74937180 (PP2 = 0.89) on chromosome 10, linked with TNF-α production upon *C. conidia* stimulation was significant in females ($p = 1.4 \times 10^{-8}$) but not in the males ($p = 5.5 \times 10^{-1}$). Additionally, this variant yielded a high posterior probability for PP1 (0.89) but low PP4 (0.004), indicating a strong association signal exclusively in females. In contrast, the PP2 value was very low (0.001), suggesting the absence of a corresponding signal in males. Additionally, low values for PP3 (0.03) and PP4 (0.004) provided little evidence for either distinct or shared causal variants across sexes, supporting the conclusion that this association is female specific (Tables 2 and S3). Variant rs12510962 on chromosome 4, which showed strong association with production of TNF-α upon *C. burnetii* stimulation ($p = 1.3 \times 10^{-8}$), did not show any female-specific effects (PP2 = 0.0014) as affirmed by the strong colocalization with a PP4 value of 0.474 (Table 2).

In addition, among the 10 sex-specific loci identified, 6 showed a statistically significant interaction between sex and the genotypes of the sex-specific cQTLs (Figure S10). These included two male-specific loci (IFN-γ *B. burgdorferi* [rs2548280] and IL-1β PHA [rs2503724]) and four female-specific loci (IL-22 *S. aureus* [rs2112115], TNF-α *C. conidia* [rs74937180], IFN-γ *C. conidia* [rs2293694], and IL-6 *S. typhimurium* [rs763040]). Interaction analysis further revealed that three male-specific loci and one female-specific locus were not statistically significant, suggesting that a sex effect is present in at least one sex but with weak inter-sex effect size difference. Overall, applying three complementary statistical approaches including *p* value look-up, colocalization analysis, and interaction analysis enabled us to identify robust sex-specific signals.

Genome-wide significant cQTL associations with all cytokine measurements in the Tanzanian cohort

To assess whether the identified genome-wide significant cQTLs were also associated with other cytokine-stimulation pairs, cQTL variants were evaluated across all cytokine-stimulus pairs in both sexes.

In the Tanzanian cohort, the identified 12 genome-wide significant cQTLs are also sex specific (7 males and 5 females). In the analysis conducted in the males, apart from the 7 genome-wide significant cQTLs, we observe

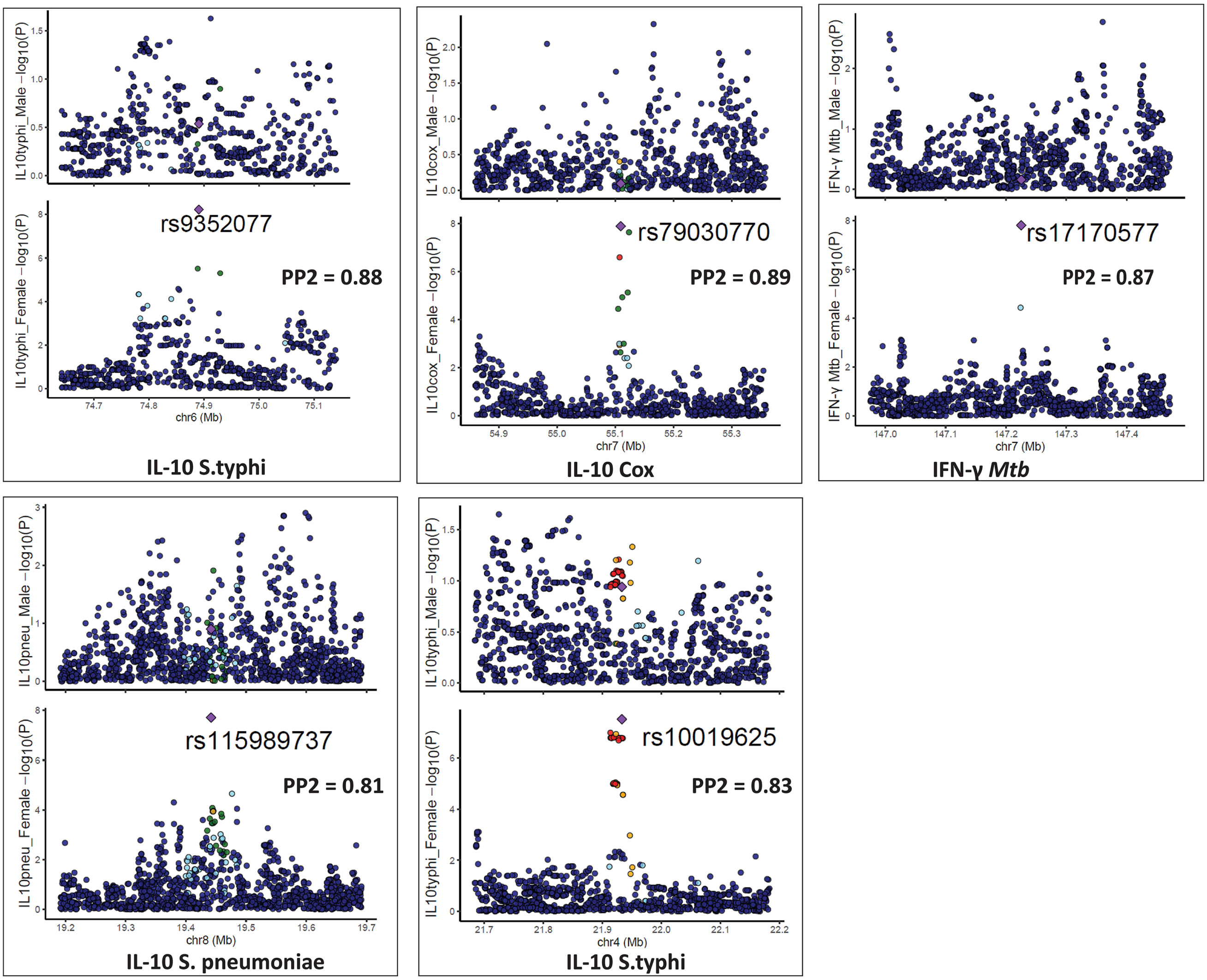

Figure 3. Illustration of colocalization analysis results among females in the Tanzanian cohort
Locus comparison plots of genomic loci between males and females. The cytokine-stimuli names and strength of association (–log10 p values) are displayed on the vertical axis against the chromosomal physical position on the horizontal axis. The posterior probability values (PP2) are also indicated on the plot. The top SNP in the locus is indicated by purple diamond, which is used to compute pairwise LD levels with other SNPs in the genomic region using the 1000 Genome African reference panel.

more other cytokine-stimulation pairs associated with the cQTL SNPs compared with the analysis conducted in the females (Figure 6A). For example, among males the genome-wide significant cQTL rs10092649 associated with IFN-γ upon *S. pneumoniae* stimulation also showed nominal association ($5 \times 10^{-2} < p < 5 \times 10^{-6}$) with 8 other cytokine-stimulation pairs, whereas in the females it was associated with only three cytokine-stimulation pairs.

Also, among the five genome-wide significant cQTLs identified in the females, we observe more cytokine-stimulation pairs associated with the female group contrary to the male group (Figure 6B). For example, the genome-wide significant cQTL rs9352077, associated with IL-10 after *S. typhimurium* stimulation, was also nominally associated with 13 other cytokine-stimulation pairs, whereas in the males it was associated with only 5 cytokine-stimulation pair.

Genome-wide significant cQTL associations with all cytokine measurements in the Dutch cohort

We evaluated whether the identified genome-wide significant cQTLs were also associated with other cytokine-stimulation pairs, across all cytokine-stimulus pairs in both males and females.

In the Dutch cohort, among the 12 identified genome-wide significant cQTLs, 6 are also sex specific (2 males and 4 females). Among the genome-wide significant loci in the male's analysis, we observe more significant cytokine-stimulation pairs associations with the males as compared with females (Figure 7A). For example, the genome-wide significant cQTL rs2503724 associated with IL-1β after PHA stimulation was also associated with five other cytokine-stimulation pairs, whereas in the females it was not significantly associated with any cytokine-stimulation pair. However, for the variant rs6834581 which was not sex specific, we

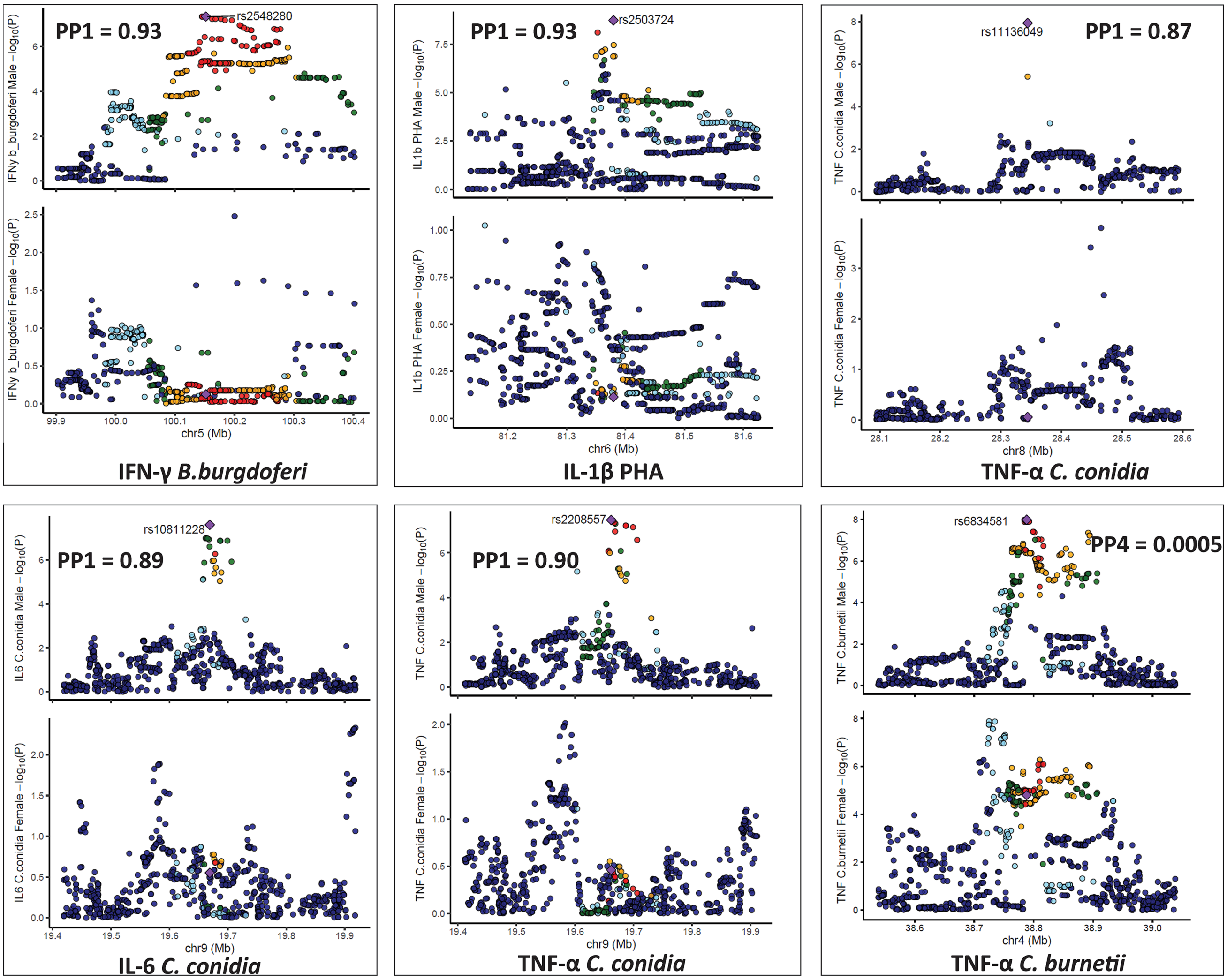

Figure 4. Locus comparison plots for each cQTL among males in the Dutch cohort
The plot represents the loci in both males and females. For each locus, the upper plot represents the males, the bottom plot represents the females. The cytokine-stimuli names and strength of association (–log10 *p* values) are displayed on the vertical axis against the chromosomal physical position on the horizontal axis. The posterior probability values (PP1) are also indicated on the plot. The top SNP in the locus is indicated by purple diamond, which is used to compute pairwise LD levels with other SNPs in the genomic region using the 1000 Genome European reference panel.

observe similar associations in both the males and females. For example, the cQTL rs6834581 associated with IL-1β after *C. burnetii* and poly(I:C) stimulation and IL-6 upon poly(I:C) stimulation, the variant is genome-wide significant in both males and females. Overall, where the cQTL is shared between males and females, we also observe many cytokine-stimulation pairs that are shared in both males and females. However, where the cQTLs act in a sex-specific manner, we find more associations of that cQTL in that specific sex compared with the other sex. For instance, the genome-wide significant cQTL rs2548280associated with IFN-γ upon *B. burgdorferi* stimulation is also nominally associated with four other cytokine-stimulation pairs in the males, but we did not observe any associations in the females (Figure 7A).

In the analysis of the females, we observe more significant cytokine-stimulation pair associations with the females compared with males (Figure 7B). For example, the genome-wide significant cQTL rs2112115 associated with IL-22 after *S. aureus* stimulation was also nominally associated with four other cytokine-stimulation pairs, while in the males it was significantly associated with one cytokine-stimulation pair. The effect of another cQTL variant rs74937180 correlating with three other cytokine-stimulation pairs in females was completely masked in the males.

Genome-wide significant cQTL associations with common cytokine measurements in the Tanzanian and Dutch cohorts

Further investigating the 12 cytokine-stimulation combinations common in both African and European datasets can reveal shared immune mechanisms across the populations, reducing potential bias or confounding. Among the

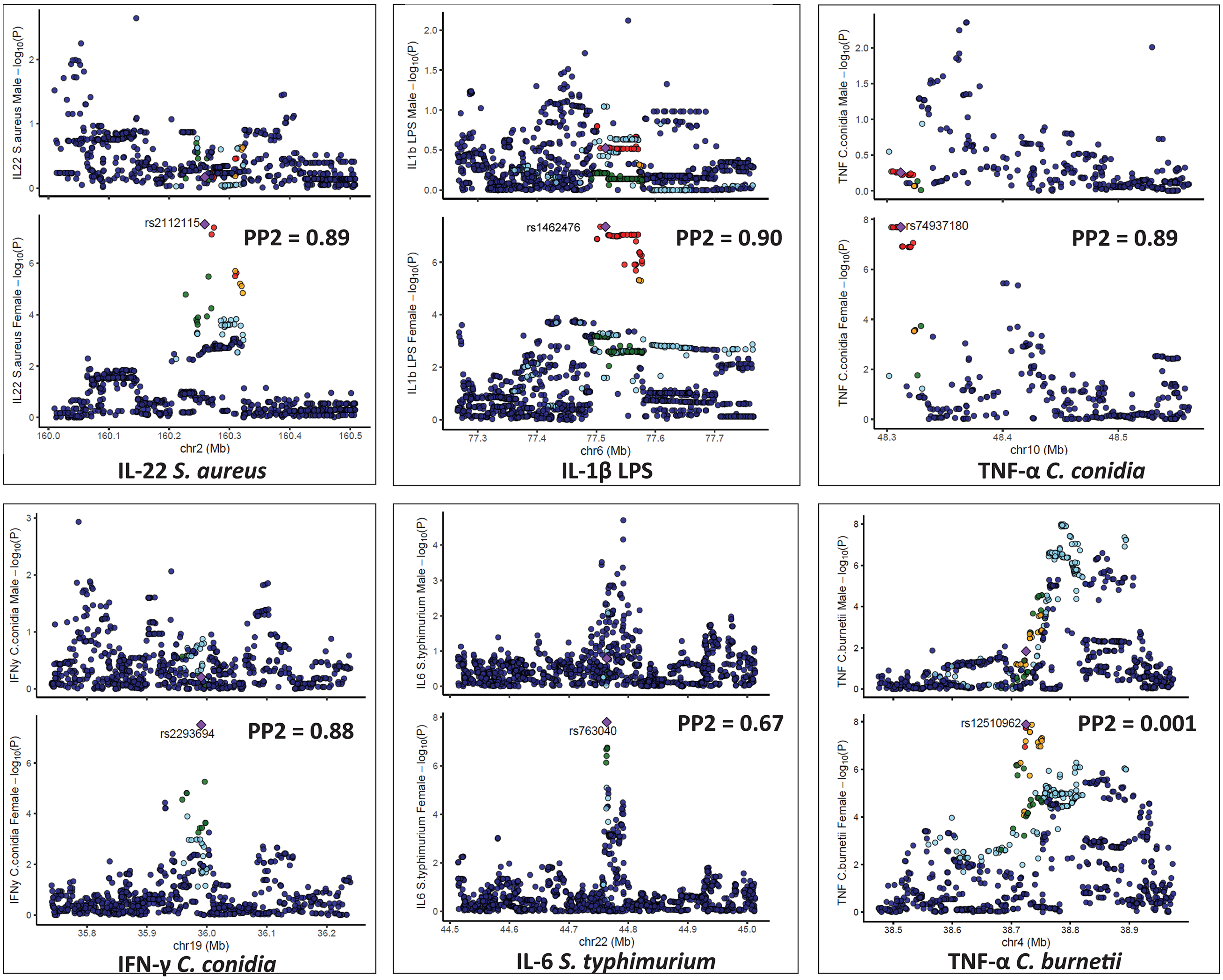

Figure 5. Locus comparison plots association plot for each cQTL among females in the Dutch cohort
The plot represents the loci in both males and females. For each locus, the upper plot represents the males, the bottom plot represents the females. The cytokine-stimuli names and strength of association (–log10 p values) are displayed on the vertical axis against the chromosomal physical position on the horizontal axis. The posterior probability values (PP2) are also indicated on the plot. The top SNP in the locus is indicated by purple diamond, which is used to compute pairwise LD levels with other SNPs in the genomic region using the 1000 Genome European reference panel.

genome-wide significant cQTLs, we observed more suggestive associations with other cytokine-stimulation pairs in the Tanzanian males in comparison with the Dutch males. However, more suggestive associations with other cytokine-stimulation pairs were observed in the Dutch females in comparison with the Tanzanian females (Figure S11). Overall, in the datasets we observed some of the cQTL loci were common to several stimuli, but many were stimulus specific. For example, the male genome-wide significant cQTL rs7448583 associated with IFN-γ upon *E. coli* stimulation, also showed association with IFN-γ, TNF-α, and IL6 upon LPS stimulations (Figure S11).

In the Tanzanian cohort, among the males seven genome-wide significant cQTLs we observe more other cytokine-stimulation pairs associated with the cQTL SNPs compared with the analysis conducted in the females (Figure S11A). For example, the genome-wide significant cQTL rs7448583 associated with IFN-γ upon *E. coli* stimulation also showed association with six other cytokine-stimulation pairs that are common between the cohorts; whereas, in the females, it was not associated with any cytokine-stimulation pair.

In the Dutch cohort, among the females five genome-wide significant cQTLs we observe more cytokine-stimulation pairs associated with the cQTL SNPs compared with the analysis conducted in the males (Figure S11B). For example, the effect of another cQTL variant rs74937180 correlating with eight other cytokine-stimulation pairs in females was completely masked in the males.

Findings from combined-sex cQTL mapping results across both cohorts

To provide a comprehensive overview of the genetic variations associated with cytokine responses and to evaluate

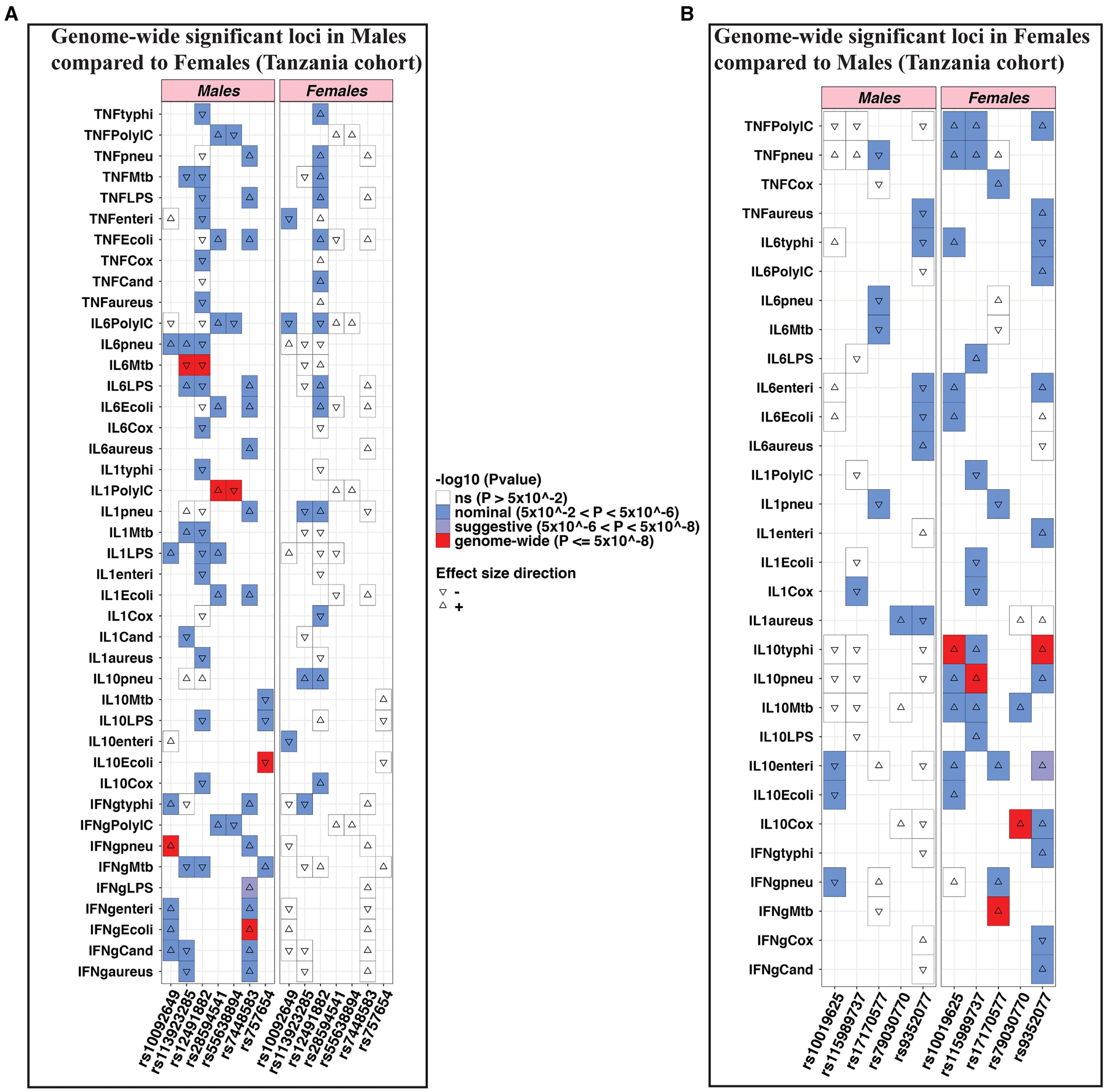

Figure 6. Heatmap showing the association of the genome-wide significant cQTLs with all the available cytokine measurements in the Tanzania cohort

The horizontal lines represent the ordered cytokine-stimulation pair. For each SNP, the –log10(p value) is shown both for males and for females. All the genome-wide significant SNPs shown were sex specific. Color key ranges are: not significant, white; nominal, blue; suggestive, gray; and all the genome-wide significant associations are shown in red. The heatmaps display only cytokine-stimulation pairs that showed at least one significant SNP association. For visualization purposes, cytokine-stimulation pairs that have no association with any of the genome-wide significant SNPs are not shown on the y axis. For cytokines without a box, these include cytokine-stimuli pair that were excluded for not showing any significant associations ($p > 0.05$) in both males and females.

whether the loci identified in the sex-stratified analyses remained insignificant when the data were pooled, we conducted genome-wide cQTL mapping in the combined sample of males and females (Figure S12).

Among the Tanzanians, four loci surpassed the genome-wide significant threshold (Figure S12A), while three genome-wide significant hits were observed in the Dutch cohort (Figure S12B). Additionally, we observed that the SNPs identified as significant in sex-stratified analyses did not reach genome-wide significance when analyzed collectively. For example, in the Tanzanian cohort the variant rs10092649, associated with IFN-γ response upon *S. pneumoniae* stimulation, showed a highly significant association in males ($p = 1.6 \times 10^{-8}$), but not in females ($p = 0.859$), nor in the joint analysis ($p = 0.0002$), suggesting a male-specific effect. Also, the SNP rs9352077 was

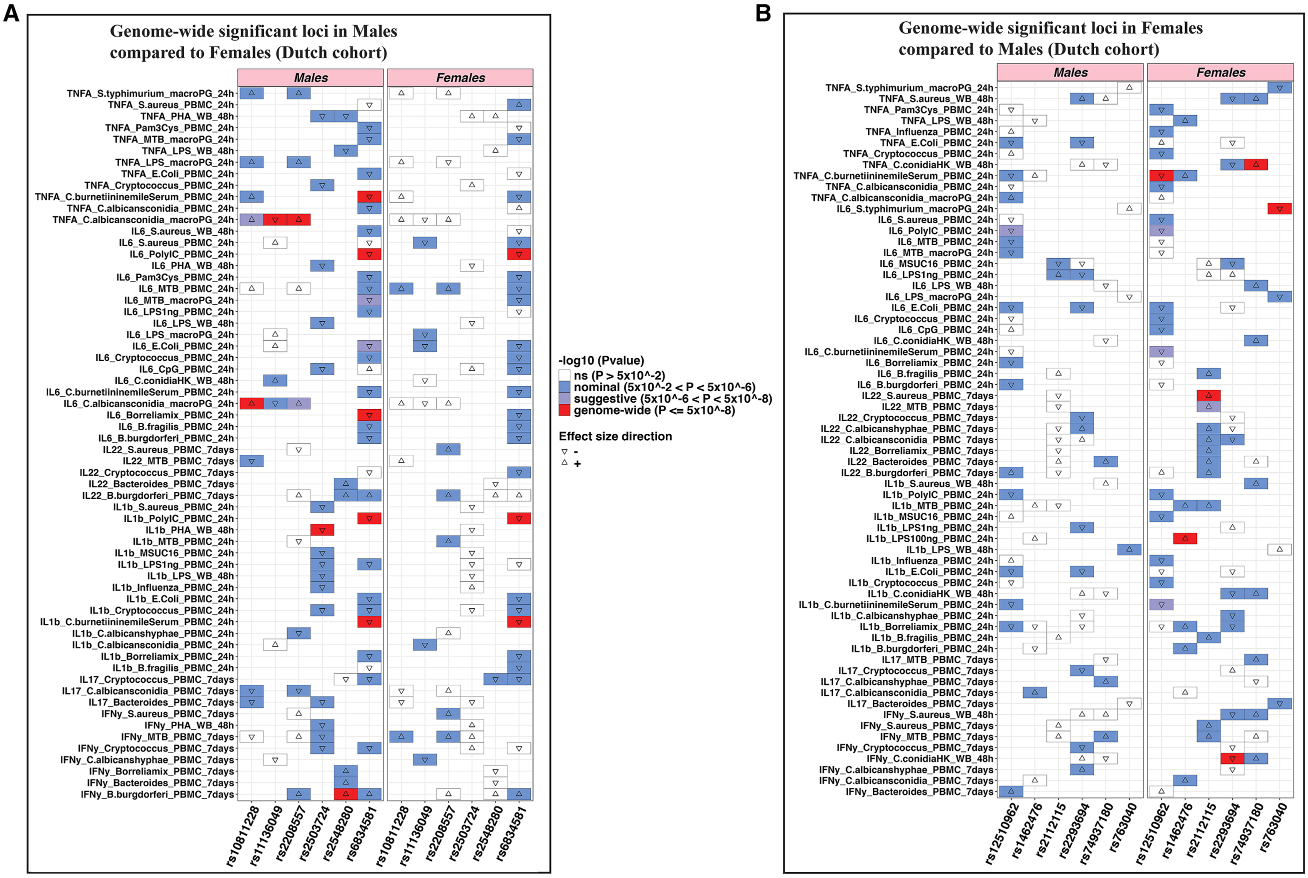

Figure 7. Heatmap showing the association of the genome-wide significant cQTLs with all the available cytokine measurements in the Dutch cohort

The horizontal lines represent the ordered cytokine-stimulation pair. For each SNP, the –log10(p value) is shown both for males and for females. All the genome-wide significant SNPs shown were sex specific except SNP (rs6834581 and rs12510962). Color key ranges are: not significant, white; nominal, blue; suggestive, gray; and all the genome-wide significant associations are shown in red. The heatmaps display only cytokine-stimulation pairs that showed at least one significant SNP association. For visualization purposes, cytokine-stimulation pairs that have no association with any of the genome-wide significant SNPs are not shown on the y axis. For cytokines without a box, these include cytokine-stimuli pair that were excluded for not showing any significant associations ($p > 0.05$) in both males and females.

significantly associated with IL-10 production upon *S. typhimurium* stimulation in females ($p = 5.9 \times 10^{-9}$), but not in males ($p = 0.289$) or in the joint analysis ($p = 0.00004$), highlighting a female-specific genetic effect (Table S4).

Several loci showed evidence of sex-specific cQTL effects in the Dutch cohort. The SNP rs2503724 was significantly associated with IL-1β production in response to PHA stimulation in males ($p = 1.8 \times 10^{-9}$), but showed no association in females ($p = 0.77$) or in the joint analysis ($p = 0.766$), indicating a male-specific effect. Conversely, rs74937180 was significantly associated with TNF-α production in response to *Candida* conidia stimulation in females ($p = 1.4 \times 10^{-8}$), but not in males ($p = 0.55$) or the joint analysis ($p = 0.454$), supporting a female-specific effect (Table S5).

This suggests that these associations may be sex specific or modulated by sex-related factors on cytokine responses. The joint analysis, which included the covariates as in the stratified models (in addition to sex), provided a baseline reference and confirmed that the observed sex-specific cQTLs were not driven by shared effects across sexes. These results emphasize the importance of conducting stratified analyses to uncover sex-differentiated genetic regulation of cytokine responses (Tables S4 and S5; Figure S12).

Phenotypes associated with the identified genome-wide cQTLs in males

PhenoScanner was utilized to investigate other previously identified phenotypes associated with the identified genome-wide cQTLs. We cross-referenced all the genome-wide significant cQTL variants in the males with other phenotypic traits using a threshold of 1×10^{-5} to explore any potential biological pleiotropy. In the Tanzanian cohort, the cQTL variant rs55638894 showed significant association with neuralgia, neuritis, and radiculitis NOS. Whereas, in the Dutch cohort, the shared cQTL variant rs6834581 was significantly associated with traits related to the immune response, such as allergic diseases, asthma, and lymphocyte counts (Table 3).

Table 3. **Significant associations to traits and diseases of the cQTLs lead variants in the male**

snp	Cohort	Trait	p value
rs6834581	Dutch	lymphocyte count	2.3×10^{-11}
rs6834581	Dutch	allergic disease	6.1×10^{-37}
rs6834581	Dutch	asthma	3.6×10^{-14}
rs6834581	Dutch	doctor diagnosed hay fever or allergic rhinitis	1.5×10^{-21}
rs6834581	Dutch	hay fever, allergic rhinitis or eczema	1.6×10^{-72}
rs6834581	Dutch	home area population density: Scotland large urban area	1.3×10^{-10}
rs6834581	Dutch	no blood clot, bronchitis, emphysema, asthma, rhinitis, eczema or allergy diagnosed by doctor	6.8×10^{-60}
rs6834581	Dutch	self-reported asthma	1.1×10^{-13}
rs6834581	Dutch	self-reported hay fever or allergic rhinitis	2.5×10^{-16}
rs55638894	Tanzanian	neuralgia, neuritis, and radiculitis NOS	7.9×10^{-7}
rs55638894	Tanzanian	cataract	8.6×10^{-5}
rs55638894	Tanzanian	benign neoplasm of ovary	4.1×10^{-4}
rs55638894	Tanzanian	cancer of larynx, pharynx, nasal cavities	6.0×10^{-4}
rs55638894	Tanzanian	cancer of nasal cavities	8.2×10^{-4}
rs28594541	Tanzanian	loose body in joint	4.8×10^{-4}
rs28594541	Tanzanian	cholecystitis without cholelithiasis	6.2×10^{-4}
rs10092649	Tanzanian	skin cancer	5.2×10^{-4}
rs10092649	Tanzanian	other non-epithelial cancer of skin	5.7×10^{-4}
rs10092649	Tanzanian	diabetes mellitus	9.4×10^{-4}
rs7448583	Tanzanian	hydrocele	6.8×10^{-4}
rs12491882	Tanzanian	cardiac conduction disorders	7.4×10^{-4}

We further queried associations of the other traits with the genome-wide cQTLs at a relaxed p value threshold to 0.001. This followed after an initial threshold of 1×10^{-5}, which had only identified associations with rs6834581 and rs55638894.

In the Dutch cohort, additional associations were identified for rs2548280, rs10811228, rs11136049, and rs2208557. The male-specific variant rs2548280 was associated with various traits related to body measurements (e.g., arm fat-free mass, arm predicted mass, height, leg fat-free mass, leg predicted mass), with the strongest association observed with white blood cell count ($p = 2.5 \times 10^{-4}$). Another male-specific SNP rs11136049 was associated with different diseases, such as diffuse non-Hodgkin lymphoma, and reported occurrences of cancer. The male-specific cQTL rs2208557 was associated with essential hypertension, symptoms and signs involving the urinary system and creatine levels. Traits and diseases such as body measurements and other prostate disorders were associated with male-specific SNP rs2503724, while the only association for male-specific cQTL rs10811228 was with varicose veins of lower extremities ($p = 6.6 \times 10^{-4}$).

In the Tanzanian cohort with the relaxed p value, associations were identified for rs55638894, rs28594541, and rs10092649. The male-specific SNP rs55638894 was associated with cataract ($p = 8.6 \times 10^{-5}$) and larynx cancer diseases ($p = 6.0 \times 10^{-4}$), with the strongest association observed with neuralgia, neuritis, and radiculitis NOS ($p = 7.9 \times 10^{-7}$). Joint pain was associated with SNP rs28594541 ($p = 4.8 \times 10^{-4}$); while the male-specific cQTL variant rs10092649 was associated with skin cancer ($p = 5.2 \times 10^{-4}$) and diabetes mellitus ($p = 9.4 \times 10^{-4}$) (Table 3).

Phenotypes associated with the identified genome-wide cQTLs in females

We cross-referenced all the genome-wide significant cQTL variants in the females with other phenotypic traits, using a threshold of 1×10^{-5} to explore any previously identified potential biological mechanisms. In the Tanzanian cohort, none of the cQTLs variants showed association with any disease or traits at this threshold. In the Dutch cohort the shared cQTL variant rs12510962 at chromosome 4 and the TLR1-TLR6-TLR10 locus was identified to be associated with allergic diseases ($p = 1.7 \times 10^{-10}$), *Helicobacter pylori*

Table 4. **Significant associations to traits and diseases of the cQTLs lead variants in the females**

snp	Cohort	Trait	*p* value
rs12510962	Dutch	lymphocyte count	3.7×10^{-20}
rs12510962	Dutch	lymphocyte percentage of white cells	1.0×10^{-9}
rs12510962	Dutch	allergic disease	1.8×10^{-10}
rs12510962	Dutch	*Helicobacter pylori* seroprevalence	1.0×10^{-10}
rs12510962	Dutch	hay fever, allergic rhinitis or eczema	1.5×10^{-19}
rs12510962	Dutch	no blood clot, bronchitis, emphysema, asthma, rhinitis, eczema, or allergy diagnosed by doctor	5.9×10^{-16}
rs115989737	Tanzanian	renal colic	4.2×10^{-4}
rs17170577	Tanzanian	other specified gastritis	6.9×10^{-4}
rs17170577	Tanzanian	hemorrhage during pregnancy; childbirth and postpartum	8.7×10^{-4}
rs79030770	Tanzanian	infertility, female	7.8×10^{-4}
rs79030770	Tanzanian	myoclonus	8.1×10^{-4}
rs9352077	Tanzanian	inflammatory and toxic neuropathy	8.4×10^{-4}
rs9352077	Tanzanian	influenza	9.1×10^{-4}

sero prevalence ($p = 1.0 \times 10^{-10}$), and lymphocyte count ($p = 3.7 \times 10^{-20}$) (Table 4).

In the Dutch cohort, by relaxing the *p* value threshold to 0.001, associations were found for all female-specific variants. The female-specific SNP rs74937180 was associated with infections treated with ciprofloxacin treatment ($p = 3.92 \times 10^{-4}$), while female-specific variant rs763040 was linked to conditions associated with use of cymalon cranberry, such as urinary tract infections[59,60] and the antihypertensive combination treatment lisinopril/hydrochlorothiazide.[61] Traits associated with female-specific cQTL rs2293694 included elevated blood glucose levels among others.[62] Female-specific variant rs2112115 was associated with granulocyte, myeloid, and white blood cell counts. The female-specific SNP rs12510962 showed several associations, including allergic diseases, various cell counts, and asthma.[63]

In the Tanzanian cohort with the relaxed *p* value, associations were identified for rs115989737, rs17170577, rs79030770, and rs9352077 female-specific variants. The female-specific SNP rs115989737 was associated with renal colic pain ($p = 4.2 \times 10^{-4}$), while variant rs17170577 was linked to gastritis ($p = 6.9 \times 10^{-4}$) and hemorrhage during pregnancy; childbirth, and postpartum ($p = 8.7 \times 10^{-4}$). The variant rs79030770 was associated with female infertility ($p = 7.8 \times 10^{-4}$) and myoclonus ($p = 8.1 \times 10^{-4}$); while rs9352077 was associated with inflammatory and toxic neuropathy ($p = 8.4 \times 10^{-4}$) and influenza ($p = 9.1 \times 10^{-4}$) (Table 4).

Functional enrichment analysis of cQTLs identified in the Tanzanian and Dutch cohorts

To determine the biological relevance of the cytokine production upon various stimuli with cQTLs, we queried two of the most comprehensive biological annotation and pathway databases: Kyoto Encyclopedia of Genes and Genomes (KEGG)[64,65] and Reactome.[66,67] We performed gene set enrichment functional analysis for pathways in the cytokines using the Web Gestalt (WEB-based Gene Set Analysis Toolkit) web tool platform. To identify these pathways, we first prioritized suggestive independent cQTLs ($p < 1 \times 10^{-5}$) for each of the sex-stratified cohorts and extracted gene sets mapping near these genetic variants by using a window size of 250 kb upstream and downstream of each SNP. This analysis revealed significant enrichment of different pathways (FDR $p < 0.05$).

In the Tanzanian cohort, the top pathways included "Growth hormone synthesis, secretion and action," and "human cytomegalovirus infection" for the males (Figure S13A), and "type II diabetes mellitus" and "gastric cancer" disease pathways for the females (Figure S13B). In the Dutch cohort, we observe less significant pathways related to immune function and disease pathways (Figures S14A and S14B), including "Toll-like receptor signaling pathway," "human immunodeficiency virus 1 infection," "diseases of the immune system," and "platelet activation" for the genes associated with cQTLs in the males (Figure S14A). This indicates that the *cis*-genes surrounding the cQTLs primarily regulate pathogen recognition receptors such as TLR-mediated signaling. Moreover, among the females we also observed less significantly enriched pathways related to immune and diseases for the genes associated with the cQTLs (Figure S14B). For example, the top-ranked KEGG pathways were mostly related to diseases such as asthma, "type 1 diabetes mellitus," and "Yersinia infection." Some genetic variants are known to affect gene expression patterns, which may influence the intensity of the immune response and increase

the risk of diabetes and their complications.[68] While the top-ranked Reactome pathways were mostly related to immune function, including "diseases of the immune system," and "diseases associated with the TLR signaling cascade." TLRs are pathogen recognition receptors through which our innate immune system recognizes foreign antigens and activates host protective inflammatory responses.[69] The identification of sex-specific biological pathways is vital for the development of effective therapeutics, particularly for diseases that exhibit differential expression between sexes.

Discussion

This study provides a comprehensive analysis of sex-specific genetic effects on cytokine responses in humans, using two independent cohorts from Tanzania and the Netherlands. While previous research has explored the influence of sex on the immune responses,[70,71] most studies relied on animal models or sex-agnostic approaches that may obscure sex-dependent effects. By performing sex-stratified cQTL mapping, supported by colocalization and interaction analyses, we identified sex-specific loci that regulate cytokine production, together with shared loci that act independently of sex. These findings highlight both common and divergent mechanisms underlying immune regulation in men and women.

In the Tanzanian cohort, the most notable male-specific association was rs10092649 at the *TOX* locus, linked to IFN-γ production following S. *pneumoniae* stimulation. *TOX* is a transcription factor critical for T cell development and NK cell differentiation, with roles in establishing long-lived effector populations.[54] The male-specific regulation of IFN-γ responses suggests that *TOX* variants may preferentially shape protective cellular immunity in men, consistent with observed differences in infection outcomes. In the Dutch cohort, two additional male-specific loci were identified: rs2548280 within *ST8SIA4*, a sialyltransferase involved in receptor glycosylation and immune signaling,[72] and rs11136049 within *FBXO16*, an F-box protein regulating ubiquitin-mediated protein degradation.[73] These findings suggest that male-biased immune regulation involves enhanced modulation of receptor signaling and effector cell differentiation, potentially contributing to sex differences in pathogen clearance and inflammation.

In females, several loci were uniquely associated with cytokine regulation. In the Tanzanian cohort, rs79030770 near *EGFR* was associated with IL-10 production in response to Cox stimulation. *EGFR* signaling is known to influence immune modulation as well as oncogenic pathways, with mutations more prevalent in women with lung adenocarcinoma.[55] The coupling of EGFR variants to IL-10, a key anti-inflammatory cytokine, suggests that female-specific EGFR regulation may promote stronger feedback control of inflammation, with potential implications for cancer susceptibility and treatment responses. Another Tanzanian female-specific locus, rs17170577 near *CNTNAP2*, was linked to IFN-γ production after M. *tuberculosis* stimulation. *CNTNAP2*, best known for its role in neurodevelopmental traits with female bias,[57] may represent an unexpected neuro-immune regulator of pathogen-induced cytokine responses in women.

In the Dutch cohort, rs74937180 near *ANXA8* was associated with TNF-α production following C. *albicans* stimulation. *ANXA8* belongs to the annexin family of Ca^{2+}- and phospholipid-binding proteins, with roles in coagulation and inflammatory processes.[74] Its female-specific regulation of TNF-α suggests a mechanism by which women may exhibit distinct inflammatory and thrombotic responses to infection.[75] Together, these findings underscore that female-biased regulation predominantly involves cytokine modulation through pathways intersecting with growth factor signaling, neuro-immune crosstalk, and coagulation biology.

Further analysis of the identified SNPs revealed that not all loci showed sex specificity. For example, cQTL variant rs12510962 at chromosome 4 near the TLR1-TLR6-TLR10 locus was strongly associated with cytokine production in both sexes in the Dutch cohort, with links to asthma, allergy, and immune-related traits.[76,77] This highlights a set of conserved innate immune regulators that function similarly across sexes. Importantly, however, our sex-stratified analysis revealed that several other loci, which were robustly associated in one sex, failed to reach genome-wide significance in pooled analyses. This finding demonstrates that sex-agnostic approaches can mask true genetic effects, underestimating the contribution of sex-specific regulatory variants.

The sex-specific mechanisms identified here provide plausible explanations for observed differences in disease susceptibility and outcomes. Male-biased regulation of IFN-γ responses through *TOX* and glycosylation/ubiquitin pathways may contribute to stronger cellular immunity against certain pathogens, consistent with epidemiological data showing lower male susceptibility to infections such as hepatitis viruses.[2,3] Conversely, female-biased IL-10 regulation via *EGFR* and TNF-α modulation through *ANXA8* may predispose women to heightened inflammatory or autoimmune responses, but also support better control of tissue damage.[10,78] The identification of *CNTNAP2* as a regulator of IFN-γ responses further suggests that neuro-immune interactions may contribute to sex differences in both infection and autoimmunity.

This study has limitations. First, sex chromosomes were excluded from the analysis, despite their established roles in immune regulation, particularly for genes escaping X-inactivation.[79] Future studies should include sex chromosome to better understand sex-specific genetic variations in the immune responses. Second, the sample sizes were reduced by sex-stratification, limiting power to detect additional cQTLs. Larger cohorts or meta-analyses

with sex-stratified approaches are necessary to identify more sex-specific genetic variants. Although MR-MEGA offers a robust framework for modeling ancestry-related effect heterogeneity in *trans*-ancestry meta-analyses, its application was not feasible in the present study due to the inclusion of only two population groups; incorporation of additional ancestrally distinct populations in future analyses would enable the use of MR-MEGA as a valuable extension of this work. Future studies with detailed reproductive and hormonal data would be well positioned to explicitly investigate menopause-related modulation of immune responses. Although individual-level data on menopausal status is unavailable in this cohort, population-based studies have shown that chronological age explains a greater proportion of variance in many inflammatory and cytokine markers than menopausal status alone.[80] We did not model interactions between autosomes and the X chromosome, despite recent work by Cheng et al., showing that sex-biased gene expression can arise from complex regulatory architectures involving autosome-X chromosome interactions and *trans*-eQTLs.[81] In contrast, our analyses focused on autosomal cQTLs and SNP × sex interaction models, which capture sex-dependent effect sizes. As a result, more distal or *trans*-acting mechanisms contributing to sex differences in cytokine responses may not be fully captured. Future studies integrating X chromosome variation, autosome-X interaction models, and *trans*-eQTL analyses will be important to provide a more comprehensive understanding of sex-biased immunogenetic regulation.[81,82] Additionally, replication in larger, independent cohorts is needed, ideally with harmonized cytokine stimulations. Furthermore, functional validation of the implicated loci is necessary to establish causal mechanisms.

Perspectives and significance

Our study identifies sex-specific genetic variants linked to cytokine production across diverse pathogens, underscoring the complexity of immune responses and highlighting the importance of considering sex differences in immunological research. The presence of both sex-specific and universal genetic influences suggests that, while certain variants distinctly modulate cytokine levels in males and females, others may play broader roles in immune function. These results have significant implications for personalized medicine, as they could inform targeted therapeutic approaches that account for genetic and sex-related factors in immune response. Future research could build on these findings by exploring how these genetic variations influence susceptibility to infections or autoimmune diseases, ultimately leading to more effective, tailored interventions for both sexes.

In conclusion, our study identifies distinct sex-specific genetic mechanisms regulating cytokine responses, including *TOX*-mediated IFN-γ regulation in males, *EGFR*-driven IL-10 modulation in females, *CNTNAP2*-linked neuro-immune control, and *ANXA8*-associated TNF-α production in women. These findings highlight how genetic regulation of immune pathways diverges between sexes, and suggest that incorporating sex-stratified analyses in immunogenetic studies can uncover mechanisms masked in pooled datasets. Ultimately, understanding these pathways may inform the development of personalized approaches to infectious and immune-mediated diseases that account for sex differences.

Data and code availability

- The cQTL association full summary statistics have been deposited to the Zenodo website under the accession numbers: DOI https://doi.org/10.5281/zenodo.18441954 for the male and female cohorts in the Tanzanian cohort.
- The Dutch cQTL association full summary statistics have been deposited to the Zenodo website under the accession numbers: DOI https://doi.org/10.5281/zenodo.18413538 and DOI https://doi.org/10.5281/zenodo.18379771 for the male and female cohorts, respectively, in the cohort.
- Anonymized metadata of the participants are available in an open access registry (DANS registry: https://doi.org/10.17026/dans-xgx-zuht).
- In order not to compromise research volunteers' privacy, individual-level 500FG and 300FG genetic data can be acquired by researchers upon successful application using the data request from for the 500FG cohort (http://www.humanfunctionalgenomics.org/site/?page_id=16).
- The form will be reviewed by the Data Access Committee, who will grant access upon approval.
- The phenotypic datasets accompanying this manuscript are available on Figshare (https://doi.org/10.5061/dryad.k3j9kd5b6).
- No custom code was generated and all software that is central to the research findings have been previously reported and are referenced throughout the paper mostly in the subjects and methods and web resources sections or as part of the figure legends.
- All other data are available in the main text and supplemental information.
- Any additional information required to reanalyze the data reported in this paper is available from the lead contact upon request.

Acknowledgments

The authors are grateful to all volunteers from the Tanzanian and Dutch cohorts without whom this research would not have been possible. We would like to thank J. Njau, J. Kwayu, and the late E. Kimaro for assistance with sample collection. This study was funded by the following grants: the European Union's Horizon 2020 Research and Innovation Program under the ERA-Net Cofund action no. 727565, the Joint Programming Initiative, A Healthy Diet for a Healthy Life (JPI-HDHL) (project 529051018) awarded to M.G.N. and Q.d.M., ZonMw (the Netherlands Organization for Health Research and Development) awarded to M.G.N. and Q.d.M., and EDCTP-EACCR (CSA2020NoE-3102 - EACCR3) awarded to C.A., Radboud Revolving Research Funds (3R-Fund) awarded to G.S.T., Spinoza Prize (NOW SPI94-212) and ERC

Advanced grant (no. 833247) awarded to M.G.N., and Hypatia tenure track grant from Radboudumc to V.I.K.

Author contributions

M.G.N., L.A.B.J., Q.d.M., V.K., B.T.M., and R.K. contributed to the conceptualization, study design, data interpretation, and led the project. G.S.T. and V.I.K. contributed to participants recruitment, data collection, and laboratory analyses. C.A. and C.K.B. performed statistical analysis and graphics of the data. C.A. wrote the original draft of the manuscript. C.K.B., G.S.T., R.C., I.R.P., N.K., V.I.K., B.T.M., R.K., L.A.B.J., M.G.N., Q.d.M., and V.K. contributed in writing, review, and editing the manuscript.

Declaration of interests

The authors declare no competing interests.

Supplemental information

Supplemental information can be found online at https://doi.org/10.1016/j.xhgg.2026.100593.

Web resources

Colocalization package, https://chr1swallace.github.io/coloc/articles/a01_intro.html

Human Functional Genomics Project, http://www.human.functionalgenomics.org/site/

LocusZoom, http://locuszoom.orgWeb Gestalt (WEB-based Gene Set Analysis Toolkit), https://www.webgestalt.org/

PhenoScanner, https://github.com/phenoscanner/phenoscanner

PLINK, https://github.com/phenoscanner/phenoscanner

Received: August 29, 2025
Accepted: March 16, 2026

References

1. Jacobsen, H., and Klein, S.L. (2021). Sex Differences in Immunity to Viral Infections. Front. Immunol. *12*, 720952. https://doi.org/10.3389/fimmu.2021.720952.
2. Guha-Sapir, D., and Schimmer, B. (2005). Dengue fever: new paradigms for a changing epidemiology. Emerg. Themes Epidemiol. *2*, 1. https://doi.org/10.1186/1742-7622-2-1.
3. Tsay, P.-K., Tai, D.-I., Chen, Y.-M., Yu, C.-P., Wan, S.-Y., Shen, Y.-J., and Lin, D.-Y. (2009). Impact of gender, viral transmission and aging in the prevalence of hepatitis B surface antigen. Chang Gung Med. J. *32*, 155–164.
4. Takahashi, T., Ellingson, M.K., Wong, P., Israelow, B., Lucas, C., Klein, J., Silva, J., Mao, T., Oh, J.E., Tokuyama, M., et al. (2020). Sex differences in immune responses that underlie COVID-19 disease outcomes. Nature *588*, 315–320. https://doi.org/10.1038/s41586-020-2700-3.
5. Klein, S.L., Hodgson, A., and Robinson, D.P. (2012). Mechanisms of sex disparities in influenza pathogenesis. J. Leukoc. Biol. *92*, 67–73. https://doi.org/10.1189/jlb.0811427.
6. Fairweather, D., and Rose, N.R. (2004). Women and Autoimmune Diseases. Emerg. Infect. Dis. *10*, 2005–2011. https://doi.org/10.3201/eid1011.040367.
7. Cook, M.B., McGlynn, K.A., Devesa, S.S., Freedman, N.D., and Anderson, W.F. (2011). Sex Disparities in Cancer Mortality and Survival. Cancer Epidemiol. Biomarkers Prev. *20*, 1629–1637. https://doi.org/10.1158/1055-9965.EPI-11-0246.
8. Ober, C., Loisel, D.A., and Gilad, Y. (2008). Sex-specific genetic architecture of human disease. Nat. Rev. Genet. *9*, 911–922. https://doi.org/10.1038/nrg2415.
9. Weiss, L.A., Pan, L., Abney, M., and Ober, C. (2006). The sex-specific genetic architecture of quantitative traits in humans. Nat. Genet. *38*, 218–222. https://doi.org/10.1038/ng1726.
10. Mayne, B.T., Bianco-Miotto, T., Buckberry, S., Breen, J., Clifton, V., Shoubridge, C., and Roberts, C.T. (2016). Large Scale Gene Expression Meta-Analysis Reveals Tissue-Specific, Sex-Biased Gene Expression in Humans. Front. Genet. *7*, 183.
11. Jansen, R., Batista, S., Brooks, A.I., Tischfield, J.A., Willemsen, G., van Grootheest, G., Hottenga, J.-J., Milaneschi, Y., Mbarek, H., Madar, V., et al. (2014). Sex differences in the human peripheral blood transcriptome. BMC Genom. *15*, 33. https://doi.org/10.1186/1471-2164-15-33.
12. Trabzuni, D., Ramasamy, A., Imran, S., Walker, R., Smith, C., Weale, M.E., Hardy, J., Ryten, M.; and North American Brain Expression Consortium (2013). Widespread sex differences in gene expression and splicing in the adult human brain. Nat. Commun. *4*, 2771. https://doi.org/10.1038/ncomms3771.
13. Köttgen, A., Albrecht, E., Teumer, A., Vitart, V., Krumsiek, J., Hundertmark, C., Pistis, G., Ruggiero, D., O'Seaghdha, C.M., Haller, T., et al. (2013). Genome-wide association analyses identify 18 new loci associated with serum urate concentrations. Nat. Genet. *45*, 145–154. https://doi.org/10.1038/ng.2500.
14. InanlooRahatloo, K., Liang, G., Vo, D., Ebert, A., Nguyen, I., and Nguyen, P.K. (2017). Sex-based differences in myocardial gene expression in recently deceased organ donors with no prior cardiovascular disease. PLoS One *12*, e0183874. https://doi.org/10.1371/journal.pone.0183874.
15. Melé, M., Ferreira, P.G., Reverter, F., DeLuca, D.S., Monlong, J., Sammeth, M., Young, T.R., Goldmann, J.M., Pervouchine, D.D., Sullivan, T.J., et al. (2015). Human genomics. The human transcriptome across tissues and individuals. Science *348*, 660–665. https://doi.org/10.1126/science.aaa0355.
16. Gershoni, M., and Pietrokovski, S. (2017). The landscape of sex-differential transcriptome and its consequent selection in human adults. BMC Biol. *15*, 7. https://doi.org/10.1186/s12915-017-0352-z.
17. Guo, S., Zhou, Y., Zeng, P., Xu, G., Wang, G., and Cui, Q. (2018). Identification and analysis of the human sex-biased genes. Briefings Bioinf. *19*, 188–198. https://doi.org/10.1093/bib/bbw125.
18. Khramtsova, E.A., Davis, L.K., and Stranger, B.E. (2019). The role of sex in the genomics of human complex traits. Nat. Rev. Genet. *20*, 173–190. https://doi.org/10.1038/s41576-018-0083-1.
19. Rawlik, K., Canela-Xandri, O., and Tenesa, A. (2016). Evidence for sex-specific genetic architectures across a spectrum of human complex traits. Genome Biol. *17*, 166. https://doi.org/10.1186/s13059-016-1025-x.
20. Gilks, W.P., Abbott, J.K., and Morrow, E.H. (2014). Sex differences in disease genetics: evidence, evolution, and detection. Trends Genet. *30*, 453–463. https://doi.org/10.1016/j.tig.2014.08.006.
21. Traglia, M., Bseiso, D., Gusev, A., Adviento, B., Park, D.S., Mefford, J.A., Zaitlen, N., and Weiss, L.A. (2017). Genetic

Mechanisms Leading to Sex Differences Across Common Diseases and Anthropometric Traits. Genetics *205*, 979–992. https://doi.org/10.1534/genetics.116.193623.

22. Ge, T., Chen, C.-Y., Neale, B.M., Sabuncu, M.R., and Smoller, J.W. (2017). Phenome-wide heritability analysis of the UK Biobank. PLoS Genet. *13*, e1006711. https://doi.org/10.1371/journal.pgen.1006711.
23. Yang, J., Bakshi, A., Zhu, Z., Hemani, G., Vinkhuyzen, A.A.E., Nolte, I.M., van Vliet-Ostaptchouk, J.V., Snieder, H., Lifelines Cohort Study, Esko, T., et al. (2015). Genome-wide genetic homogeneity between sexes and populations for human height and body mass index. Hum. Mol. Genet. *24*, 7445–7449. https://doi.org/10.1093/hmg/ddv443.
24. Stringer, S., Polderman, T.J.C., and Posthuma, D. (2017). Majority of human traits do not show evidence for sex-specific genetic and environmental effects. Sci. Rep. *7*, 8688. https://doi.org/10.1038/s41598-017-09249-3.
25. Vink, J.M., Bartels, M., van Beijsterveldt, T.C.E.M., van Dongen, J., van Beek, J.H.D.A., Distel, M.A., de Moor, M.H.M., Smit, D.J.A., Minica, C.C., Ligthart, L., et al. (2012). Sex differences in genetic architecture of complex phenotypes? PLoS One *7*, e47371. https://doi.org/10.1371/journal.pone.0047371.
26. Polderman, T.J.C., Benyamin, B., de Leeuw, C.A., Sullivan, P.F., van Bochoven, A., Visscher, P.M., and Posthuma, D. (2015). Meta-analysis of the heritability of human traits based on fifty years of twin studies. Nat. Genet. *47*, 702–709. https://doi.org/10.1038/ng.3285.
27. Winkler, T.W., Justice, A.E., Graff, M., Barata, L., Feitosa, M.F., Chu, S., Czajkowski, J., Esko, T., Fall, T., Kilpeläinen, T.O., et al. (2015). The Influence of Age and Sex on Genetic Associations with Adult Body Size and Shape: A Large-Scale Genome-Wide Interaction Study. PLoS Genet. *11*, e1005378. https://doi.org/10.1371/journal.pgen.1005378.
28. Pulit, S.L., Stoneman, C., Morris, A.P., Wood, A.R., Glastonbury, C.A., Tyrrell, J., Yengo, L., Ferreira, T., Marouli, E., Ji, Y., et al. (2019). Meta-analysis of genome-wide association studies for body fat distribution in 694 649 individuals of European ancestry. Hum. Mol. Genet. *28*, 166–174. https://doi.org/10.1093/hmg/ddy327.
29. Döring, A., Gieger, C., Mehta, D., Gohlke, H., Prokisch, H., Coassin, S., Fischer, G., Henke, K., Klopp, N., Kronenberg, F., et al. (2008). SLC2A9 influences uric acid concentrations with pronounced sex-specific effects. Nat. Genet. *40*, 430–436. https://doi.org/10.1038/ng.107.
30. Randall, J.C., Winkler, T.W., Kutalik, Z., Berndt, S.I., Jackson, A.U., Monda, K.L., Kilpeläinen, T.O., Esko, T., Mägi, R., Li, S., et al. (2013). Sex-stratified Genome-wide Association Studies Including 270,000 Individuals Show Sexual Dimorphism in Genetic Loci for Anthropometric Traits. PLoS Genet. *9*, e1003500. https://doi.org/10.1371/journal.pgen.1003500.
31. Rask-Andersen, M., Karlsson, T., Ek, W.E., and Johansson, Å. (2019). Genome-wide association study of body fat distribution identifies adiposity loci and sex-specific genetic effects. Nat. Commun. *10*, 339. https://doi.org/10.1038/s41467-018-08000-4.
32. Dumitrescu, L., Barnes, L.L., Thambisetty, M., Beecham, G., Kunkle, B., Bush, W.S., Gifford, K.A., Chibnik, L.B., Mukherjee, S., De Jager, P.L., et al. (2019). Sex differences in the genetic predictors of Alzheimer's pathology. Brain *142*, 2581–2589. https://doi.org/10.1093/brain/awz206.
33. Graham, S.E., Nielsen, J.B., Zawistowski, M., Zhou, W., Fritsche, L.G., Gabrielsen, M.E., Skogholt, A.H., Surakka, I., Hornsby, W.E., Fermin, D., et al. (2019). Sex-specific and pleiotropic effects underlying kidney function identified from GWAS meta-analysis. Nat. Commun. *10*, 1847. https://doi.org/10.1038/s41467-019-09861-z.
34. Hartiala, J.A., Tang, W.H.W., Wang, Z., Crow, A.L., Stewart, A.F.R., Roberts, R., McPherson, R., Erdmann, J., Willenborg, C., Hazen, S.L., and Allayee, H. (2016). Genome-wide association study and targeted metabolomics identifies sex-specific association of CPS1 with coronary artery disease. Nat. Commun. *7*, 10558. https://doi.org/10.1038/ncomms10558.
35. Beery, A.K., and Zucker, I. (2011). Sex bias in neuroscience and biomedical research. Neurosci. Biobehav. Rev. *35*, 565–572. https://doi.org/10.1016/j.neubiorev.2010.07.002.
36. Vandenbroeck, K. (2012). Cytokine Gene Polymorphisms and Human Autoimmune Disease in the Era of Genome-Wide Association Studies. J. Interferon Cytokine Res. *32*, 139–151. https://doi.org/10.1089/jir.2011.0103.
37. Lippitz, B.E. (2013). Cytokine patterns in patients with cancer: a systematic review. Lancet Oncol. *14*, e218–e228. https://doi.org/10.1016/S1470-2045(12)70582-X.
38. Dinarello, C.A. (2003). Anti-cytokine therapeutics and infections. Vaccine *21*, S24–S34. https://doi.org/10.1016/S0264-410X(03)00196-8.
39. Boahen, C.K., Temba, G.S., Kullaya, V.I., Matzaraki, V., Joosten, L.A.B., Kibiki, G., Mmbaga, B.T., van der Ven, A., de Mast, Q., Netea, M.G., and Kumar, V. (2022). A functional genomics approach in Tanzanian population identifies distinct genetic regulators of cytokine production compared to European population. Am. J. Hum. Genet. *109*, 471–485. https://doi.org/10.1016/j.ajhg.2022.01.014.
40. Schirmer, M., Smeekens, S.P., Vlamakis, H., Jaeger, M., Oosting, M., Franzosa, E.A., Horst, R.T., Jansen, T., Jacobs, L., Bonder, M.J., et al. (2016). Linking the Human Gut Microbiome to Inflammatory Cytokine Production Capacity. Cell *167*, 1897. https://doi.org/10.1016/j.cell.2016.11.046.
41. Li, Y., Oosting, M., Deelen, P., Ricaño-Ponce, I., Smeekens, S., Jaeger, M., Matzaraki, V., Swertz, M.A., Xavier, R.J., Franke, L., et al. (2016). Inter-individual variability and genetic influences on cytokine responses to bacteria and fungi. Nat. Med. *22*, 952–960. https://doi.org/10.1038/nm.4139.
42. Li, Y., Oosting, M., Smeekens, S.P., Jaeger, M., Aguirre-Gamboa, R., Le, K.T.T., Deelen, P., Ricaño-Ponce, I., Schoffelen, T., Jansen, A.F.M., et al. (2016). A Functional Genomics Approach to Understand Variation in Cytokine Production in Humans. Cell *167*, 1099–1110.e14. https://doi.org/10.1016/j.cell.2016.10.017.
43. Temba, G.S., Kullaya, V., Pecht, T., Mmbaga, B.T., Aschenbrenner, A.C., Ulas, T., Kibiki, G., Lyamuya, F., Boahen, C.K., Kumar, V., et al. (2021). Urban living in healthy Tanzanians is associated with an inflammatory status driven by dietary and metabolic changes. Nat. Immunol. *22*, 287–300. https://doi.org/10.1038/s41590-021-00867-8.
44. Shah, T.S., Liu, J.Z., Floyd, J.A.B., Morris, J.A., Wirth, N., Barrett, J.C., and Anderson, C.A. (2012). optiCall: a robust genotype-calling algorithm for rare, low-frequency and common variants. Bioinforma. Oxf. Engl. *28*, 1598–1603. https://doi.org/10.1093/bioinformatics/bts180.
45. Das, S., Forer, L., Schönherr, S., Sidore, C., Locke, A.E., Kwong, A., Vrieze, S.I., Chew, E.Y., Levy, S., McGue, M., et al. (2016). Next-generation genotype imputation service and methods. Nat. Genet. *48*, 1284–1287. https://doi.org/10.1038/ng.3656.
46. Deelen, P., Bonder, M.J., van der Velde, K.J., Westra, H.-J., Winder, E., Hendriksen, D., Franke, L., and Swertz, M.A.

(2014). Genotype harmonizer: automatic strand alignment and format conversion for genotype data integration. BMC Res. Notes *7*, 901. https://doi.org/10.1186/1756-0500-7-901.

47. Aulchenko, Y.S., Ripke, S., Isaacs, A., and van Duijn, C.M. (2007). GenABEL: an R library for genome-wide association analysis. Bioinforma. Oxf. Engl. *23*, 1294–1296. https://doi.org/10.1093/bioinformatics/btm108.
48. Shabalin, A.A. (2012). Matrix eQTL: ultra fast eQTL analysis via large matrix operations. Bioinformatics *28*, 1353–1358. https://doi.org/10.1093/bioinformatics/bts163.
49. Purcell, S., Neale, B., Todd-Brown, K., Thomas, L., Ferreira, M.A.R., Bender, D., Maller, J., Sklar, P., De Bakker, P.I.W., Daly, M.J., and Sham, P.C. (2007). PLINK: A Tool Set for Whole-Genome Association and Population-Based Linkage Analyses. Am. J. Hum. Genet. *81*, 559–575. https://doi.org/10.1086/519795.
50. Giambartolomei, C., Vukcevic, D., Schadt, E.E., Franke, L., Hingorani, A.D., Wallace, C., and Plagnol, V. (2014). Bayesian Test for Colocalisation between Pairs of Genetic Association Studies Using Summary Statistics. PLoS Genet. *10*, e1004383. https://doi.org/10.1371/journal.pgen.1004383.
51. Boughton, A.P., Welch, R.P., Flickinger, M., VandeHaar, P., Taliun, D., Abecasis, G.R., and Boehnke, M. (2021). LocusZoom.js: interactive and embeddable visualization of genetic association study results. Bioinformatics *37*, 3017–3018. https://doi.org/10.1093/bioinformatics/btab186.
52. Kamat, M.A., Blackshaw, J.A., Young, R., Surendran, P., Burgess, S., Danesh, J., Butterworth, A.S., and Staley, J.R. (2019). PhenoScanner V2: an expanded tool for searching human genotype–phenotype associations. Bioinformatics *35*, 4851–4853. https://doi.org/10.1093/bioinformatics/btz469.
53. Staley, J.R., Blackshaw, J., Kamat, M.A., Ellis, S., Surendran, P., Sun, B.B., Paul, D.S., Freitag, D., Burgess, S., Danesh, J., et al. (2016). PhenoScanner: a database of human genotype-phenotype associations. Bioinforma. Oxf. Engl. *32*, 3207–3209. https://doi.org/10.1093/bioinformatics/btw373.
54. Yun, S., Lee, S.H., Yoon, S.-R., Kim, M.S., Piao, Z.-H., Myung, P.-K., Kim, T.-D., Jung, H., and Choi, I. (2011). TOX regulates the differentiation of human natural killer cells from hematopoietic stem cells in vitro. Immunol. Lett. *136*, 29–36. https://doi.org/10.1016/j.imlet.2010.11.008.
55. Bell, D.W., Brannigan, B.W., Matsuo, K., Finkelstein, D.M., Sordella, R., Settleman, J., Mitsudomi, T., and Haber, D.A. (2008). Increased Prevalence of EGFR-Mutant Lung Cancer in Women and in East Asian Populations: Analysis of Estrogen-Related Polymorphisms. Clin. Cancer Res. *14*, 4079–4084. https://doi.org/10.1158/1078-0432.CCR-07-5030.
56. Gaur, P., Bhattacharya, S., Kant, S., Kushwaha, R.A.S., Singh, G., and Pandey, S. (2018). EGFR Mutation Detection and Its Association With Clinicopathological Characters of Lung Cancer Patients. World J. Oncol. *9*, 151–155. https://doi.org/10.14740/wjon1167.
57. Gu, H., Hou, F., Liu, L., Luo, X., Nkomola, P.D., Xie, X., Li, X., and Song, R. (2018). Genetic variants in the CNTNAP2 gene are associated with gender differences among dyslexic children in China. EBioMedicine *34*, 165–170. https://doi.org/10.1016/j.ebiom.2018.07.007.
58. Paul, D., Islam, S., Manne, R.K., Dinesh, U.S., Malonia, S.K., Maity, B., Boppana, R., Rapole, S., Shetty, P.K., and Santra, M.K. (2019). F-box protein FBXO16 functions as a tumor suppressor by attenuating nuclear β-catenin function. J. Pathol. *248*, 266–279. https://doi.org/10.1002/path.5252.
59. Foxman, B. (2014). Urinary tract infection syndromes: occurrence, recurrence, bacteriology, risk factors, and disease burden. Infect. Dis. Clin. *28*, 1–13. https://doi.org/10.1016/j.idc.2013.09.003.
60. Williams, G., Hahn, D., Stephens, J.H., Craig, J.C., and Hodson, E.M. (2023). Cranberries for preventing urinary tract infections. Cochrane Database Syst. Rev. *4*, CD001321. https://doi.org/10.1002/14651858.CD001321.pub6.
61. Burnier, M., Bakris, G., and Williams, B. (2019). Redefining diuretics use in hypertension: why select a thiazide-like diuretic? J. Hypertens. *37*, 1574–1586. https://doi.org/10.1097/HJH.0000000000002088.
62. Kong, S., and Cho, Y.S. (2019). Identification of female-specific genetic variants for metabolic syndrome and its component traits to improve the prediction of metabolic syndrome in females. BMC Med. Genet. *20*, 99. https://doi.org/10.1186/s12881-019-0830-y.
63. Gautam, Y., Afanador, Y., Abebe, T., López, J.E., and Mersha, T.B. (2019). Genome-wide analysis revealed sex-specific gene expression in asthmatics. Hum. Mol. Genet. *28*, 2600–2614. https://doi.org/10.1093/hmg/ddz074.
64. Ogata, H., Goto, S., Sato, K., Fujibuchi, W., Bono, H., and Kanehisa, M. (1999). KEGG: Kyoto Encyclopedia of Genes and Genomes. Nucleic Acids Res. *27*, 29–34. https://doi.org/10.1093/nar/27.1.29.
65. Kanehisa, M., Furumichi, M., Tanabe, M., Sato, Y., and Morishima, K. (2017). KEGG: new perspectives on genomes, pathways, diseases and drugs. Nucleic Acids Res. *45*, D353–D361. https://doi.org/10.1093/nar/gkw1092.
66. Jassal, B., Matthews, L., Viteri, G., Gong, C., Lorente, P., Fabregat, A., Sidiropoulos, K., Cook, J., Gillespie, M., Haw, R., et al. (2020). The reactome pathway knowledgebase. Nucleic Acids Res. *48*, D498–D503. gkz1031. https://doi.org/10.1093/nar/gkz1031.
67. Gillespie, M., Jassal, B., Stephan, R., Milacic, M., Rothfels, K., Senff-Ribeiro, A., Griss, J., Sevilla, C., Matthews, L., Gong, C., et al. (2022). The reactome pathway knowledgebase 2022. Nucleic Acids Res. *50*, D687–D692. https://doi.org/10.1093/nar/gkab1028.
68. Diedisheim, M., Carcarino, E., Vandiedonck, C., Roussel, R., Gautier, J.-F., and Venteclef, N. (2020). Regulation of inflammation in diabetes: From genetics to epigenomics evidence. Mol. Metabol. *41*, 101041. https://doi.org/10.1016/j.molmet.2020.101041.
69. Hug, H., Mohajeri, M.H., and La Fata, G. (2018). Toll-Like Receptors: Regulators of the Immune Response in the Human Gut. Nutrients *10*, 203. https://doi.org/10.3390/nu10020203.
70. Boivin, G.A., Pothlichet, J., Skamene, E., Brown, E.G., Loredo-Osti, J.C., Sladek, R., and Vidal, S.M. (2012). Mapping of Clinical and Expression Quantitative Trait Loci in a Sex-Dependent Effect of Host Susceptibility to Mouse-Adapted Influenza H3N2/HK/1/68. J. Immunol. *188*, 3949–3960. https://doi.org/10.4049/jimmunol.1103320.
71. Hayes, K.S., Hager, R., and Grencis, R.K. (2014). Sex-dependent genetic effects on immune responses to a parasitic nematode. BMC Genom. *15*, 193. https://doi.org/10.1186/1471-2164-15-193.
72. Mori, A., Hane, M., Niimi, Y., Kitajima, K., and Sato, C. (2017). Different properties of polysialic acids synthesized by the polysialyltransferases ST8SIA2 and ST8SIA4. Glycobiology *27*, 834–846. https://doi.org/10.1093/glycob/cwx057.

73. Ji, M., Zhao, Z., Li, Y., Xu, P., Shi, J., Li, Z., Wang, K., Huang, X., Ji, J., Liu, W., and Liu, B. (2021). FBXO16-mediated hnRNPL ubiquitination and degradation plays a tumor suppressor role in ovarian cancer. Cell Death Dis. *12*, 758. https://doi.org/10.1038/s41419-021-04040-9.
74. Gerke, V., Gavins, F.N.E., Geisow, M., Grewal, T., Jaiswal, J.K., Nylandsted, J., and Rescher, U. (2024). Annexins—a family of proteins with distinctive tastes for cell signaling and membrane dynamics. Nat. Commun. *15*, 1574. https://doi.org/10.1038/s41467-024-45954-0.
75. Abou-Ismail, M.Y., Citla Sridhar, D., and Nayak, L. (2020). Estrogen and thrombosis: A bench to bedside review. Thromb. Res. *192*, 40–51. https://doi.org/10.1016/j.thromres.2020.05.008.
76. Areal, H., Abrantes, J., and Esteves, P.J. (2011). Signatures of positive selection in Toll-like receptor (TLR) genes in mammals. BMC Evol. Biol. *11*, 368. https://doi.org/10.1186/1471-2148-11-368.
77. Laayouni, H., Oosting, M., Luisi, P., Ioana, M., Alonso, S., Ricaño-Ponce, I., Trynka, G., Zhernakova, A., Plantinga, T.S., Cheng, S.-C., et al. (2014). Convergent evolution in European and Rroma populations reveals pressure exerted by plague on Toll-like receptors. Proc. Natl. Acad. Sci. USA *111*, 2668–2673. https://doi.org/10.1073/pnas.1317723111.
78. Fairweather, D., Beetler, D.J., McCabe, E.J., and Lieberman, S.M. (2024). Mechanisms underlying sex differences in autoimmunity. J. Clin. Investig. *134*, e180076. https://doi.org/10.1172/JCI180076.
79. Hay, M., Kumar, V., and Ricaño-Ponce, I. (2022). The role of the X chromosome in infectious diseases. Brief. Funct. Genom. *21*, 143–158. https://doi.org/10.1093/bfgp/elab039.
80. Kastrati, L., Groothof, D., Quezada-Pinedo, H.G., Raeisi-Dehkordi, H., Bally, L., De Borst, M.H., Bakker, S.J.L., Vidal, P.-M., Eisenga, M.F., and Muka, T. (2024). Utility of iron biomarkers in differentiating menopausal status: Findings from CoLaus and PREVEND. Maturitas *179*, 107872. https://doi.org/10.1016/j.maturitas.2023.107872.
81. Cheng, S., Ning, Z., Huang, K., Yuan, Y., Tan, X., Pan, Y., Zhang, R., Tian, L., Lu, Y., Wang, X., et al. (2024). Analysis of sex-biased gene expression in a Eurasian admixed population. Briefings Bioinf. *25*, bbae451. https://doi.org/10.1093/bib/bbae451.
82. Oliva, M., Muñoz-Aguirre, M., Kim-Hellmuth, S., Wucher, V., Gewirtz, A.D.H., Cotter, D.J., Parsana, P., Kasela, S., Balliu, B., Viñuela, A., et al. (2020). The impact of sex on gene expression across human tissues. Science *369*, eaba3066. https://doi.org/10.1126/science.aba3066.

HGGA, Volume 7

Supplemental information

Sex-stratified genetic regulators of cytokine production in the Dutch and Tanzanian populations

Caroline Amour, Raul Cetatean, Isis Ricano Ponce, Nick Keur, Godfrey S. Temba, Vesla I. Kullaya, Blandina T. Mmbaga, Reginald Kavishe, Leo A.B. Joosten, Mihai G. Netea, Quirijn de Mast, Collins K. Boahen, and Vinod Kumar

Supplemental Figures

A

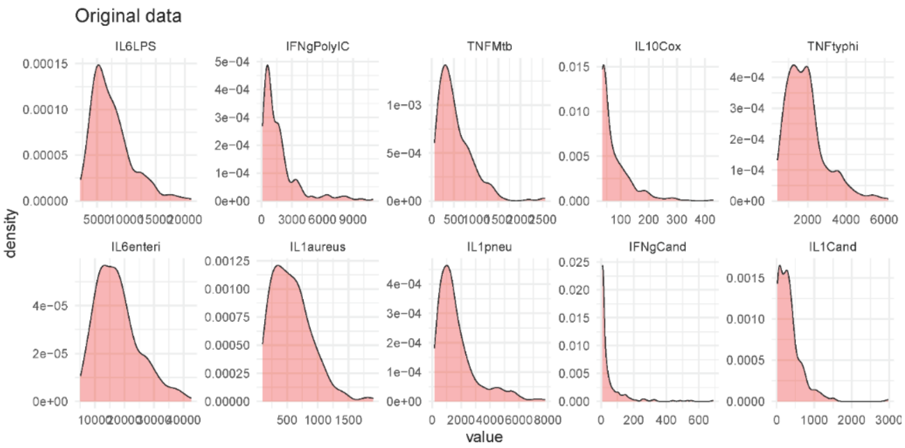

B

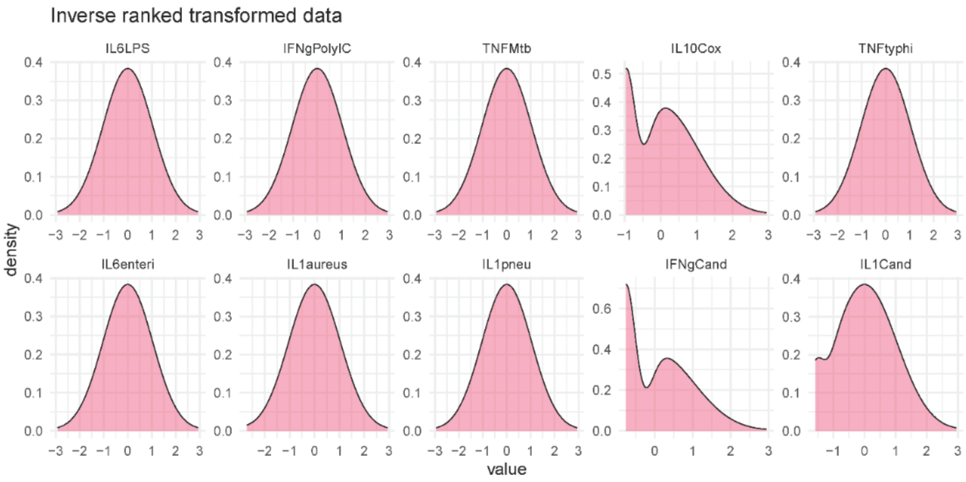

Figure S1: Distribution of the measured cytokine stimuli pairs for the Tanzania cohort.

Density plots depicting the distributions of cytokine production upon stimulation of various bacteria and pathogens. The density plots are arranged as follows A) raw (Original data), and B) inverse-ranked normalization of the cytokine production distributions respectively.

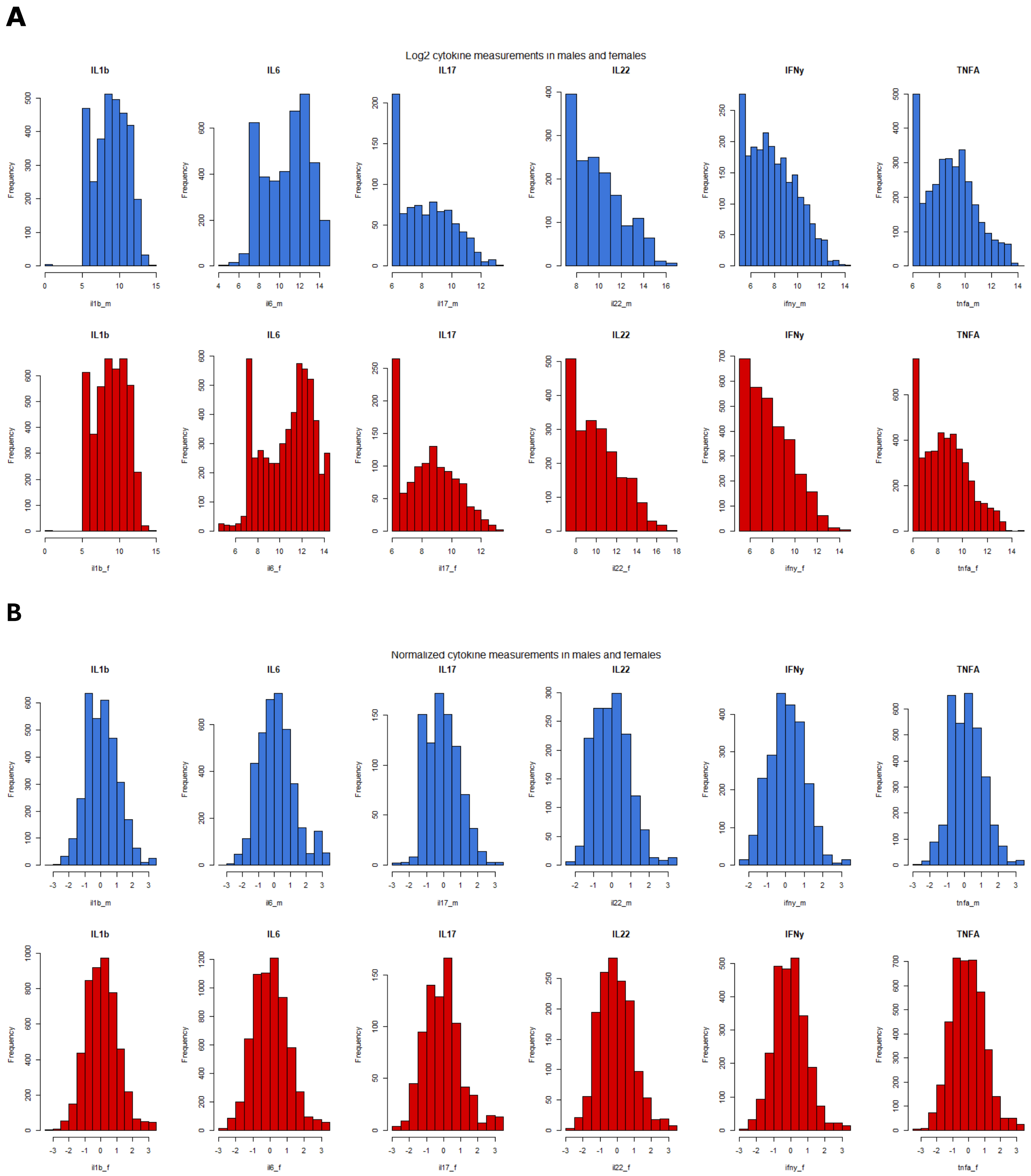

Figure S2: Distribution of the measured cytokine stimuli pairs for the Dutch cohort.

Density plots depicting the distributions of cytokine production upon stimulation of various bacteria and pathogens. The density plots are arranged as follows: A) raw (Original data), and B) inverse-ranked normalization of the cytokine production distributions respectively.

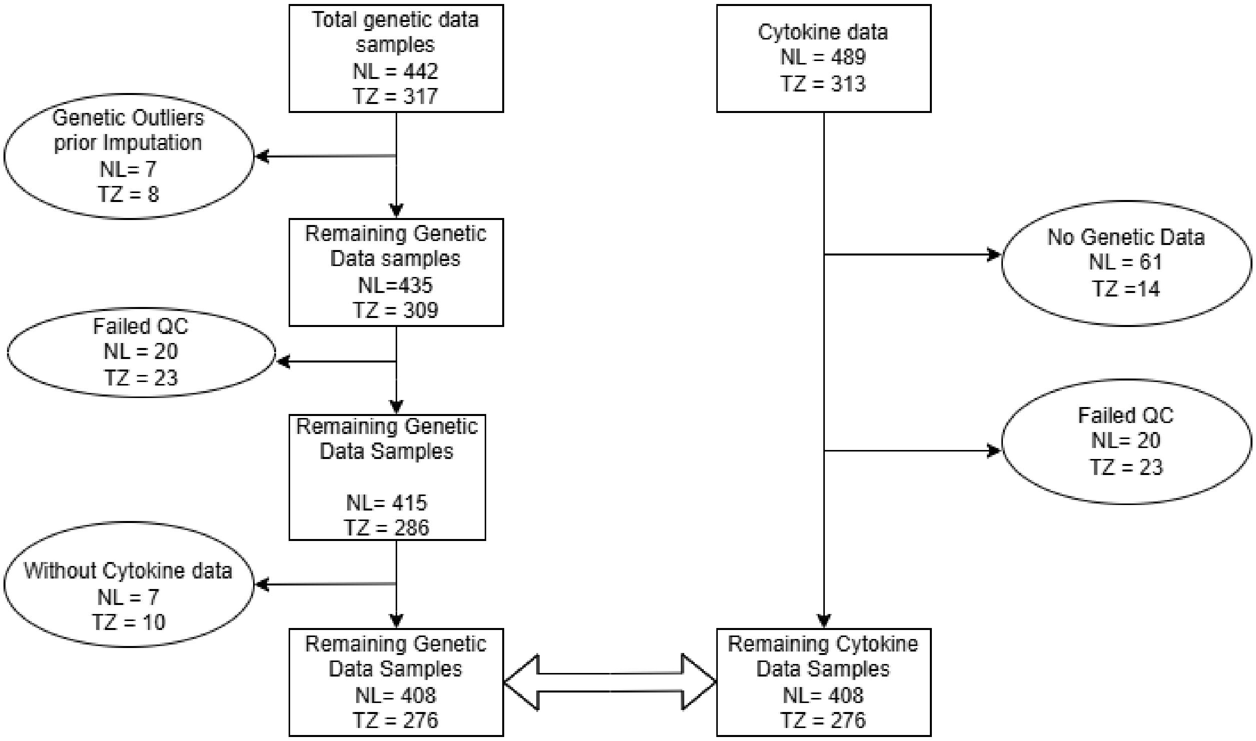

Figure S3: Visualization of samples in the Dutch (NL) and Tanzania (TZ) cohorts

Schematic diagram showing the sample preprocessing according to the measured genetic and cytokine data

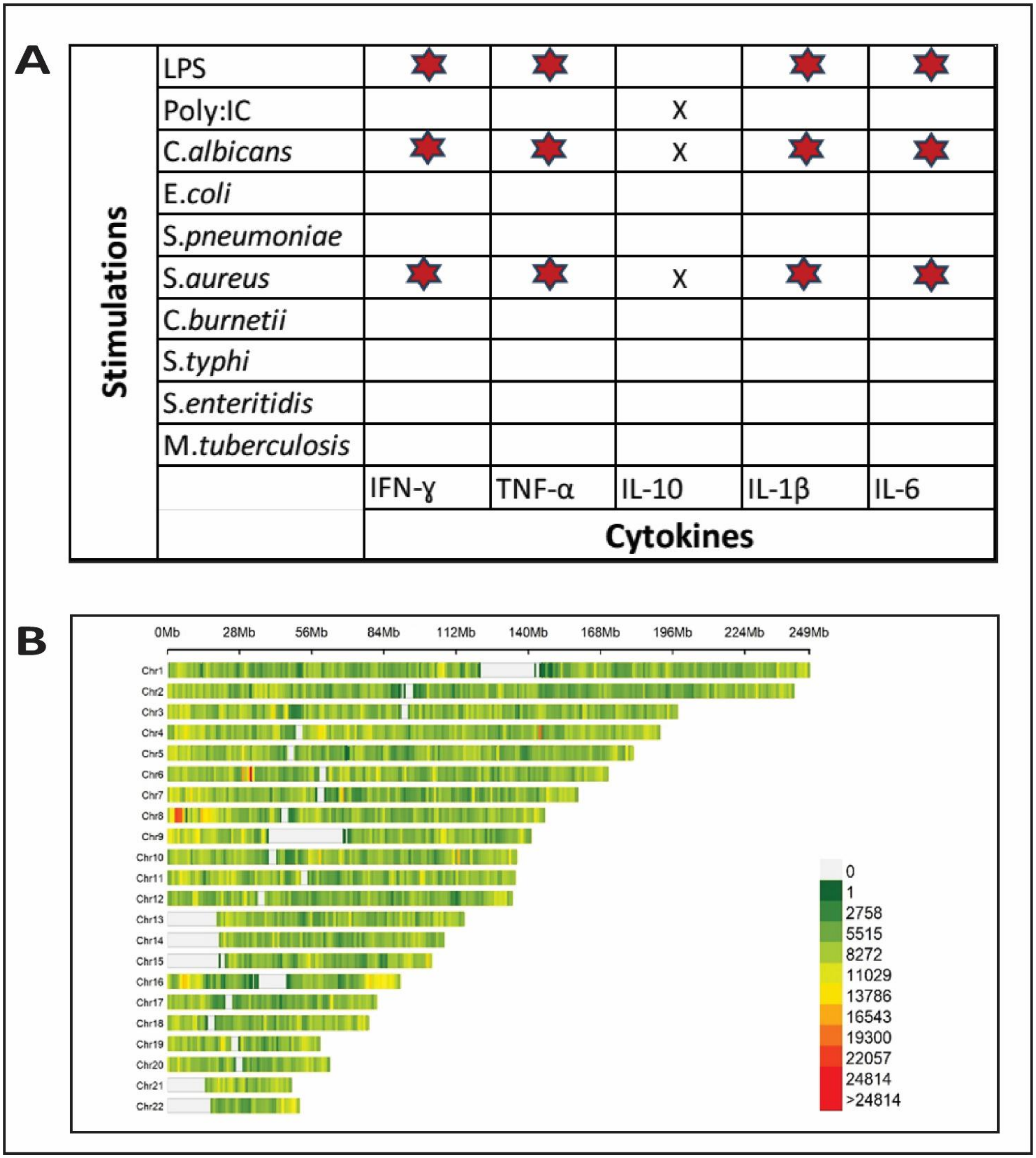

Figure S4: Cytokine-stimulation combinations and SNP density plot

(A) Illustration of cytokine-stimulation combinations common in both African and European datasets (indicated by red stars). Cells with "X'' labels denote cytokine-stimulation pairs excluded from downstream analysis, as more than 75% of samples had values below the detection limit of the assay.

(B) The SNP density plot chromosome wise representing number of SNPs within 1 Mb window size. The horizontal axis shows the chromosome length (Mb); the different color depicts SNP density

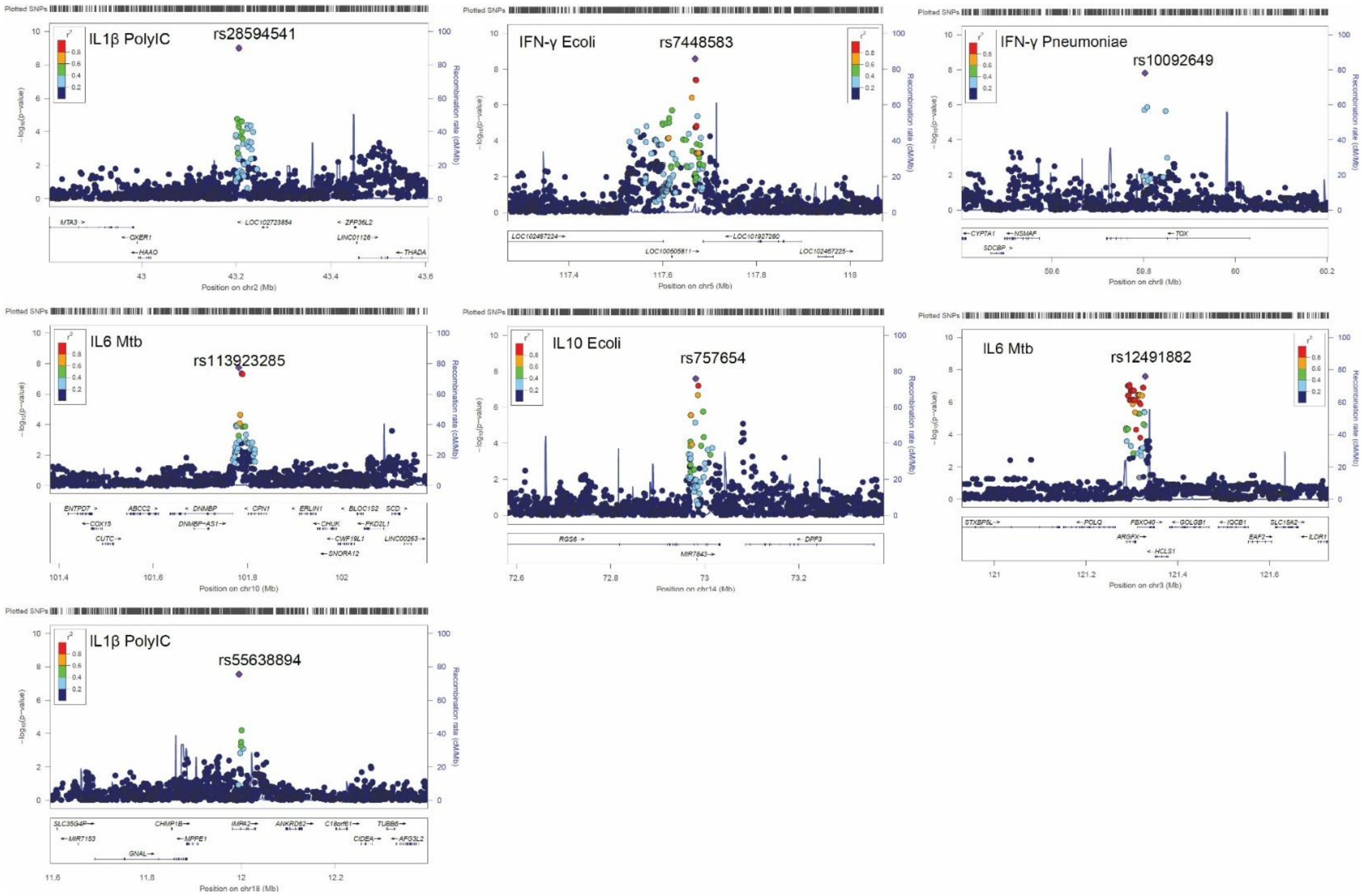

Figure S5: Regional association plot for each genome-wide cytokine QTL in the Tanzanian cohort males. Other SNPs within a genomic window of 400 kb are color-coded based on their linkage disequilibrium (r2) with the top SNP, displayed in purple. The horizontal axis represents chromosomal positions according to the NCBI human genome build 37, and the vertical axis represents -log10 p-values.

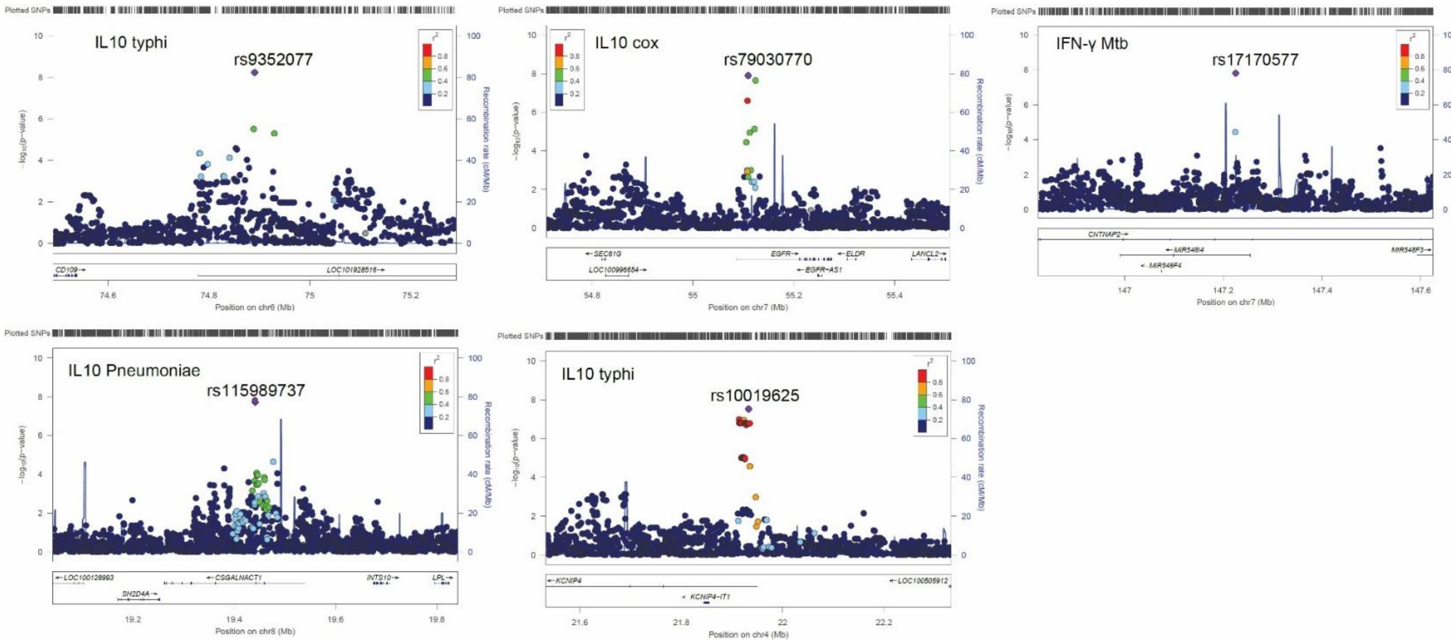

Figure S6: Regional association plot for each genome-wide cytokine QTL in the Tanzanian cohort females. Other SNPs within a genomic window of 400 kb are color-coded based on their linkage disequilibrium (r2) with the top SNP, displayed in purple. The horizontal axis represents chromosomal positions according to the NCBI human genome build 37, and the vertical axis represents -log10 p-values.

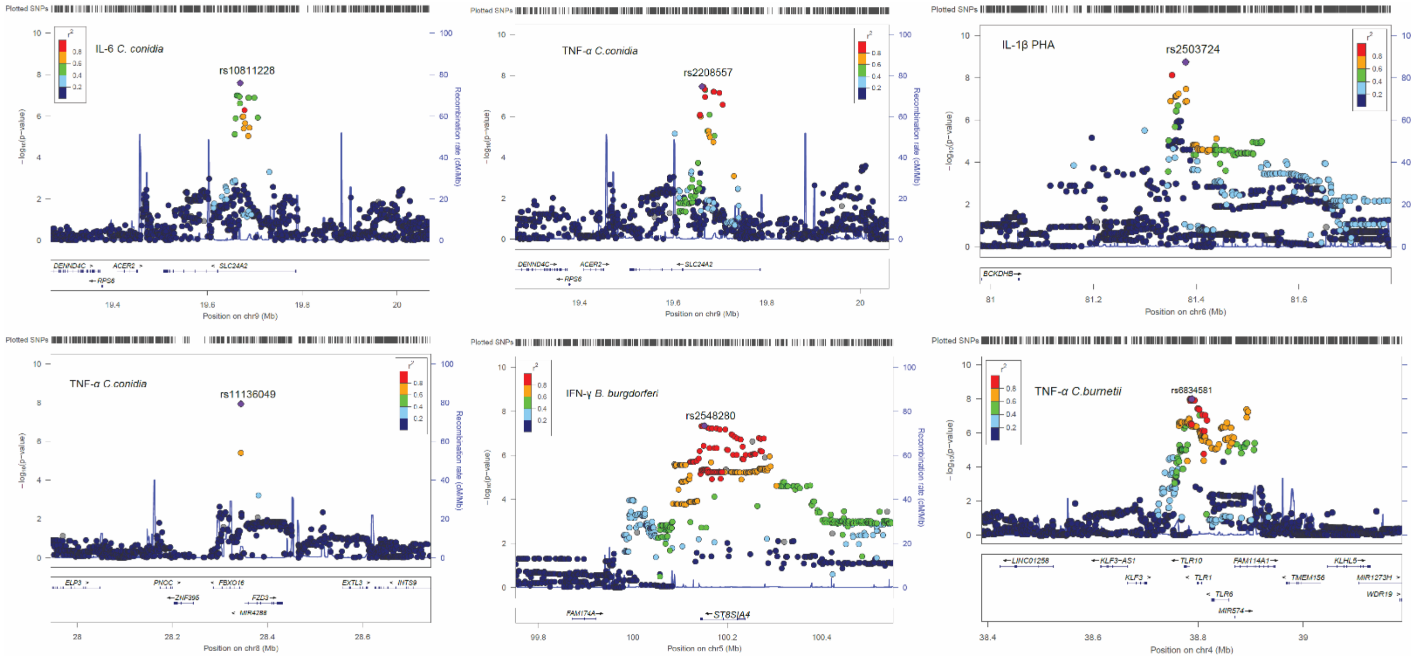

Figure S7: Regional association plot for each genome-wide cytokine QTL in the Dutch cohort males. Other SNPs within a genomic window of 400 kb are color-coded based on their linkage

disequilibrium (r2) with the top SNP, displayed in purple. The horizontal axis represents chromosomal positions according to the NCBI human genome build 37, and the vertical axis represents -log10 p-values.

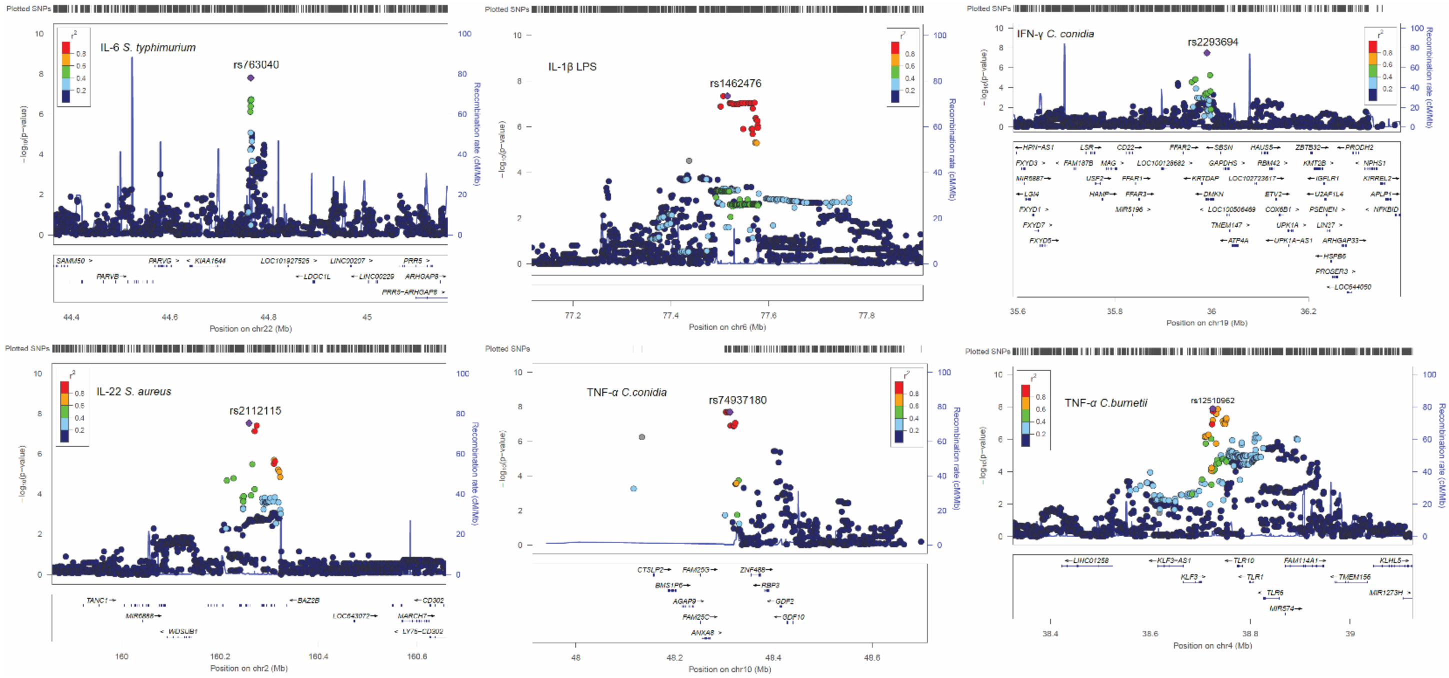

Figure S8: Regional association plot for each genome-wide cytokine QTL among females in the Dutch cohort. Other SNPs within a genomic window of 400 kb are color-coded based on their linkage disequilibrium (r2) with the top SNP, displayed in purple. The horizontal axis represents chromosomal positions according to the NCBI human genome build 37, and the vertical axis represents -log10 p-values.

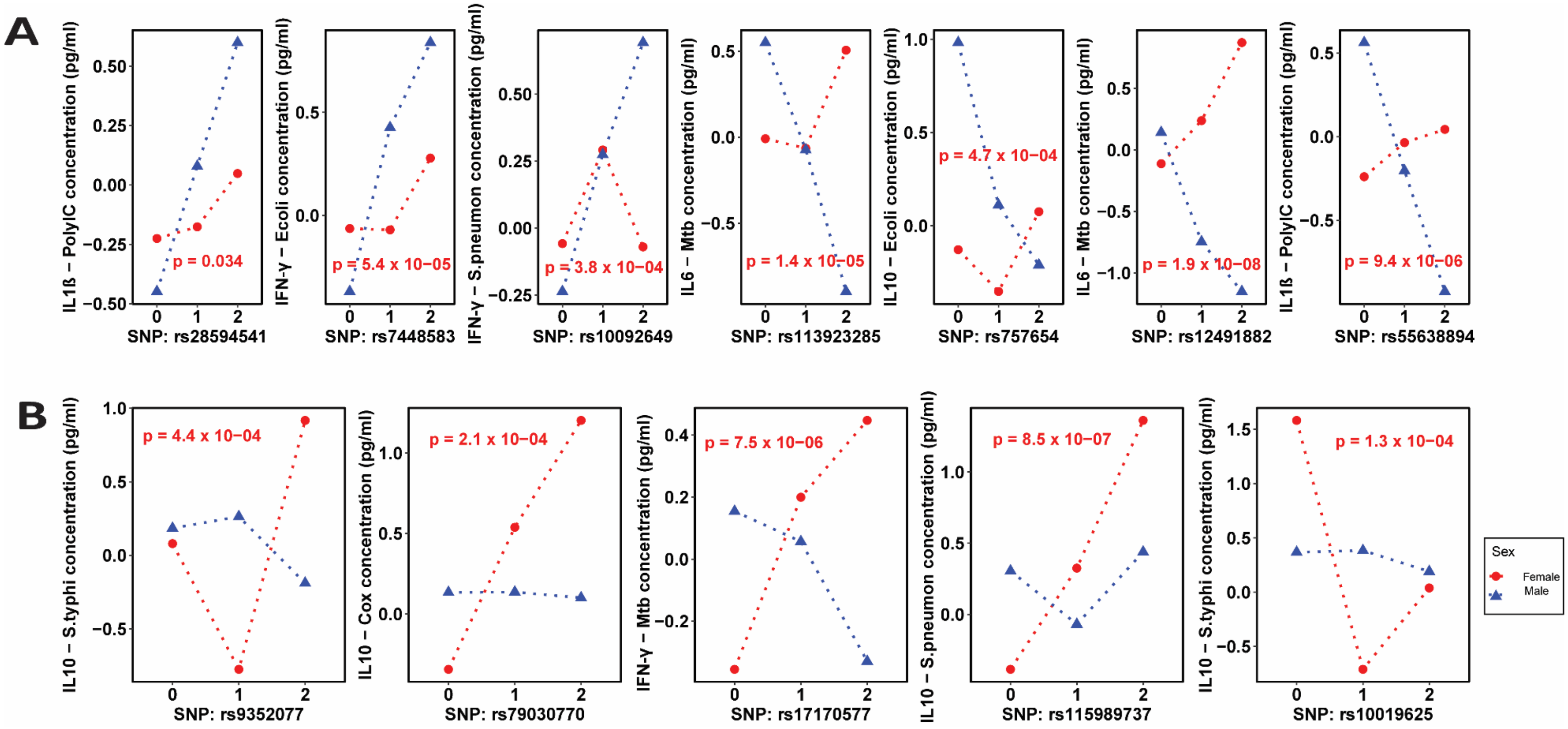

Figure S9: Visualization of Sex-by-SNP interaction in the Tanzanian cohort

(A) Interaction plots of the male-specific genome-wide significant cQTL variants.

(B) Interaction plots of the female-specific genome-wide significant cQTL variants. The median values of the cytokines are depicted with circle and triangle points for males and females respectively.

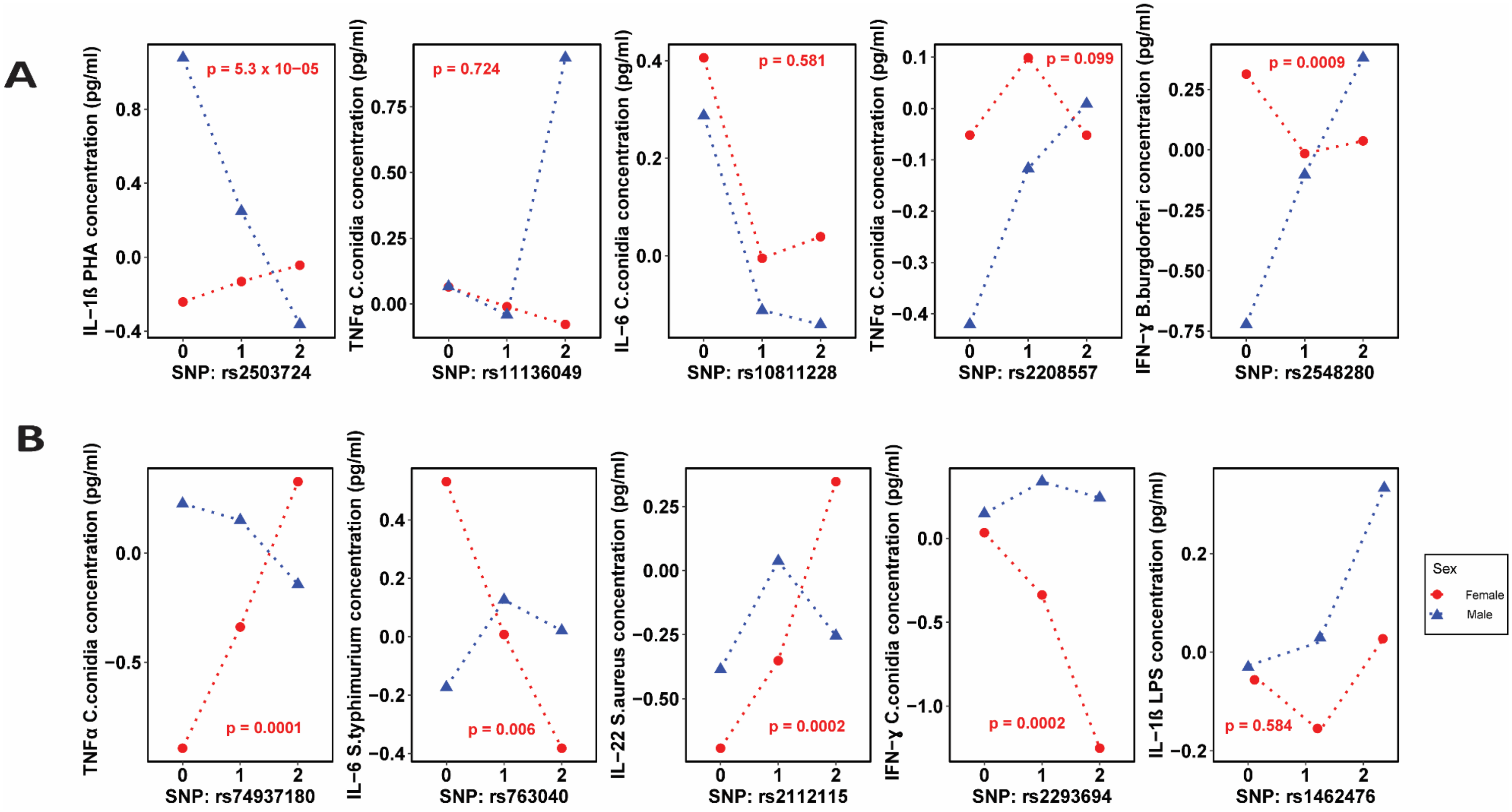

Figure S10: Visualization of Sex-by-SNP interaction in the Dutch cohort

(A) Interaction plots of the male-specific genome-wide significant cQTL variants.

(B) Interaction plots of the female-specific genome-wide significant cQTL variants. The median values of the cytokines are depicted with circle and triangle points for males and females respectively.

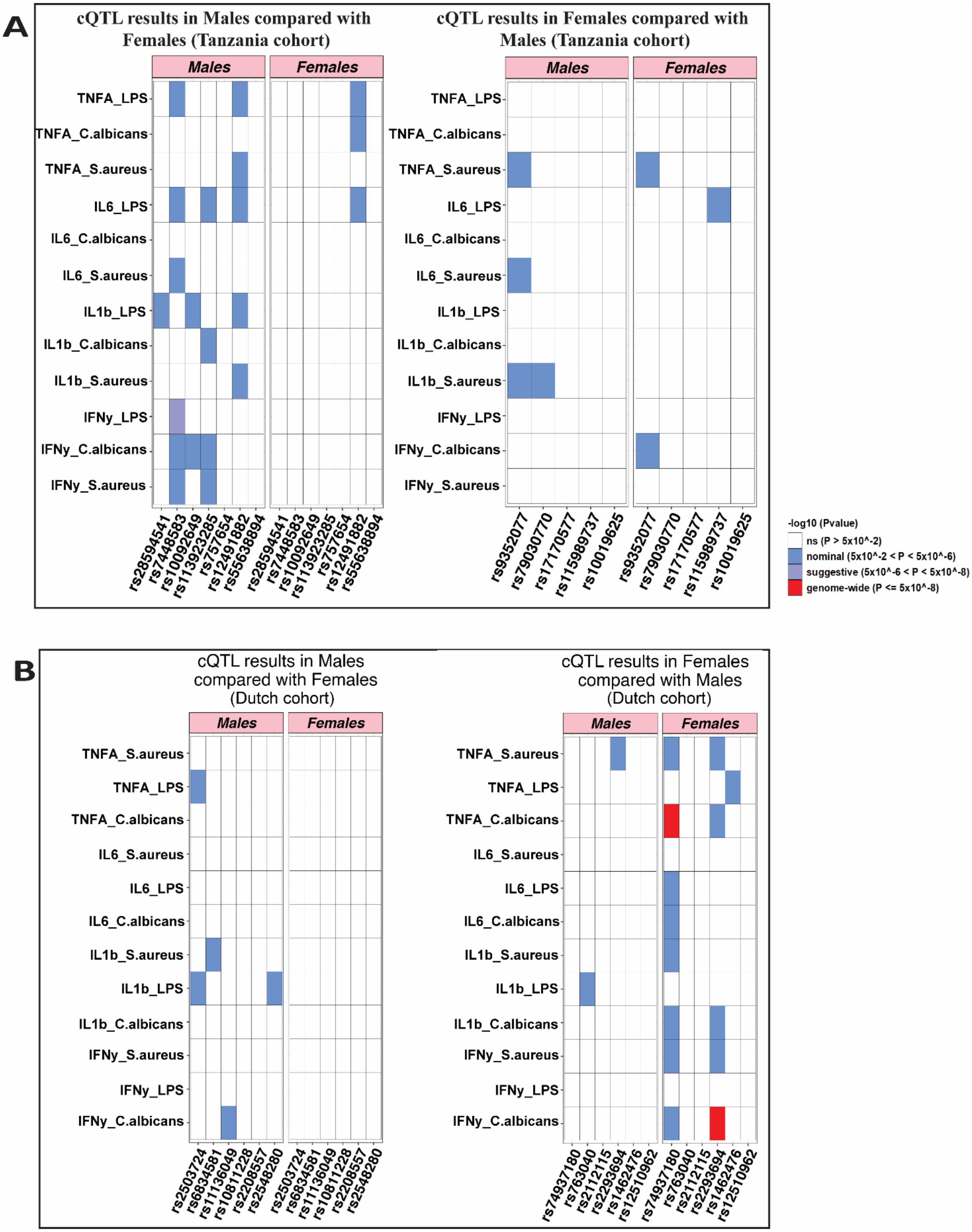

Figure S11: The association results of the cQTLs with the common cytokine measurements between the two cohorts are shown for A) Tanzania and B) Dutch cohorts. The color key indicates the range of cQTL p values (shown as -log10 Pvalue). The x axis shows the genome-

wide significant loci, and the y axis shows the cytokine-stimuli pairs. For each SNP, the -log10(p-value) is shown both for males and for females. Color-key ranges are: not significant - white, nominal - blue, suggestive – grey, and all the genome-wide significant associations are shown in red.

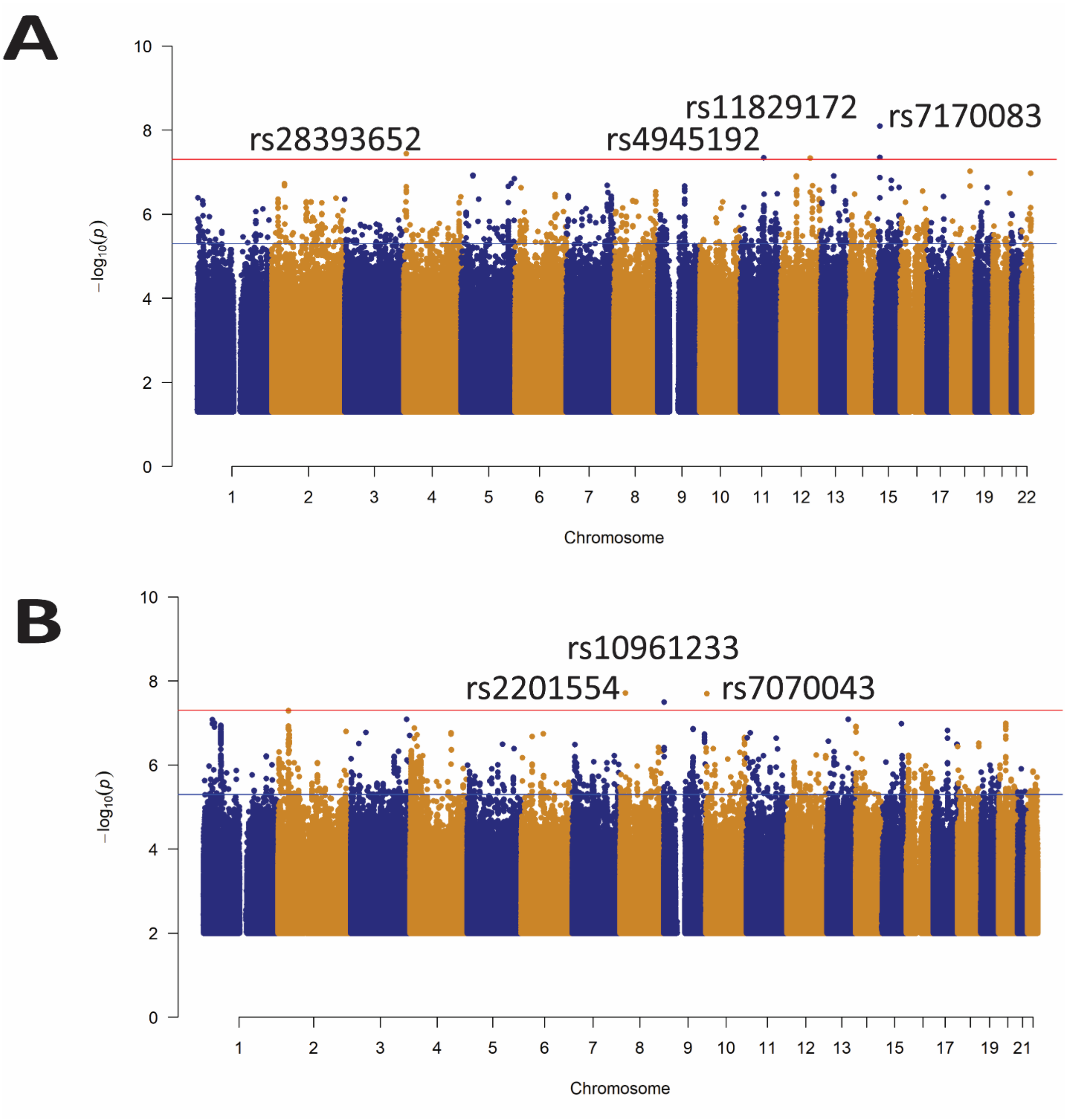

Figure S12: Manhattan plots of the c-QTL variants joint analysis of males and females in the Tanzania and Dutch cohorts.

The Manhattan plots show cQTLs that reached genome-wide significance in A) Participants upon stimulation in the Tanzania cohort and B) Participants in the Dutch cohort. The red horizontal dashed line represents the genome-wide significant threshold (p value < 5 x 10^{-8})

and the blue dashed line denotes the suggestive evidence of association threshold. Nominally significant (p value < 5 x 10^{-2}) cQTLs are plotted.

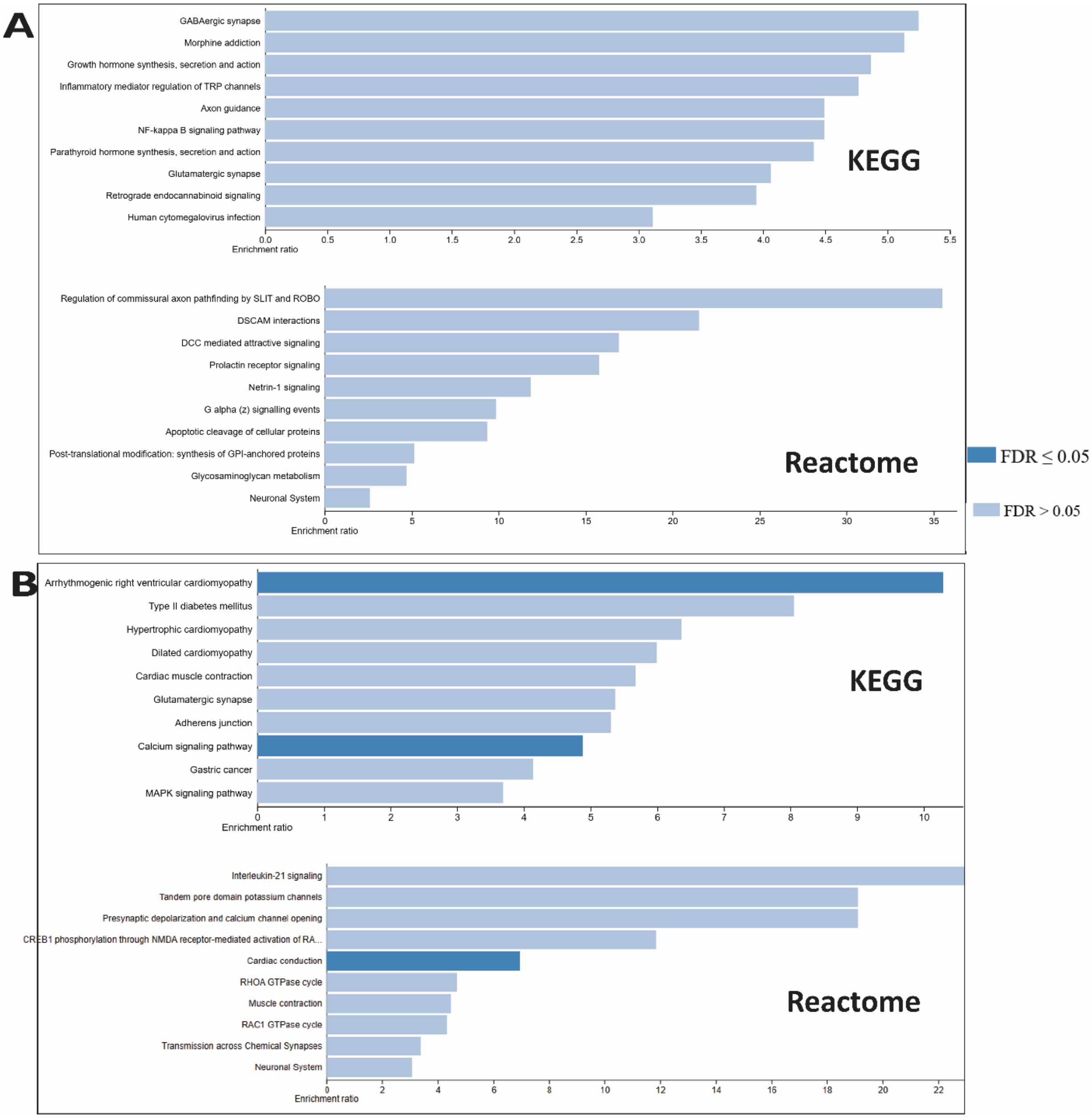

Figure S13: Bar graphs summarizing pathway enrichment results in the Tanzanian cohort

(A) Top enriched KEGG and Reactome pathways for gene sets curated from SNPs associated with cytokines in males.

(B) Top enriched KEGG and Reactome pathways for gene sets curated from SNPs associated with cytokines in females.

The bars represent statistically significant pathways after multiple testing. We set the false discovery rate (FDR)-adjusted p-value to 0.05 as the significance level.

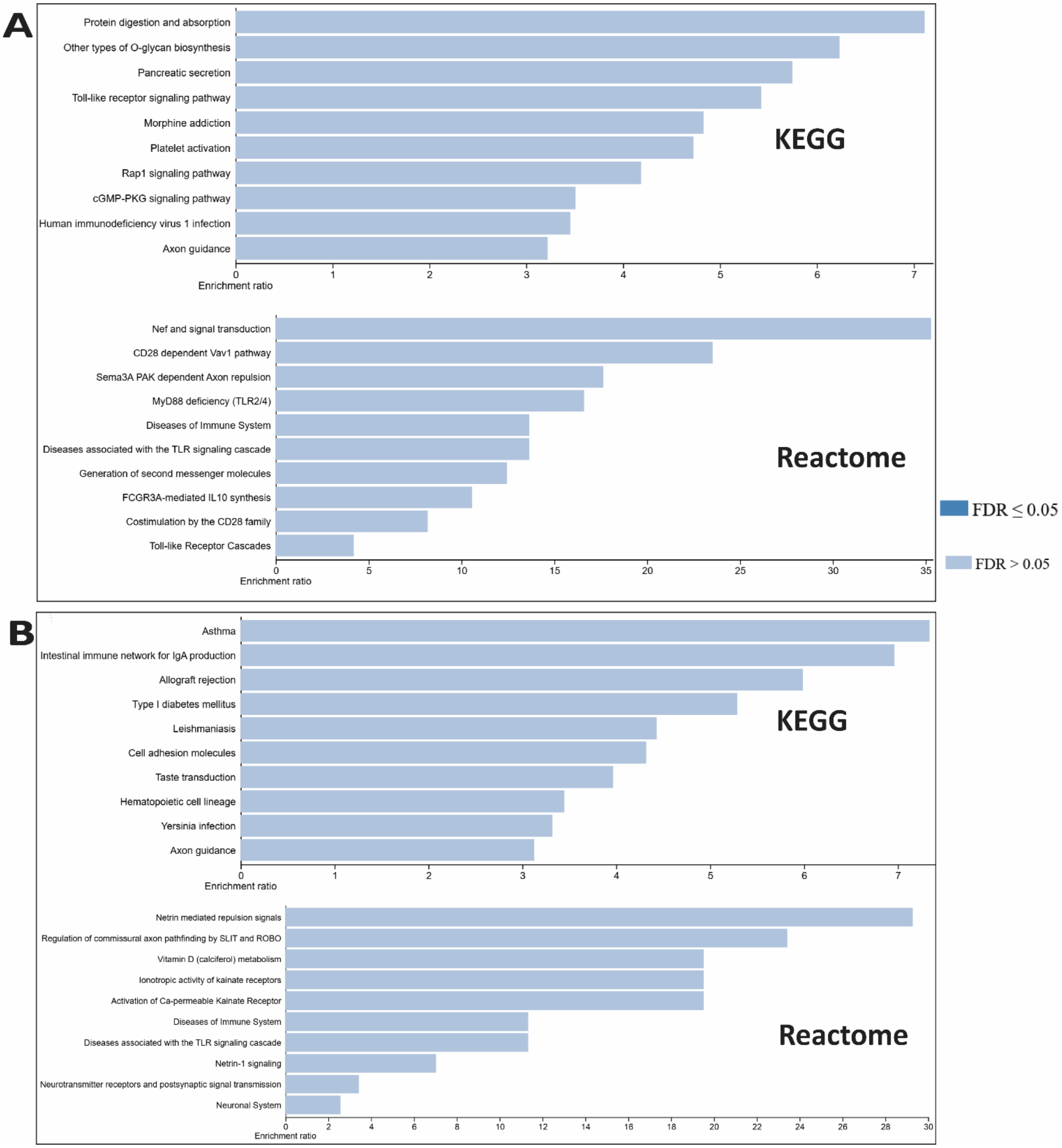

Figure S14: Bar graphs summarizing pathway enrichment results in the Dutch cohort

(A) Top enriched KEGG and Reactome pathways for gene sets curated from SNPs associated with cytokines in males.

(B) Top enriched KEGG and Reactome pathways for gene sets curated from SNPs associated with cytokines in females. The bars represent statistically significant pathways after multiple testing. We set the false discovery rate (FDR)-adjusted p-value to 0.05 as the significance level.

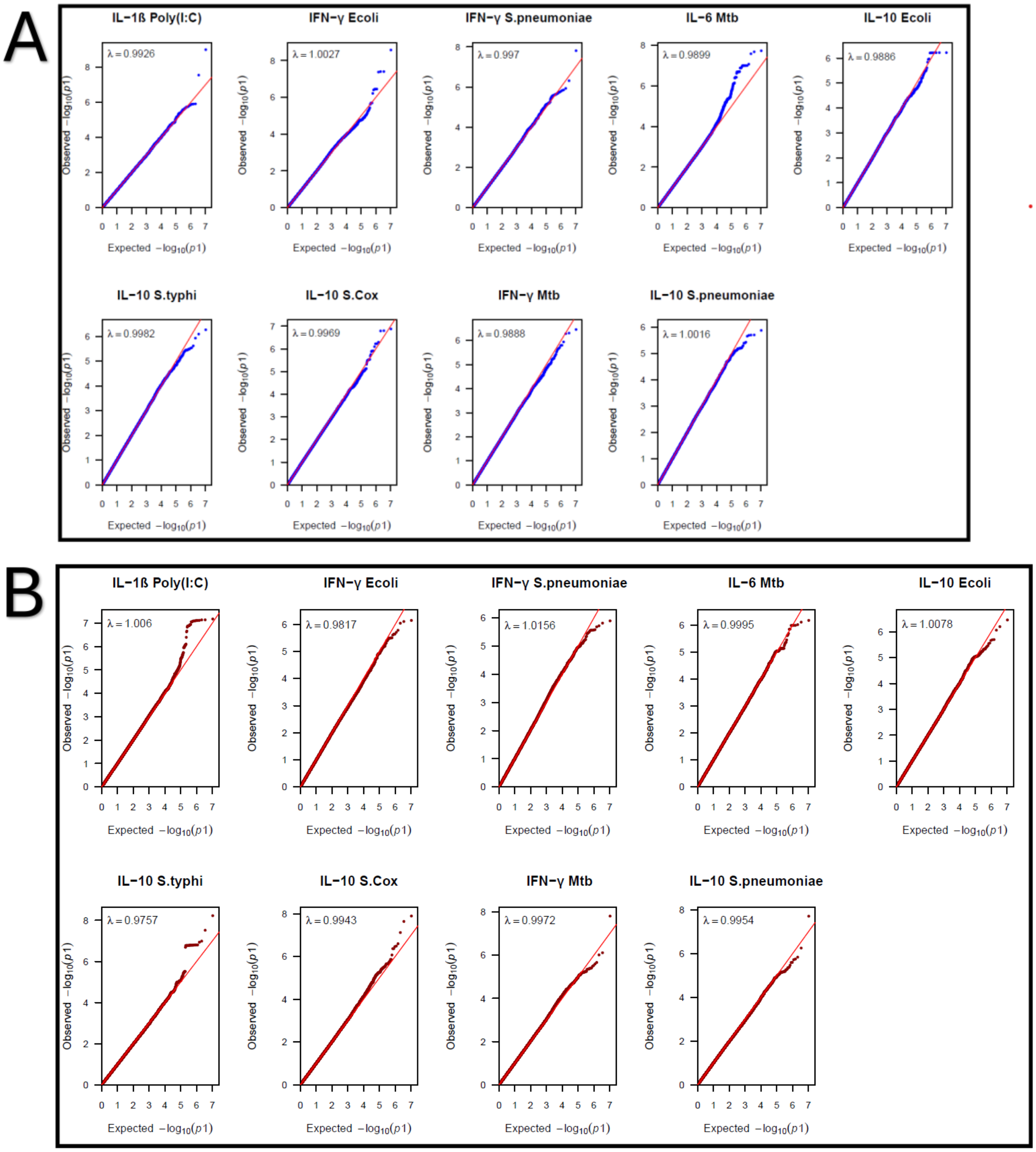

Figure S15: Quantile-quantile (Q-Q) plots for the Tanzania cohort

Quantile-quantile (Q-Q) plots for the pQTL mapping results in the Tanzania cohort for Males (A) and Females (B). The p-values distribution of the analysis (significant cytokine-stimuli) results are shown in blue and dark red colors for the Males and Females respectively.

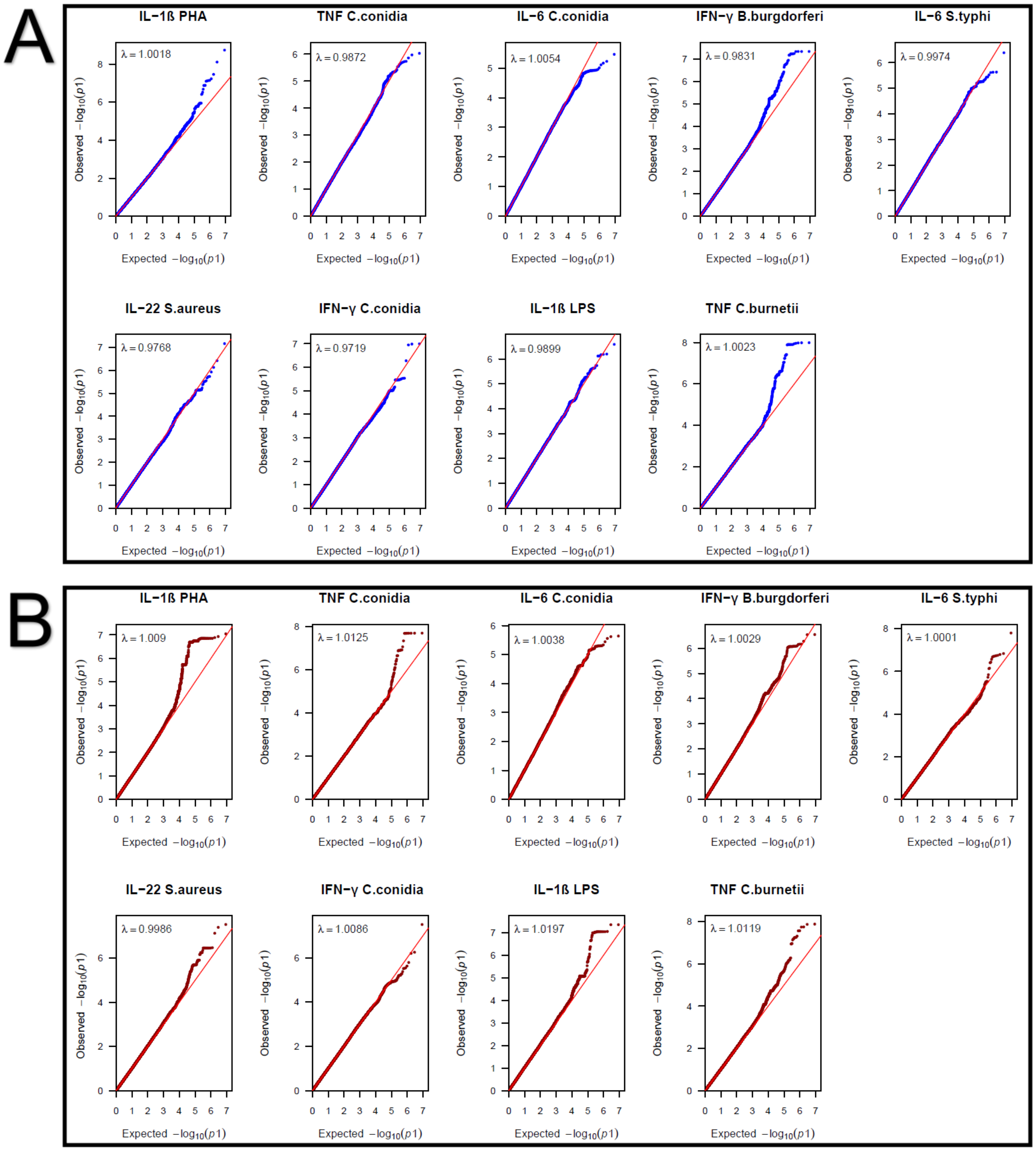

Figure S16: Quantile-quantile (Q-Q) plots for the Dutch cohort

Quantile-quantile (Q-Q) plots for the pQTL mapping results in the Dutch cohort for Males (A) and Females (B). The p-values distribution of the analysis (significant cytokine-stimuli) results are shown in blue and dark red colors for the Males and Females respectively.
